# “Dual-Boosting” Strategy to Enhance Radical Generation of Photosensitizer for Mitochondria-Targeted Phototherapy

**DOI:** 10.34133/research.1279

**Published:** 2026-05-14

**Authors:** Limin Wang, Dongming Wu, Haolin Zhang, Panpan Li, Hui Liu, Biying Zhang, Jiacong Yan, Yunxiu Li, Bo Peng, Wenbo Hu, Bin Fang, Hua Bai, Lin Li

**Affiliations:** ^1^ State Key Laboratory of Flexible Electronics (LoFE) & Institute of Flexible Electronics (IFE), Northwestern Polytechnical University, Xi’an 710072, China.; ^2^ Center of Reproductive Medicine, NHC Key Laboratory of Healthy Birth and Birth Defect Prevention in Western China, First People’s Hospital of Yunnan Province, Kunming 650500, China.; ^3^ State Key Laboratory of Flexible Electronics (LoFE) & Institute of Flexible Electronics (IFE), Xiamen University, Xiamen 361102, China.; ^4^ Future Display Institute in Xiamen, Xiamen 361005, China.

## Abstract

Organelle-targeted photodynamic therapy (PDT) shows substantial promise for precision tumor treatment. However, the clinical translation of oxygen-independent photosensitizers (PSs) designed for mitochondrial localization remains challenging. Herein, we propose a “dual-boosting” strategy to enhance the type-I PDT efficacy of mitochondria-targeted PSs. The first boost leverages multi-branched donor/π-bridge engineering to develop a series of mitochondria-targeted pyrido cyanines. Among them, McL3 displays broad visible-light absorption and a reduced singlet–triplet energy gap (Δ*E*_S1-T3_ = 0.24 eV), which collectively lead to a 4.2-fold increase in superoxide anion radical (•O_2_^−^) generation compared to McL1. The second boost is achieved through the self-assembly of McL3 with human serum albumin (HSA) into McL3@HSA nanoparticles (~40 nm). This confinement further narrows Δ*E*_S1-T2_ to 0.08 eV, amplifying •O_2_^−^ production by 20.3-fold. Mechanistic studies indicate that HSA confinement modulates molecular conformation and promotes ISC efficiency from 33% to 52%, enabling efficient •O_2_^−^ generation. Upon white-light irradiation, McL3@HSA selectively accumulates in mitochondria, inducing apoptosis and effectively inhibiting tumor growth even under hypoxic conditions. This work establishes a “dual-boosting” paradigm for the rational design of mitochondria-targeted, hypoxia-tolerant PSs, offering a promising avenue for clinical phototheranostics.

## Introduction

Photodynamic therapy (PDT) is a clinically established treatment modality used in oncology, dermatology, and ophthalmology [1–7]. In this process, photosensitizers (PSs) are activated by light to yield cytotoxic reactive oxygen species (ROS) [8–10]. Conventional type-II PSs, including hematoporphyrin, methylene blue, rose bengal (RB), and hypericin, produce singlet oxygen (^1^O_2_) via an oxygen-dependent process, which often limits their efficacy in hypoxic solid tumors [11–13]. In contrast, type-I PSs can engage in oxygen-independent electron transfer to generate radical species including superoxide anion radical (•O_2_^−^) and hydroxyl radical (•OH) [14,15], thereby offering superior potential in low-oxygen environments. Mitochondria, being central to cellular oxidative stress and energy metabolism [16], are highly promising targets for phototherapy [17]. Mitochondrial-targeted PSs generate ROS directly within the organelles, inducing immediate structural and functional damage that triggers efficient tumor cell apoptosis, thereby substantially lowering the required PS dosage and reducing systemic side effects [18–21]. Thus, developing type-I PSs capable of precise mitochondrial localization represents a powerful strategy for enhancing PDT efficacy, particularly under hypoxia.

Rational design of type-I PSs has primarily focused on enhancing intersystem crossing (ISC) and facilitating electron transfer [5,22]. In recent years, molecular engineering of electron-donating groups and π-bridges in small molecules has garnered considerable interest for its ability to suppress exciton–vibration coupling, induce red-shifted absorption, and enhance triplet-state electron transfer. Notably, the donor-π-acceptor (D-π-A) architecture promotes intramolecular charge transfer (ICT) [23,24]. Extending this to a (D-π)_n_-A framework further modulates excited-state energy dissipation pathways, enabling fine control over photophysical properties [25–27]. Concurrently, human serum albumin (HSA) is extensively used as a biocompatible nanocarrier in diagnostic and therapeutic applications [28–31]. Complexation with HSA can modulate PS conformation and excited-state properties for tumor imaging or therapy [20,25]. Nevertheless, a synergistic strategy integrating tailored molecular design with HSA confinement to specifically enhance hypoxia-resilient type-I PDT remains largely unexplored.

**Fig. 1.**
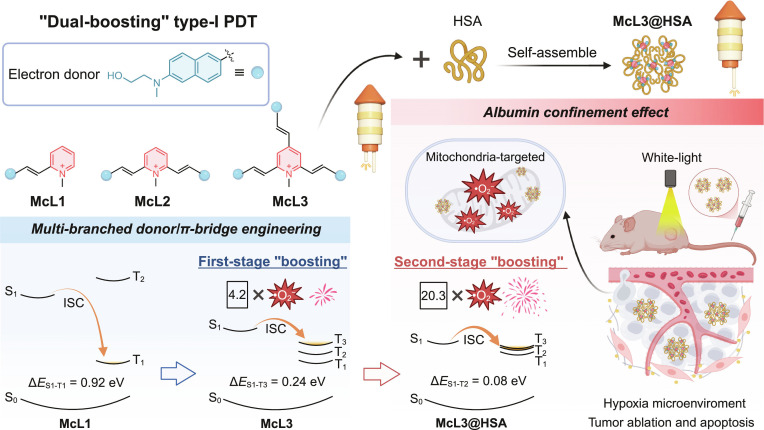
Schematic of the “dual-boosting” strategy for developing mitochondria-targeted type-I PSs. HSA, human serum albumin; •O_2_^−^, superoxide anion radical; S, singlet state; T, triplet state; ISC, intersystem crossing; Δ*E*_S-T_, singlet–triplet energy gap.

Herein, we report a “dual-boosting” strategy that integrates donor/π-bridge and albumin confinement to develop high-performance type-I PSs for mitochondria-targeted PDT (Fig. 1). We first designed a series of pyrido cyanines (McL1 to McL3) with a tunable (D-π)_n_-A (*n* = 1, 2, 3) architecture, employing *N*-methyl-*N*-hydroxyethyl naphthylamine as the donor, vinyl group as the π-spacer, and *N*-methylpyridinium as the acceptor. Increasing the number of donor/π-bridge branches effectively broadened the absorption into the visible-light region, enabling efficient white-light harvesting. Furthermore, the additional branches reduced the singlet–triplet energy gap (Δ*E*_S1-T3_ = 0.24 eV for McL3), which promoted ISC and resulted in a 4.2-fold enhancement in •O_2_^−^ generation relative to McL1. Subsequent confinement of McL3 with HSA drove its self-assembly into McL3@HSA nanoparticles, elevating •O_2_^−^ production by 20.3-fold. Mechanistic studies integrating molecular docking, excited-state dynamics, and quantum calculations revealed that HSA confinement modulates the conformation of McL3, which further narrowed Δ*E*_S1-T2_ to 0.08 eV and increased the ISC efficiency (*Φ*_ISC_) from 33% to 52%. These synergistic effects collectively facilitate efficient triplet-state electron transfer with superior type-I ROS generation. Consequently, McL3@HSA enables effective mitochondria-targeted type-I PDT, inducing cancer cell apoptosis under hypoxia conditions and inhibiting solid-tumor growth upon white-light irradiation.

## Results and Discussion

### Molecular design and photophysical properties

**Fig. 2.**
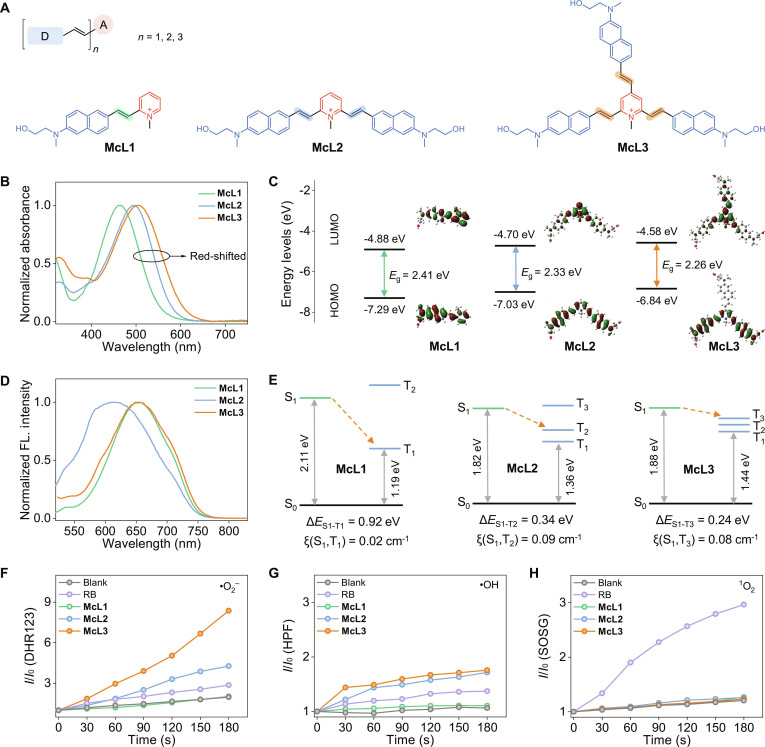
Photophysical properties and theoretical calculation of multi-branched donor/π-bridge engineered pyrido cyanines (McL1 to McL3). (A) Chemical structures of McL1, McL2, and McL3. (B) Normalized UV–vis absorption spectra of McL1 to McL3 (10 μM) in DMSO. (C) Calculated HOMO/LUMO energy levels and electron density distributions of McL1 to McL3. (D) Normalized fluorescence emission spectra of McL1 to McL3 (10 μM) in DMSO. (E) Calculated singlet (S_1_) and triplet (T_n_) energy levels, proposed S_1_ to T_n_ ISC pathways, and spin-orbit coupling (SOC) constants (ξ) for McL1 to McL3. (F to H) Relative FL intensity (*I*/*I*_0_) changes of DHR123 (λ_em_ = 524 nm), HPF (λ_em_ = 515 nm), and SOSG (λ_em_ = 527 nm) in the presence of McL1 to McL3 and rose bengal (RB) under white-light (420-nm long-pass filter, 80 mW cm^−2^) irradiation in PBS (10 mM, pH 7.42).

To elucidate the structure–property relationships, we designed a series of multi-branched donor/π-bridge to enhance ICT and promote ISC [32]. As illustrated in Fig. 1, the molecular design incorporates *N*-methyl-*N*-hydroxyethyl naphthylamine as the electron donor, vinyl groups as conformationally flexible π-bridges, and *N*-methylpyridinium as the electron acceptor. This structural configuration improves water solubility and enhances ICT to achieve red-shifted absorption, while the positively charged pyridinium moiety confers mitochondrial targeting [17]. Based on this design, a series of cationic pyrido cyanine PSs featuring a tunable (D-π)_n_-A architecture (where *n* = 1, 2, 3) were synthesized and denoted as McL1, McL2, and McL3, respectively (Fig. 2A). The synthesis employed quaternization-promoted Knoevenagel condensations to yield the desired *E*-configured products, as confirmed by the characteristic chemical shifts and coupling constants of the vinylene protons (Table S1). All synthetic compounds and key intermediates were fully characterized by ^1^H nuclear magnetic resonance (NMR), ^13^C NMR spectroscopy, and high-resolution mass spectrometry (HRMS). The characterization data are available in the Supplementary Materials (Scheme S1 and Figs. S1 to S9).

The progressive enhancement of the ICT effect with increasing donor/π-bridge branches results in a gradual red-shift in absorption. As shown in Fig. 2B, all PSs exhibit broad absorption bands in the visible region (400 to 700 nm), with absorption maxima at 461, 494, and 505 nm in dimethyl sulfoxide (DMSO), respectively. Concomitantly, the triple-branched McL3 exhibited a substantially higher molar extinction coefficient (ε = 6.2 × 10^4^ M^−1^ cm^−1^) than McL1 (ε = 2.7 × 10^4^ M^−1^ cm^−1^), indicating superior light-harvesting capability across the visible spectrum (Table S2) [33]. Density functional theory (DFT) calculations corroborate that this trend originates from a systematically reduced highest occupied molecular orbital (HOMO)-lowest unoccupied molecular orbital (LUMO) energy gap (Fig. 2C) [34], resulting from enhanced electron-donating strength and extended π-conjugation.

Emission profiles revealed distinct structure-dependent relaxation pathways in the excited state. While McL1 and McL3 emitted around 650 nm, McL2 displayed an anomalous blue-shift to 620 nm (Fig. 2D). To elucidate the underlying mechanism, we investigated the molecular conformational dynamics using variable-temperature ^1^H NMR spectra (Fig. S10). Upon heating to 328 K, the aromatic proton signals of McL2 remained well-separated, indicating a rigid framework. In contrast, the ^1^H NMR signals of McL3 coalesced at 308 K, suggesting considerable conformational flexibility. This rotational freedom arising from multiple rotatable bonds enables McL3 to readily relax into a low-energy, delocalized twisted intramolecular charge transfer (TICT) state [35], leading to red-shifted emission. Similarly, the asymmetric, single-branched McL1 can access a TICT configuration through bond rotation. In comparison, the highly symmetric and rigid structure of McL2 restricts access to such low-energy TICT conformations, thereby shifting the emission to a higher-energy, more localized excited state and resulting in the observed blue-shift.

This mechanistic interpretation is further supported by solvent- and viscosity-dependent photophysical studies. The polarity-dependent fluorescence quenching and spectral blue-shift (Fig. S11) are indicative of TICT-mediated relaxation [36]. Moreover, the viscosity spectra revealed that for all 3 compounds, fluorescence intensity increases progressively with increasing medium viscosity (Fig. S12). In addition, the fluorescence quantum yields (*Φ*_F_, Table S2) systematically decreased from 8.42% (McL1) to 0.45% (McL2) and 0.34% (McL3), indicating that increasing the number of donor branches amplifies nonradiative decay, likely through enhanced vibrational and rotational dissipation.

### Theoretical calculation and ROS production

Minimizing the singlet–triplet energy gap (Δ*E*_S-T_) offers an effective strategy for improving PS efficiency [37]. To systematically evaluate the impact of multi-branched donor/π-bridge engineering on ISC, time-dependent DFT (TD-DFT) calculations were performed. The computations revealed that increasing the donor/π-bridge branches raises the density of triplet states and systematically reduces Δ*E*_S-T_ (Fig. 2E). Subsequent analysis integrating energy gaps and spin-orbit coupling (SOC) constants (ξ) identified the dominant ISC channels: S_1_ to T_1_ for McL1 (Δ*E*_S1-T1_ = 0.92 eV), S_1_ to T_2_ for McL2 (Δ*E*_S1-T2_ = 0.34 eV), and S_1_ to T_3_ for McL3 (Δ*E*_S1-T3_ = 0.24 eV) (Table S3 and S4). The minimal Δ*E*_S-T_ value for McL3 suggests a more favorable ISC process. These computational results validate that the (D-π)_3_-A architecture in McL3 is optimal for promoting efficient ISC and offers substantial potential for enhanced ROS production.

To evaluate the ROS production of the synthesized PSs, we employed a set of commercial fluorescent probes. Specifically, •O_2_^−^, •OH, and ^1^O_2_ were detected using dihydrorhodamine 123 (DHR123), hydroxyphenyl fluorescein (HPF), and singlet oxygen sensor green (SOSG), respectively [18,37,38]. As shown in Fig. 2F and G, increasing the number of donor/π-bridge branches led to a gradual enhancement in the fluorescence intensities of DHR123 and HPF under white-light irradiation (Figs. S13 and S14). In contrast, the negligible fluorescence intensity changes in SOSG indicated minimal ^1^O_2_ production (Fig. 2H and Fig. S15). Moreover, the fluorescence of DHR123 and HPF was effectively quenched upon the introduction of the radical scavengers vitamin C (VC; for •O_2_^−^) and isopropyl alcohol (IPA; for •OH) [11,39], which further confirmed the specific •O_2_^−^ and •OH generation of McL3 via a type-I pathway (Fig. S16). Notably, the •O_2_^−^ generation of McL3 was markedly higher than that of McL1 and McL2, as well as the traditional type-II PS, RB [40].

These findings highlight the efficacy of the multi-branched donor/π-bridge design in promoting electron transfer-driven type-I phototheranostics. In particular, McL3, with its red-shifted absorption, enhanced molar extinction coefficient, and minimized Δ*E*_S-T_, demonstrated the most efficient generation of •O_2_^−^ and •OH via a type-I mechanism, aligning with the theoretically predicted trends.

### HSA confinement to modulate ROS generation

To enhance biocompatibility and promote excited-state electron transfer, we introduced HSA to form self-assembled nanoparticles with McL3, thereby further boosting ROS production. We first examined the responsiveness of the PSs (McL1 to McL3) to HSA. While the absorption spectra remained basically unchanged with increasing HSA concentration (Fig. S17), all PSs exhibited enhanced fluorescence intensity and brightness (Table S5). Moreover, binding to HSA substantially prolonged fluorescence lifetime (Fig. S18), indicating an extended excited-state duration, consistent with our previously proposed confinement fluorescence effect (CFE) [41].

**Fig. 3.**
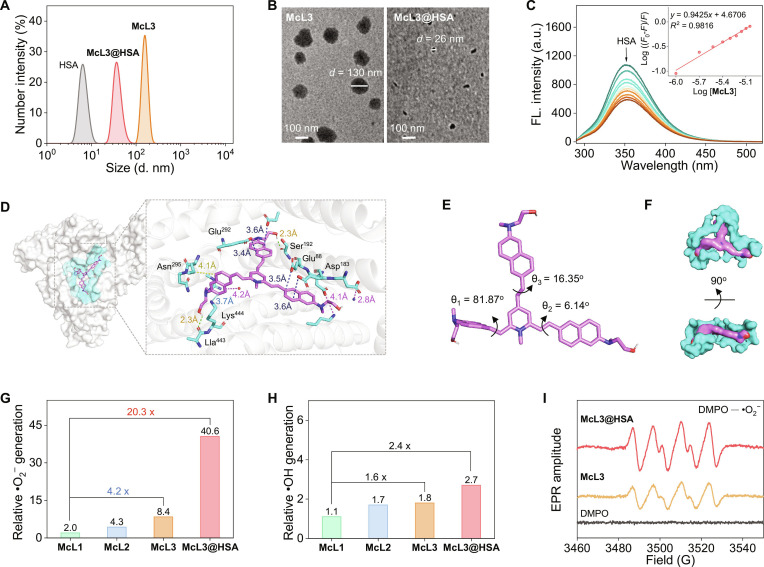
Albumin confinement enhances ROS generation. (A) Hydrodynamic size distribution profiles and (B) corresponding transmission electron microscopy (TEM) micrographs of McL3 and McL3@HSA. (C) Fluorescence emission spectra (λ_ex_ = 280 nm) of HSA (10 μM) upon titration with McL3 in PBS (10 mM, pH 7.42). (Insert) Double-logarithmic regression plot used to determine the binding constant (*K*_a_ > 10^5^ M^−1^) and binding stoichiometry (1:1). (D) Molecular docking model of McL3 (magenta, stick) within the binding pocket (cyan, surface) of HSA (PDB: 4lb9). (Left) Overall binding pose. (Right) Detailed view of key interacting residues. (E) Configuration of McL3 within the HSA binding pocket. The dihedral angle θ is defined between the planes of the donor (naphthylamine) and acceptor (pyridinium) moieties. (F) Surface representations of the McL3@HSA from 2 orthogonal views. (G and H) Comparative •O_2_^−^ and •OH generation abilities of McL1 to McL3 and McL3@HSA measured by the fluorescence increase of DHR123 and HPF after 180-s white-light irradiation (420-nm long-pass filter, 80 mW cm^−2^). Data are expressed as fold change in fluorescence intensity. (I) Electron paramagnetic resonance (EPR) signals of McL3 and McL3@HSA under white-light irradiation, using DMPO as a spin trap for •O_2_^−^.

The interaction of McL3 with HSA was characterized by dynamic light scattering (DLS) and transmission electron microscopy (TEM). DLS confirmed the formation of nanoparticles with a hydrodynamic diameter of approximately 40 nm (Fig. 3A), a size favorable for tumor accumulation via the enhanced permeability and retention effect [42]. TEM further revealed a morphological transition, with McL3@HSA forming well-defined, porous nanoparticles averaging 26 nm in diameter (Fig. 3B). The observed size difference between DLS and TEM is attributed to sample dehydration during TEM preparation [22,43]. Moreover, McL3@HSA exhibited good colloidal stability in phosphate-buffered saline (PBS) solution (Fig. S20). These results collectively demonstrate the successful self-assembly of McL3 and HSA into stable and monodisperse nanoparticles.

To elucidate the configuration and interaction between McL3 and HSA, fluorescence titration and molecular docking studies were performed. As shown in Fig. 3C, McL3 binds to HSA with a high affinity (*K*_a_ > 10^5^ M^−1^) and a 1:1 stoichiometry (Table S5), indicating strong molecular interaction [44]. Molecular docking simulations were subsequently carried out to investigate the binding mode [29]. As illustrated in Fig. 3D, McL3 (magenta sticks) localizes deeply within a cyan-colored binding pocket of HSA [Protein Data Bank (PDB): 4lb9]. Among 9 simulated binding poses, the most favorable configuration exhibited a binding energy of −10.6 kcal/mol (Table S6). Detailed analysis revealed that McL3 embeds into a protein subpocket formed by residues including Glu^292^, Asn^295^, Lys^444^, Lla^443^, Ser^192^, Glu^88^, and Asp^183^, exhibiting high shape complementarity with the HSA surface. Specific interactions occur between protein residues and the naphthylamine donor group of McL3, with intermolecular distances around 4 Å, and stabilize the bound conformation (Fig. 3D, right), rationalizing the enhanced photophysical properties upon nanocomplex formation. The docking results further revealed that McL3 adopts a bird-shaped twisted conformation within the HSA binding pocket (Fig. 3E). One naphthylamine electron donor branch forms a near-vertical dihedral angle (θ = 82^o^) relative to the pyridinium core, while the remaining branches are virtually coplanar. Overall, these simulations confirm that HSA effectively traps McL3 within a tailored subpocket (Fig. 3F), contributing to the formation of McL3@HSA complex.

We subsequently quantified the ROS generation of the HSA-assembled nanoparticles (Figs. S13 to S15). Comparative analysis revealed that both multi-branched donor/π-bridge engineering and the albumin confinement assembly enhanced ROS generation efficiency (Fig. 3G and H and Fig. S22). Among the PSs, ROS production increased with the number of donor branches, and McL3 exhibited a 4.2-fold higher •O_2_^−^ production than did McL1. Subsequent HSA confinement further amplified ROS generation, with McL3@HSA showing a 20.3-fold increase in •O_2_^−^ generation (Fig. 3G) and a 2.4-fold enhancement in •OH production (Fig. 3H) over McL1. These results confirm that the “dual-boosting” strategy synergistically amplifies the generation of ROS. Electron paramagnetic resonance (EPR) spectroscopy provides direct verification of the enhanced •O_2_^−^ production [45]. Under white-light irradiation, McL3@HSA exhibited markedly stronger 5,5-dimethyl-1-pyrroline N-oxide (DMPO)–•O_2_^−^ adduct signals than McL3 (Fig. 3I). This finding corroborates prior fluorescent probe and radical scavenging experiments (Fig. S16), further confirming the type-I PDT mechanism of McL3@HSA with efficient •O_2_^−^ generation. Additionally, McL3@HSA exhibited excellent photostability under physiological environments (Fig. S24). Collectively, these results demonstrate that the enhanced type-I ROS generation and superior photostability of McL3@HSA are promising for hypoxia-tolerant phototherapy.

### Excited-state dynamics analysis

**Fig. 4.**
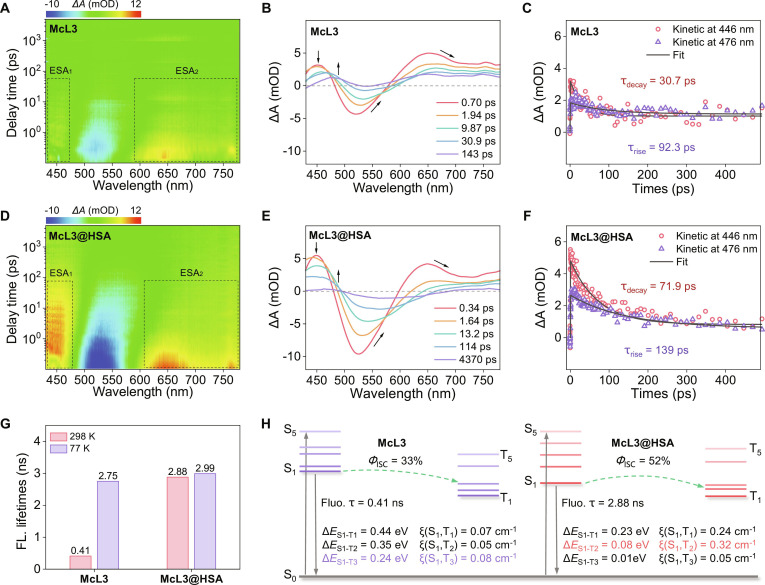
Experimental and computational insights into ISC enhancement. (A and D) Two-dimensional pseudo-color *fs*-TA maps of McL3 and McL3@HSA in PBS (10 mM, pH 7.42) upon photoexcitation at 400 nm. (B and E) Corresponding *fs*-TA traces at selected delay times. (C and F) Kinetic traces and fitted curves for McL3 and McL3@HSA at representative wavelength, yielding the rise (τ_rise_) and decay (τ_decay_) lifetimes. (G) Fluorescence lifetimes of McL3 and McL3@HSA at 298 and 77 K. (H) Schematic energy-level diagrams illustrating the proposed ISC pathways from S_1_ to triplet states (T_n_) for McL3 and McL3@HSA, highlighting the relevant S_1_-T_n_ energy gaps (Δ*E*_S-T_) and spin-orbital coupling (SOC) constants.

To elucidate the origin of the enhanced ROS generation in McL3@HSA, we performed femtosecond transient absorption (*fs*-TA) spectroscopy [46,47]. The time-resolved spectra of McL3 and its HSA complex McL3@HSA provide kinetic and spectral insights into their excited-state processes (Fig. 4A and D). A negative band between 490 and 560 nm is attributed to ground-state bleach (GSB) and stimulated emission (SE), and 2 positive excited-state absorption (ESA) signals at 450 nm (ESA_1_) and 650 nm (ESA_2_) belong to excited singlet and triplet absorption. Temporal evolution profiles extracted at selected delays revealed that the excited-state signal of McL3 decayed within 143 ps (Fig. 4B), whereas that of McL3@HSA persisted beyond 4,370 ps (Fig. 4E), indicating heavily prolonged excited-state lifetime due to HSA binding.

Kinetic analysis provided further insight into the ISC process. At longer delay times, both McL3 and McL3@HSA exhibited a long-lived species around 480 nm, accompanied by the decay of signals around 450 nm. The observed isosbestic point between 450 and 480 nm indicates spectral evolution from the S_1_ to T_1_ state associated with ISC [48]. Further analysis of the dynamics yielded lifetimes for McL3, a decay component of 30.7 ps (446 nm, S_1_ decay), and a corresponding rise component of 92.3 ps (476 nm, T_1_ formation) (Fig. 4C). For McL3@HSA, these components were 71.9 ps (decay) and 139 ps (rise) (Fig. 4F). From these values, the ISC efficiency (*Φ*_ISC_ = [1/τ (T_1_, rise)]/[1/τ (S_1_, decay)]) was estimated to be 33% for McL3 and 52% for McL3@HSA. These results demonstrate that HSA confinement effectively enhances ISC efficiency, thereby promoting more efficient ROS generation.

We further validated the restriction of the cavity structure of HSA on the rotational freedom of the π-bridge in McL3, stabilizing specific twisted molecular conformations. Both the convergence of emission peaks for McL3 (580 nm) and McL3@HSA (590 nm) at 77 K (Fig. S25) and the close match between the low-temperature fluorescence lifetime of McL3 (2.75 ns; Fig. S26A) and that of McL3@HSA at room temperature (2.88 ns; Fig. S18C) strongly support the role of HSA in conformationally restraining McL3 (Fig. 4G). A blue-shift in the emission peak of McL3 under both low-temperature and protein confinement conditions further indicated that suppressed thermal vibration enhances the radiative transition rate. These results confirm that the HSA protein cavity effectively stabilizes the conformation of McL3.

To complement the experimental kinetics, we calculated the excited-state energy levels for McL3@HSA. As illustrated in Fig. 4H, the twisted conformation induced by HSA binding reduces the energy gap between S_1_ and T_2_ to 0.08 eV, compared with the S_1_-T_3_ gap of 0.24 eV in McL3 (Table S3). This is accompanied by a substantial increase in the relevant SOC constant from 0.08 cm^−1^ to 0.32 cm^−1^ (Table S4). The combined effect of a reduced Δ*E*_S-T_ and a strengthened SOC provides a solid theoretical foundation for the dramatically improved ISC [40] and ROS generation observed experimentally for McL3@HSA.

These results establish that HSA confinement promotes ISC by stabilizing the molecular conformation to reduce Δ*E*_S-T_, promoting the type-I PDT pathway. The “dual-boosting” strategy, integrating multi-branched donor/π-bridge engineering with albumin confinement, narrows the singlet–triplet energy gap and enhances ISC efficiency to amplify ROS generation, offering a versatile platform for high-performance phototheranostics.

### Mitochondria-targeted in vitro phototherapy

**Fig. 5.**
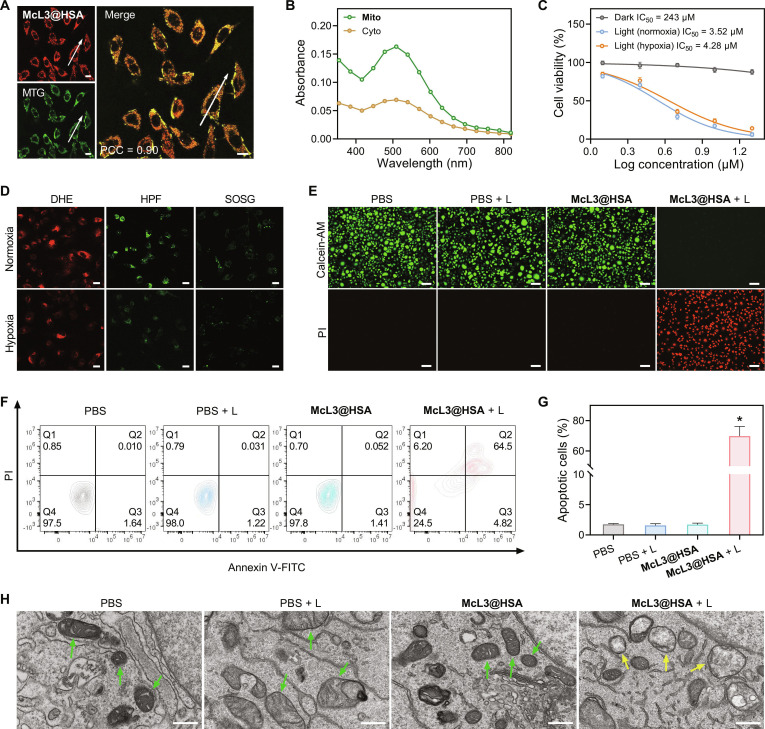
In vitro mitochondrial colocalization and cytotoxicity evaluation. (A) Fluorescence colocalization images of McL3@HSA (λ_ex/em_ = 488/550 to 750 nm) costained with MitoTracker Green (MTG; λ_ex/em_ = 488/500 to 540 nm) in A549 cells. Inset: Pearson correlation coefficient (PCC). Scale bar, 10 μm. (B) Absorption spectra of McL3@HSA in mitochondrial and cytoplasmic fractions. (C) Cell viability of A549 cells treated with different concentrations of McL3@HSA in the dark or irradiated with white light (L) (420-nm long-pass filter, 80 mW cm^−2^, 10 min) under normoxia or hypoxia. Data were presented as mean ± SD (*n* = 6). (D) Intracellular ROS detection of McL3@HSA (1.0 μM) treated with DHE (λ_ex/em_ = 488/560 to 620 nm), HPF (λ_ex/em_ = 488/500 to 540 nm), and SOSG (λ_ex/em_ = 488/500 to 540 nm) in A549 cells. Scale bar, 20 μm. (E) Live/dead staining (Calcein-AM, green; PI, red) of A549 cells under hypoxia after various treatments (AM, λ_ex/em_ = 488/500 to 550 nm; PI = 561/580 to 630 nm). Scale bar, 100 μm. (F) Flow cytometric analysis of apoptosis (Annexin V-FITC/PI) under hypoxia. (G) Quantification of apoptotic cells from (F). Data were presented as mean ± SD (*n* = 3). Statistical significance: *P < 0.05 versus PBS group. (H) TEM images of mitochondria in A549 cells under hypoxia after different treatments. Green arrows indicate intact mitochondrial membranes with well-defined cristae; yellow arrows indicate condensed membranes and distorted cristae. Scale bar, 500 nm.

To evaluate the cellular ROS generation capability of McL3@HSA, we first investigated its intracellular distribution using confocal microscopy. Strong colocalization of McL3@HSA with MitoTracker Green (MTG) was observed, yielding a high Pearson’s correlation coefficient (PCC) of 0.90 (Fig. 5A). The fluorescence intensity profiles further confirmed successful internalization and mitochondria-specific accumulation (Fig. S27). Furthermore, control experiments showed minimal overlap with nuclear (Hoechst 33342) or lysosomal [LysoTracker Green (LTG)] markers (PCC = 0.03 and 0.10, respectively; Fig. S27), confirming the mitochondria-targeting specificity of McL3@HSA. We next investigated the endogenous enrichment of McL3@HSA in mitochondria. After incubating A549 cells with McL3@HSA, mitochondrial and cytoplasmic fractions were isolated by differential centrifugation. The absorbance of McL3@HSA in the mitochondrial fraction was higher than that in the cytoplasmic fraction (Fig. 5B), indicating effective mitochondrial enrichment.

We next evaluated the therapeutic potential of McL3@HSA. Using a cell counting kit-8 (CCK-8) assay [8,11], we assessed both dark cytotoxicity and photocytotoxicity under normoxic (21% O_2_) and hypoxic (2% O_2_) conditions. McL3@HSA showed low dark cytotoxicity, with cell viability maintained above 85% (Fig. S30), confirming high biocompatibility. Upon white-light (L) irradiation, McL3@HSA induced pronounced and dose-dependent phototoxicity (Fig. 5C), with a median inhibitory concentration (IC_50_) values of 3.52 μM under normoxia and 4.28 μM under hypoxia. Additionally, the photocytotoxicity index [IC_50(dark)_/IC_50(light)_] [49] was determined to be 57-fold under hypoxia conditions (Table S7), demonstrating its promising potential in the hypoxia microenvironment.

Intracellular ROS generation was further monitored using the fluorescent probes dihydroethidium (DHE) (for •O_2_^−^), HPF (for •OH), and SOSG (for ^1^O_2_). Under both normoxia and hypoxia, the fluorescence intensities of DHE and HPF increased upon light irradiation, indicating efficient production of •O_2_^−^ and •OH, respectively (Fig. 5D). In comparison, the fluorescence signal of SOSG remained weak, suggesting minimal ^1^O_2_ generation. Notably, the fluorescence signals from DHE and HPF were not markedly suppressed under hypoxia conditions (Fig. S31), further supporting the hypoxia tolerance of McL3@HSA as a type-I PS.

To elucidate the phototherapy efficacy, live/dead cell staining was performed using Calcein-AM and propidium iodide (PI) [49]. As depicted in Fig. 5E, nearly all cells in the therapeutic group (McL3@HSA + L) exhibited red fluorescence upon white-light irradiation, whereas the control groups (PBS, PBS + L, and McL3@HSA) displayed predominantly green fluorescence. Furthermore, the phototherapeutic efficacy of McL3@HSA under hypoxic conditions was markedly superior to that of the type-II PS RB (Fig. S32). Quantitative validation of apoptosis was obtained through flow cytometry with Annexin V–fluorescein isothiocyanate (FITC)/PI staining. As shown in Fig. 5F and G, the therapeutic group displayed a pronounced shift toward apoptosis, with over 69% of cells in early and late apoptotic stages (Q2 + Q3), whereas the control groups maintained approximately 98% viability (Q4). These results are fully aligned with the CCK-8 and live/dead staining assays.

Given the excellent mitochondrial targeting capability and superior ROS generation ability of McL3@HSA, which enables direct oxidative damage to induce apoptosis, we further investigated its effect on mitochondrial morphology using TEM (Fig. 5H). After incubation of McL3@HSA with A549 cells, mitochondria exhibited intact membranes and well-defined cristae in the absence of light. Upon white-light irradiation, mitochondria displayed severely condensed membranes and distorted cristae. These morphological changes indicate that McL3@HSA induces mitochondrial damage upon light exposure. Collectively, these findings demonstrate that McL3@HSA, as a potent type-I PS, effectively induces tumor cell death even under hypoxic conditions, thereby overcoming a critical limitation of conventional phototherapy and highlighting its potential for clinical translation.

### Fluorescence imaging and in vivo phototherapy

**Fig. 6.**
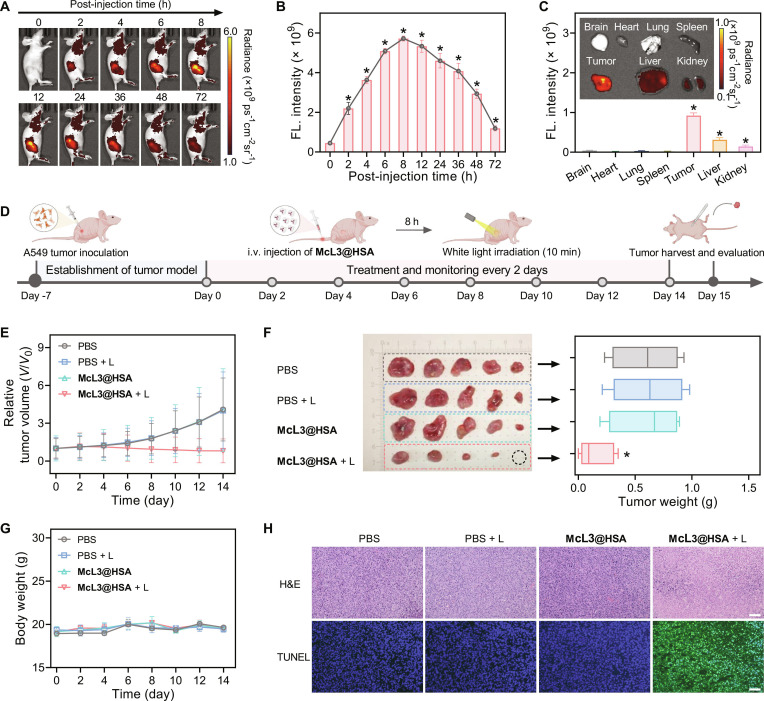
Assessment of in vivo tumor targeting and phototherapy. (A) Time-dependent in vivo fluorescence imaging post-injection of McL3@HSA (80 μl, 200 μM). (B) Corresponding quantification of tumor signal intensity. Data were presented as mean ± SD (*n* = 3). Statistical significance: *P < 0.05 versus 0 h group. (C) Ex vivo fluorescence images (inset) and quantitative analysis of major organs and tumors harvested 24 h post-injection. Data were presented as mean ± SD (*n* = 3). Statistical significance: *P < 0.05 versus brain group. (D) Schematic illustration of A549 tumor model transplantation and phototherapy protocol. i.v., intravenous. (E) Tumor volume growth curves for the indicated groups. (F) Photograph (left) and weights (right) of excised tumors after the final treatment. (G) Body weight monitoring throughout the study. (H) Representative H&E and TUNEL analysis of tumor tissues. Scale bar, 50 μm. Data were presented as mean ± SD (*n* = 5). Statistical significance: *P < 0.05 versus PBS group.

Given the promising in vitro results, the in vivo performance of McL3@HSA was further evaluated in A549 subcutaneous transplantation mouse models [50]. Fluorescence imaging after intravenous injection showed time-dependent tumor accumulation, with the signal peaking at 8 h post-injection (Fig. 6A), defining the optimal treatment window. The signal persisted at the tumor site for up to 72 h, indicating prolonged retention (Fig. 6B). Ex vivo imaging of the organs was performed at 24 h. The strongest fluorescence was detected in the tumor, while moderate signals were present in the liver and kidneys, suggesting clearance via hepatobiliary and renal pathways (Fig. 6C). Pharmacokinetic analysis revealed a blood circulation half-life [20] of 26 h for McL3@HSA (Fig. S33). Furthermore, fecal fluorescence tracking confirmed its gradual excretion over 72 h (Fig. S34), supporting favorable metabolic stability and biosafety.

We next evaluated the antitumor efficacy of McL3@HSA mediated by white-light irradiation in A549 tumor-bearing mice (Fig. 6D). BALB/c nude mice were randomly divided into 4 treatment groups: PBS, PBS + L, McL3@HSA, McL3@HSA + L. At 8 h post-intravenous injection of McL3@HSA, mice in the irradiation groups were exposed to white light for 10 min. Tumor volumes and body weights were monitored and measured every 2 d. As shown in Fig. 6E, tumor growth in the McL3@HSA + L group was markedly inhibited compared with that in all control groups (Fig. S35). On day 15, excised tumors from the McL3@HSA + L group weighed only 26% of those in the PBS group, confirming its potent antitumor efficacy (Fig. 6F). Importantly, no obvious body weight loss was observed in any group throughout the study (Fig. 6G), indicating minimal systemic toxicity.

To further validate treatment effects at the histological level, tumor sections were analyzed by hematoxylin and eosin (H&E) and terminal deoxynucleotidyl transferase-mediated deoxyuridine triphosphate nick end labeling (TUNEL) staining. As shown in Fig. 6H, the McL3@HSA + L group exhibited extensive tumor cell apoptosis and nuclear fragmentation; in contrast, no discernible damage was observed in the other groups. Furthermore, H&E analysis of major organs (heart, liver, spleen, lung, kidney) revealed no pathological alterations in any treatment group (Fig. S36). These data collectively highlight the excellent biocompatibility of McL3@HSA, underscoring its translational promise for phototheranostics.

## Conclusion

In summary, we developed a high-performance mitochondria-targeted type-I PS through a “dual-boosting” strategy that integrates multi-branched donor/π-bridge engineering with albumin confinement effect. Initially, molecular engineering yielded McL3 with (D-π)_3_-A structure that exhibits enhanced visible-light absorption and a reduced singlet–triplet energy gap (Δ*E*_S1-T3_ = 0.24 eV), boosting •O_2_^−^ generation 4.2-fold relative to McL1. Subsequently, self-assembly with HSA formed the McL3@HSA nanoparticles. Within the protein cavity, twisted conformation of McL3 further narrows Δ*E*_S1-T2_ to 0.08 eV and amplifies •O_2_^−^ production by 20.3-fold. Transient absorption spectroscopy confirmed that HSA binding restrains molecular motion, elevating the ISC efficiency from 33% (McL3) to 52% (McL3@HSA), thereby enabling efficient type-I ROS generation. In vitro, McL3@HSA selectively accumulates in mitochondria, upon white-light irradiation, effectively inducing apoptosis even under hypoxic conditions. In vivo studies further demonstrated effective tumor photoablation via fluorescence imaging-guided phototherapy, with no observable systemic toxicity. This “dual-boosting” strategy establishes a generalizable design paradigm for high-performance type-I phototheranostics with clinical translation potential.

## Materials and Methods

### General information

All commercial chemicals and solvents were used as supplied. DMSO, methanol (MeOH), ethanol (EtOH), *N*,*N*-dimethylformamide (DMF), acetonitrile (ACN), tetrahydrofuran (THF), dichloromethane (DCM), and HSA were purchased from Sigma-Aldrich (USA). PBS was purchased from Servicebio Technology. Dulbecco’s modified Eagle’s medium (DMEM), fetal bovine serum (FBS), and fluorescent probes/kits (Hoechst 33258, MTG, LTG, Calcein-AM/PI, CCK-8) were provided by Beyotime (China). ROS detection probes DHR123, DHE, and HPF were sourced from Shanghai Maokang Biotechnology Co. (China); SOSG was purchased from Thermo Fisher (USA). Reactions were monitored by TLC F254 plates (glass-backed, 250-μm thickness), with spots visualized under a portable ZF-7 ultraviolet (UV) lamp. Flash column chromatography was carried out using silica gel (mesh 200-300) at room temperature under positive pressure.

### TD-DFT calculation

Computational studies were carried out using Gaussian 16 [51] and ORCA 6.0.1 [52]. Ground-state geometry optimization was performed at the B3LYP/def2-SVP level of theory [53]. Based on the optimized structures, time-dependent density functional theory (TD-DFT) calculations were conducted to obtain the energies of singlet (S_n_) and triplet (T_n_) states and their corresponding energy gaps (Δ*E*_S-T_). The spin-orbital coupling (SOC) constants between these states were derived from the ORCA quantum chemistry program package.

### Molecular docking

The binding conformation of McL3 with HSA (PDB: 4LB9) was predicted by molecular docking [20]. The HSA structure was prepared by removing the cocrystallized ligand and solvent molecules. The ligand structure was optimized and docked into the defined binding site (grid center: 70.19, 29.85, and 104.22 nm) using AutoDock Vina. Results were visualized with Discovery Studio.

### Calculation for binding affinity

Binding affinities were predicted by molecular docking (AutoDock Vina) [20]. The HSA structure (PDB: 4LB9) and the ligand were prepared with MGLTools. A global docking search covering the entire protein volume was performed, with 100 independent runs to identify the optimal binding pose and its associated affinity score.

### The fluorescence quantum yields

Fluorescence quantum yields (*Φ*_F_) were determined using the relative method with rhodamine B (*Φ*_r_ = 0.31 in water) [54] as the standard. The quantum yield was calculated according to the following standard formula: *Φ_s_* = *Φ_r_*(*A_r_F_s_n_s_*^2^)/(*A_s_F_r_n_r_*^2^), where *A* is the absorbance at excitation, *F* is the integrated fluorescence intensity, *n* is the solvent refractive index, and the sample and reference are denoted by the subscripts *s* and *r*, respectively.

### The fluorescence lifetimes

Fluorescence lifetimes were acquired via time-correlated single photon counting (TCSPC) on an FLS1000 spectrometer using 450-nm pulsed excitation and monitoring at the emission maximum wavelength. Decay profiles were analyzed with a biexponential function [55]: *I*_(*t*)_ = *A*_1_exp[(−*t*)/*τ*_1_] + *A*_2_exp[(−*t*)/*τ*_2_], and the average lifetime was derived from *τ* = (*A*_1_*τ_1_*^2^ + *A*_2_*τ*_2_^2^)/(*A*_1_*τ*_1_ + *A*_2_*τ*_2_), where *I*_(*t*)_ is the fluorescence intensity at time *t*, and *A*_1_ and *A*_2_ are the amplitudes of the lifetime components *τ*_1_ and *τ*_2_.

### Detection of superoxide anion radical (•O_2_^−^)

The •O_2_^−^ was performed by DHR123 as specific indicator. DHR123 stock solution (10 mM in DMSO) was diluted to 5 μM in aqueous solution and mixed with samples or with commercial PS RB (absorbance adjusted to 0.2), and fluorescence emission spectra were acquired at specified intervals during light exposure. For radical scavenging experiments, VC (200 μM) was employed as •O_2_^−^ scavenger [11].

### Detection of hydroxyl radical (•OH)

The •OH generation was measured by HPF as an indicator. HPF stock solution (5 mM in DMF) was diluted to 5 μM in aqueous solution and mixed with the sample or RB (absorbance adjusted to 0.2), and fluorescence emission spectra were acquired at specified intervals during light exposure. For radical scavenging experiments, IPA (250 μM) was employed as •OH scavenger [39].

### Detection of singlet oxygen (^1^O_2_)

The ^1^O_2_ generation was detected using SOSG as an indicator. SOSG stock solution (10 mM in DMSO) was diluted to 5 μM in aqueous solution and mixed with the sample or RB (absorbance adjusted to 0.2), and fluorescence emission spectra were acquired at specified intervals during light exposure.

### EPR spectroscopy measurement

EPR spectroscopy was employed to detect •OH and •O_2_^−^ using DMPO as a spin trap. Sample solutions containing the test agent (50 μM) and DMPO (100 mM) were prepared. The mixture was then irradiated with white light (420-nm long-pass filter, 80 mW cm^−2^) for 5 min, and the EPR spectroscopy was observed immediately after irradiation.

### Cell cultures

A549 cells were supplied by the American Type Culture Collection (ATCC). Cells were grown in DMEM that contained 10% FBS, along with penicillin (100 U/ml) and streptomycin (100 μg/ml). For normoxic culture, cells were incubated at 37 °C in a humidified atmosphere of 21% O_2_ and 5% CO_2_. Hypoxic culture (2% O_2_, 5% CO_2_) was established using a dedicated hypoxic incubator chamber (AIPUINS) to simulate the tumor microenvironment.

### Living cell fluorescence imaging

A549 cells were seeded in 35-mm glass-bottom dishes and grown to 60% to 70% confluence in complete DMEM. For colocalization analysis, cells were first incubated with McL3@HSA (1 μM) for 1 h and then stained with Hoechst 33342 (10 μg/ml), MTG (100 nM), and LTG (100 nM) for 30 min. Subsequently, cells were washed 3 times with PBS and maintained in phenol red-free DMEM for imaging. Fluorescence images were acquired on a Nikon C2 confocal microscope using the following excitation/emission settings: McL3@HSA (488/550 to 750 nm), Hoechst (405/440 to 480 nm), and MTG and LTG (488/500 to 540 nm). Colocalization analysis and intensity quantification were performed using ImageJ software.

### Mitochondrial enrichment analysis

A549 cells (5 × 10^6^ cells per well) were incubated with McL3 or McL3@HSA at a concentration of 10 μM for 4 h (control cells were treated without the compounds). Cells were then collected, and the mitochondrial and cytoplasmic fractions were isolated by differential centrifugation. Protein concentrations were normalized using a bicinchoninic acid (BCA) protein assay kit (Pierce, Rockford, IL), and the absorbance of each fraction was measured by a UV–visible (UV–vis) absorption spectrophotometer.

### Cell viability and cytotoxicity assay

A549 cells were seeded in 96-well plates at 5 × 10^4^ cells per well. After 24 h of incubation, the medium was replaced with 100 μl of fresh medium supplemented with McL3@HSA at a series of concentrations (0 to 20 μM). After 4 h of incubation, the plates were divided into 2 sets: One set was irradiated with white light (420-nm long-pass filter, 80 mW cm^−2^) for 10 min, while the other set was kept in the dark as a control. After irradiation, all plates were incubated at 37 °C for an additional 4 h. Subsequently, the medium was replaced with 100 μl of fresh serum-free medium containing CCK-8 (10 μl) and incubated at 37 °C for 1 h. Absorbance at 450 nm was detected using a Spark multi-mode plate reader. Cell viability was determined according to the following equation: Viability (%) = (*A_s_* − *A_b_*)/(*A_c_* − *A_b_*) × 100%, where *A_s_*, *A_b_*, and *A_c_* represent the absorbance of the treated wells, blank wells (medium plus CCK-8 without cells), and untreated control wells, respectively.

### Intracellular ROS detection

A549 cells were seeded into 35-mm confocal dishes and preincubated for 12 h under either normoxic or hypoxic conditions. Cells were then treated with McL3@HSA (1 μM) for 1 h. To detect specific ROS, cells were subsequently incubated for 30 min with one of the following fluorescent probes: DHE (5 μM) for •O_2_^−^, HPF (5 μM) for •OH, and SOSG (5 μM) for ^1^O_2_. Before light exposure, the probe-containing medium was refreshed. Cells were then irradiated with white light (420-nm long-pass filter, 80 mW cm^−2^, 5 min); following a PBS wash, confocal imaging was performed using a Nikon C2 confocal microscope. Fluorescence signals were collected at 560 to 620 nm for DHE and at 500 to 540 nm for HPF and SOSG, with all probes excited at 488 nm. Fluorescence intensity was quantified using ImageJ software.

### Live/dead cell staining assay

Cell viability was further assessed using a Calcein-AM/PI double-staining kit. Calcein-AM produces green fluorescence in live cells, whereas PI stains dead cells with red fluorescence. A549 cells were seeded in 12-well plates at 5 × 10^4^ cells per well and incubated for 24 h. Cells were then treated with McL3@HSA (5 μM) for 4 h and divided into 2 groups: One group was irradiated with white light (420-nm long-pass filter, 80 mW cm^−2^, 10 min), and the other was kept in the dark as a control. Subsequently, all cells were incubated for an additional 6 h. After washing with PBS, cells were stained with the Calcein-AM/PI mixture according to the manufacturer’s instructions for 30 min at room temperature in the dark, followed by 3 PBS washes. Imaging was performed on an EVOS FL Auto 2 inverted fluorescence microscope using the following excitation/emission settings: Calcein-AM (488 nm/500 to 550 nm) and PI (561 nm/580 to 630 nm).

### Flow cytometry analysis of apoptotic cells

A549 cells (2 × 10^5^ cells per well in 12-well plates) were treated with McL3@HSA (5 μM) for 4 h and then exposed to white light (420-nm long-pass filter, 80 mW cm^−2^, 10 min) or kept in the dark. Then, cells were allowed to incubate at 37 °C for another 12 h, harvested, stained with Annexin V-FITC/PI according to the protocols of the manufacturer, and analyzed immediately by flow cytometry (CytoFLEX).

### TEM imaging of mitochondria

A549 cells were incubated with McL3@HSA (5 μM) for 4 h and then exposed to white light (420-nm long-pass filter, 80 mW cm^−2^, 10 min) or kept in the dark. Following treatment, cells were further incubated for an additional 4 h and then harvested and fixed in 2.5% glutaraldehyde for 24 h. The samples were rinsed with PBS, post-fixed with 1% osmium tetroxide for 1 h, and rinsed again with PBS. Subsequently, the cells were dehydrated through a graded EtOH series and embedded in EPON resin. Ultrathin sections were prepared, stained with 1% uranyl acetate and lead citrate, and imaged using a TEM (HT7800).

### Animals and tumor models

All animal procedures were approved by the Animal Ethics Committee of Northwestern Polytechnical University (202201035) and conducted in accordance with the *Guide for the Care and Use of Laboratory Animals* (8th edition, 2011). Female BALB/c nude mice (5 weeks, 18 to 22 g) were purchased from Beijing Weitong Lihua Experimental Animal Technology Co. Ltd. (Beijing, China). Subcutaneous tumors were generated by subcutaneous injection of A549 cells (containing 5 × 10^6^ cells in 100 μl of PBS) into the right flank of recipient mice. After the tumor volume reached about 100 mm^3^, the mice were utilized for in vivo fluorescence imaging and phototherapy. Tumor volume (*V*) = (length × width^2^)/2.

### In vivo fluorescence imaging of tumors

A549 tumor-bearing BALB/c nude mice (*n* = 3) were intravenously injected with McL3@HSA (200 μM, 80 μl) via the tail vein. Fluorescence signals were monitored in vivo at specified intervals (0 to 72 h). At 24 h post-injection, tumors and organs were harvested for ex vivo imaging using an IVIS Spectrum imaging system (excitation/emission: 480/620 nm).

### In vivo phototherapy of tumors

A549 tumor-bearing BALB/c nude mice (tumor volume ~100 mm^3^) were randomized into 4 groups (*n* = 5): PBS, PBS + light (L), McL3@HSA, and McL3@HSA + L. Mice received intravenous injections of PBS (80 μl) and McL3@HSA (200 μM, 80 μl) every 2 d. Following injection, at the 8-h time point, the tumor region of mice in the designated light groups was irradiated with white light (420-nm long-pass filter, 80 mW cm^−2^, 10 min) while shielding the rest of the body. Tumor volumes and body weights were recorded every 2 d throughout the treatment period.

### H&E and TUNEL staining

Tumors and organs (heart, liver, spleen, lungs, and kidneys) were harvested from euthanized mice. After fixation in 4% paraformaldehyde, tissue samples were dehydrated, followed by paraffin embedding and sectioning at 5-μm thickness. For histopathological examination, sections were stained with H&E. Apoptosis was measured using the TUNEL assay according to the manufacturer’s protocol.

### Statistical analysis

All data were analyzed using GraphPad Prism software and are presented as the mean ± standard deviation (SD). For comparisons among multiple groups, one-way analysis of variance (ANOVA) followed by Tukey’s post hoc test was performed. A difference was considered statistically significant where probability (*P*) values < 0.05.

## Data Availability

All data are available in the main text or the Supplementary Materials.

## References

[1] Li X, Lovell JF, Yoon J, Chen X. Clinical development and potential of photothermal and photodynamic therapies for cancer. Nat Rev Clin Oncol. 2020;17(11):657–674. 32699309 10.1038/s41571-020-0410-2

[2] Xie J, Wang Y, Choi W, Jangili P, Ge Y, Xu Y, Kang J, Liu L, Zhang B, Xie Z, et al. Overcoming barriers in photodynamic therapy harnessing nano-formulation strategies. Chem Soc Rev. 2021;50(16):9152–9201. 34223847 10.1039/d0cs01370f

[3] Zhang T, Qu X, Shao J, Dong X. Organic photosensitizers: From molecular design to phototheranostics. Chem Soc Rev. 2025;54(18):8406–8433. 40856124 10.1039/d5cs00599j

[4] Yang J, Griffin A, Qiang Z, Ren J. Organelle-targeted therapies: A comprehensive review on system design for enabling precision oncology. Signal Transduct Target Ther. 2022;7(1):379. 36402753 10.1038/s41392-022-01243-0 PMC9675787

[5] Li M, Xiong J, Zhang Y, Yu L, Yue L, Yoon C, Kim Y, Zhou Y, Chen X, Xu Y, et al. New guidelines and definitions for type I photodynamic therapy. Chem Soc Rev. 2025;54(15):725–757. 10.1039/d1cs01079d 40539837

[6] Zhang Y, Feng G, He T, Yang M, Lin J, Huang P. Traceable lactate-fueled self-acting photodynamic therapy against triple-negative breast cancer. Research. 2024;7:277. 10.34133/research.0277 PMC12326368 40771576

[7] Zhu J, Peng L, Jehan S, Wang H, Chen X, Zhao S, Zhou W. Activable photodynamic DNA probe with an “and” logic gate for precision skin cancer therapy. Research. 2024;7:295. 10.34133/research.0295 PMC10807844 38269029

[8] Teng KX, Chen WK, Niu LY, Fang WH, Cui G, Yang QZ. BODIPY-based photodynamic agents for exclusively generating superoxide radical over singlet oxygen. Angew Chem Int Ed Engl. 2021;60(36):19912–19920. 34227724 10.1002/anie.202106748

[9] Zhao C, Du T, Zhu B, He Z, Wang H, Liu Y. Activatable near-infrared organic photosensitizers for imaging-guided photodynamic therapy. Coord Chem Rev. 2025;544: Article 216962.

[10] Ding Q, Zhou L, Xiong T, Liu J, Chen L, Yoo J, Xu X, Jia X, Chen S, Chen S, et al. Sunlight PDT leveraging NIR-II nanospray: Painless, hemostatic, anti-inflammatory therapy towards diabetic wound infections. Natl Sci Rev. 2026;13(2):nwaf554. 41608042 10.1093/nsr/nwaf554 PMC12839536

[11] Zhuang J, Qi G, Feng Y, Wu M, Zhang H, Wang D, Zhang X, Chong KC, Li B, Liu S, et al. Thymoquinone as an electron transfer mediator to convert type II photosensitizers to type I photosensitizers. Nat Commun. 2024;15(1):4943. 38858372 10.1038/s41467-024-49311-z PMC11164902

[12] Huang L, Zhao S, Wu J, Yu L, Singh N, Yang K, Lan M, Wang P, Kim JS. Photodynamic therapy for hypoxic tumors: Advances and perspectives. Coord Chem Rev. 2021;438: Article 213888.

[13] Ding Q, Kim JS. Molecularly intelligent photosensitizers: Pioneering a new era in photodynamic therapy. Sci China Chem. 2026;69(1):18–24.

[14] Wan Y, Fu LH, Li C, Lin J, Huang P. Conquering the hypoxia limitation for photodynamic therapy. Adv Mater. 2021;33(48):2103978. 10.1002/adma.202103978 34580926

[15] Chen H, Wang Y, He Z, Wan Y, Cao C, Lu Z, Gao Y, Cui X, Lee KW, Tan JH, et al. De novo design of efficient NIR-II-activated heavy-atom-free type-I photosensitizer for anti-tumor photoimmunotherapy. Adv Mater. 2025;37(33):2501919. 10.1002/adma.202501919 40465313

[16] Ryu KW, Fung TS, Baker DC, Saoi M, Park J, Febres-Aldana CA, Aly RG, Cui R, Sharma A, Fu Y, et al. Cellular ATP demand creates metabolically distinct subpopulations of mitochondria. Nature. 2024;635(8039):746–754. 39506109 10.1038/s41586-024-08146-w PMC11869630

[17] Guo X, Yang N, Ji W, Zhang H, Dong X, Zhou Z, Li L, Shen HM, Yao SQ, Huang W. Mito-bomb: Targeting mitochondria for cancer therapy. Adv Mater. 2021;33(43):2007778. 10.1002/adma.202007778 34510563

[18] Dirak M, Yenici CM, Kolemen S. Recent advances in organelle-targeted organic photosensitizers for efficient photodynamic therapy. Coord Chem Rev. 2024;506: Article 215710.

[19] Xu S, Yang N, Du F, Zhang Z, Zhang Y, Zhang Y, Liang J, Zhao Y, Zhang J, Zhang Z, et al. Mitochondrial-targeted photodynamic therapy combined with TGF-β inhibition potentiates anti-PD-1 therapy in pancreatic ductal adenocarcinoma. J Nanobiotechnol. 2025;23(1):748. 10.1186/s12951-025-03795-z PMC12661700 41310702

[20] Fang B, Bai H, Zhang J, Wang L, Li P, Ge Y, Yang H, Wang H, Peng B, Hu W, et al. Albumins constrainting the conformation of mitochondria-targeted photosensitizers for tumor-specific photodynamic therapy. Biomaterials. 2025;315: Article 122914. 39461059 10.1016/j.biomaterials.2024.122914

[21] Zhou X, Zhu X, Wang W, Wang J, Wen H, Zhao Y, Zhang J, Xu Q, Zhao Z, Ni T. Comprehensive cellular senescence evaluation to aid targeted therapies. Research. 2025;8:576. 10.34133/research.0576 PMC11735710 39822281

[22] Ding Q, Wang C, Wang H, Xiang C, Wang Z, Wang Y, Zhao L, Vendrell M, Kim JS. Rabies virus targeting NIR-II phototheranostics. J Am Chem Soc. 2025;147(19):16661–16673. 40315345 10.1021/jacs.5c04975 PMC12082629

[23] Dai H, Pan J, Shao J, Xu K, Ruan X, Mei A, Chen P, Qu L, Dong X. Boosting nonradiative decay of boron difluoride formazanate dendrimers for NIR-II photothermal theranostics. Angew Chem Int Ed Engl. 2025;64(21): Article e202503718. 40071493 10.1002/anie.202503718

[24] Belyaev A, Cheng YH, Liu ZY, Karttunen AJ, Chou PT, Koshevoy IO. A facile molecular machine: Optically triggered counterion migration by charge transfer of linear donor-π-acceptor phosphonium fluorophores. Angew Chem Int Ed Engl. 2019;131(38):13590–13599. 10.1002/anie.201906929 31291049

[25] Han C, Kundu BK, Chen R, Pragti SP, Elles CG, Sun Y. Near-infrared light-driven condensation using branched two-photon-absorbing organic photocatalysts with viscosity-dependent properties. J Am Chem Soc. 2025;147(24):20525–20533. 40490684 10.1021/jacs.5c02797

[26] Li C, Li J, Pang Y, Mei L, Xu W, Zhang Z, Han C, Sun Y. Harnessing donor cyclization strategy: Converting type II to type I photosensitizers and enhancing AIE performance for NIR-II FL/MR imaging-guided photodynamic therapy under hypoxia condition. Chem Eng J. 2024;498: Article 155471.

[27] Dai Y, Xu J, Lei X, Meng QY, Qiao J. Molecular engineering of donor/π-bridge enables high-efficiency and long-lifetime near-infrared TADF-OLEDs. Adv Funct Mater. 2025;35(2):2412780.

[28] Li X, Li X, Park S, Wu S, Guo Y, Nam KT, Kwon N, Yoon J, Hu Q. Photodynamic and photothermal therapy via human serum albumin delivery. Coord Chem Rev. 2024;520: Article 216142.

[29] Fang B, Bai H, Zhang J, Shi M, Ge Y, Wang L, Li P, Ding Y, Zhang S, Zhang C, et al. Fluorogen-activating human serum albumin for mitochondrial nanoscale imaging. Adv Mater. 2025;37(35):2501849. 10.1002/adma.202501849 40420673

[30] Huang J, Liu J, Wu J, Xu M, Lin Y, Pu K. Near-infrared chemiluminophore switches photodynamic processes via protein complexation for biomarker-activatable cancer therapy. Angew Chem Int Ed Engl. 2025;64(12): Article e202421962. 39587712 10.1002/anie.202421962

[31] Wang T, Ma L, Bu Y, Xu X, Zhao X, Ni Y, Zhao Q, Lu Z, Ge F, Zhou H. Versatile albumin as a booster to optimize the excited state energy redistribution in photosensitizers enabling fluorescence imaging-guided photodynamic therapy. Chem Eng J. 2025;515: Article 163896.

[32] Zhang Z, Wang Q, Zhang X, Mei D, Mei J. Modulating the luminescence, photosensitizing properties, and mitochondria-targeting ability of D-π-A-structured dihydrodibenzo[a,c]phenazines. Molecules. 2023;28(17):6392. 37687220 10.3390/molecules28176392 PMC10490149

[33] Chen G, Sun J, Peng Q, Sun Q, Wang G, Cai Y, Gu X, Shuai Z, Tang BZ. Biradical-featured stable organic-small-molecule photothermal materials for highly efficient solar-driven water evaporation. Adv Mater. 2020;32(29):1908537. 10.1002/adma.201908537 32519356

[34] Fang L, Meng Q, Zhang Y, Su R, Xing F, Yang H, Hou Y, Ma PA, Huang K, Feng S. π bridge engineering-boosted dual enhancement of type-I photodynamic and photothermal performance for mitochondria-targeting multimodal phototheranostics of tumor. ACS Nano. 2023;17(21):21553–21566. 37910516 10.1021/acsnano.3c06542

[35] Wang C, Chi W, Qiao Q, Tan D, Xu Z, Liu X. Twisted intramolecular charge transfer (TICT) and twists beyond TICT: From mechanisms to rational designs of bright and sensitive fluorophores. Chem Soc Rev. 2021;50(22):12656–12678. 34633008 10.1039/d1cs00239b

[36] Zeng S, Chen C, Guo Z, Qin C, Wang Y, Liu X, Li X, Jeong H, Hao Y, Zhou D, et al. A photon-driven unimolecular immunostimulant for self-amplified pyroptosis and cGAS-STING pathway by destroying the pyroptosis checkpoint. Angew Chem Int Ed Engl. 2025;64(41): Article e202513815. 40844200 10.1002/anie.202513815 PMC12501661

[37] Ding Q, Ding L, Xiang C, Li C, Kim E, Yoon C, Wang H, Gu M, Zhang P, Wang L, et al. pH-responsive AIE photosensitizers for enhanced antibacterial therapy. Angew Chem Int Ed Engl. 2025;64(27): Article e202506505. 40299633 10.1002/anie.202506505 PMC12207372

[38] Liu J, Ou X, Wang K, Wang K, Gui L, Song F, Chen C, Lam JWY, Yuan Z, Tang BZ. Two-photon-activated heavy-atom free AIEgen for highly efficient type I photodynamic therapy. Adv Funct Mater. 2024;34(51):2410202.

[39] Chen W, Wang Z, Tian M, Hong G, Wu Y, Sui M, Chen M, An J, Song F, Peng X. Integration of TADF photosensitizer as “electron pump” and BSA as “electron reservoir” for boosting type I photodynamic therapy. J Am Chem Soc. 2023;145(14):8130–8140. 37001012 10.1021/jacs.3c01042

[40] Zeng S, Wang Y, Chen C, Kim H, Liu X, Jiang M, Yu Y, Kafuti YS, Chen Q, Wang J, et al. An ER-targeted, viscosity-sensitive hemicyanine dye for the diagnosis of nonalcoholic fatty liver and photodynamic cancer therapy by activating pyroptosis pathway. Angew Chem Int Ed Engl. 2024;63(9): Article e202316487. 38197735 10.1002/anie.202316487

[41] Fang B, Li P, Jiang J, Du W, Wang L, Bai H, Peng B, Huang X, An Z, Li L, et al. Confinement fluorescence effect (CFE): Lighting up life by enhancing the absorbed photon energy utilization efficiency of fluorophores. Coord Chem Rev. 2021;440: Article 213979.

[42] Golombek SK, May J, Theek B, Appold L, Drude N, Kiessling F, Lammers T. Tumor targeting *via* EPR: Strategies to enhance patient responses. Adv Drug Deliv Rev. 2018;130:17–38. 30009886 10.1016/j.addr.2018.07.007 PMC6130746

[43] Qin S, Cheng X, Zhou Z, Zhang X, Chen J, Xu P, Wu T, Hu Y. Protein-confined rotor strategy for quantum yield enhancement in supramolecular photosensitizers toward sentinel lymph node-targeted photodynamic immunoactivation. ACS Nano. 2025;19(27):24985–25006. 40590707 10.1021/acsnano.5c04279

[44] Li X, Yu S, Lee Y, Guo T, Kwon N, Lee D, Yeom SC, Cho Y, Kim G, Huang J, et al. In vivo albumin traps photosensitizer monomers from self-assembled phthalocyanine nanovesicles: A facile and switchable theranostic approach. J Am Chem Soc. 2019;141(3):1366–1372. 30565924 10.1021/jacs.8b12167

[45] Han F, Guo M, Zhou X, Zhang Z, Zhang H, Cai L, Li X, Shi T, Long S, Sun W, et al. Precise molecular engineering of heptamethine cyanine-based near-infrared type-I photosensitizers for pro-death autophagy and hypoxia-tolerant antitumor treatment. Angew Chem Int Ed Engl. 2025;64(34): Article e202504227. 40350761 10.1002/anie.202504227

[46] Zhou Y, Ma L, Lunchev AV, Long S, Wu T, Ni W, Grimsdale AC, Sun L, Gurzadyan GG. Switching pathways of triplet state formation by twisted intramolecular charge transfer. J Phys Chem B. 2021;125(45):12518–12527. 34752093 10.1021/acs.jpcb.1c07045

[47] Gu H, Yuan P, Zhang J, Xia X, Pan Q, Liu W, Zhao X, Sun W, Du J, Fan J, et al. Tuning exciton coupling of non-conjugated cyanine dimers for efficient photodynamic immunotherapy. J Am Chem Soc. 2025;147(24):20778–20789. 40471127 10.1021/jacs.5c04044

[48] Hu W, He T, Zhao H, Tao H, Chen R, Jin L, Li J, Fan Q, Huang W, Baev A, et al. Stimuli-responsive reversible switching of intersystem crossing in pure organic material for smart photodynamic therapy. Angew Chem Int Ed Engl. 2019;58(32):11105–11111. 31172619 10.1002/anie.201905129

[49] Jiang Y, Huang S, Ma H, Weng J, Du X, Lin Z, Kim J, You W, Zhang H, Wang D, et al. RNA-activatable near-infrared photosensitizer for cancer therapy. J Am Chem Soc. 2024;146(36):25270–25281. 39215718 10.1021/jacs.4c09470

[50] Tam LKB, Chu JCH, He L, Yang C, Han K, Cheung PCK, Ng DKP, Lo P. Enzyme-responsive double-locked photodynamic molecular beacon for targeted photodynamic anticancer therapy. J Am Chem Soc. 2023;145(13):7361–7375. 36961946 10.1021/jacs.2c13732 PMC10080691

[51] Frisch MJ, Trucks GW, Schlegel HB, Scuseria GE, Robb MA, Cheeseman JR, G. Scalmani, Barone V, Mennucci B, Petersson GA, et al. *Gaussian 16 Rev. C.01*. Wallingford (CT): Gaussian Inc.; 2016.

[52] Neese F. The ORCA program system. WIREs Comput Mol Sci. 2012;2(1):73–78.

[53] Neese F. Efficient and accurate approximations to the molecular spin-orbit coupling operator and their use in molecular g-tensor calculations. J Chem Phys. 2005;122(3):34107. 15740192 10.1063/1.1829047

[54] Raza SA, Naqvi SQ, Usman A, Jennings JR, Soon YW. Spectroscopic study of the interaction between rhodamine B and graphene. J Photochem Photobiol A Chem. 2021;418: Article 113417.

[55] An Z, Zheng C, Tao Y, Chen R, Shi H, Chen T, Wang Z, Li H, Deng R, Liu X, et al. Stabilizing triplet excited states for ultralong organic phosphorescence. Nat Mater. 2015;14(7):685–690. 25849370 10.1038/nmat4259

