# Supplementary material for: “Dual-Boosting” Strategy to Enhance Radical Generation of Photosensitizer for Mitochondria-Targeted Phototherapy
Source: Research (Wash D C). 2026 May 14;9:1279. doi: 10.34133/research.1279 (PMC13172578; doi:10.34133/research.1279)
Supplement: Supplementary 1 — Supplementary Text Figs. S1 to S36 Tables S1 to S7 [file research.1279.f1279.doc]

Supplementary Materials for

**“Dual-boosting” Strategy to Enhance Radical Generation of Photosensitizer for Mitochondria-targeted Phototherapy**

Limin Wang^1^, Dongming Wu^1^, Haolin Zhang^1^, Panpan Li^1^, Hui Liu^1^, Biying Zhang^1^, Jiacong Yan^2^, Yunxiu Li^2^, Bo Peng^1^, Wenbo Hu^1^, Bin Fang^3,4*^, Hua Bai^1*^, and Lin Li^1,3,4*^

^1^State Key Laboratory of Flexible Electronics (LoFE) & Institute of Flexible Electronics (IFE), Northwestern Polytechnical University, Xi’an 710072, China.

^2^Center of Reproductive Medicine, NHC Key Laboratory of Healthy Birth and Birth Defect Prevention in Western China, First People’s Hospital of Yunnan Province, Kunming 650500, China.

^3^State Key Laboratory of Flexible Electronics (LoFE) & Institute of Flexible Electronics (IFE), Xiamen University, Xiamen 361102, China.

^4^Future Display Institute in Xiamen, Xiamen 361005, China.

^*^Address correspondence to: ifebfang@xmu.edu.cn (B.F.); iamhbai@nwpu.edu.cn (H.B.); iamlli@nwpu.edu.cn (L.L.).

# Instruments

NMR spectra were detected by Bruker AVANCE NEO 500 spectrometer (Bruker). Chemical shift (δ) was reported in parts per million (ppm) relative to tetramethylsilane (TMS, δ = 0.0 ppm) or residual solvent signals (DMSO-*d_6_*: δ = 2.50 ppm for ^1^H, δ = 39.52 ppm for ^13^C). Multiplicities are reported as: s (singlet), d (doublet), t (triplet), q (quartet), dd (doublet of doublets), or m (multiplet); coupling constants (*J* value) are reported in Hz. The high-resolution mass spectra (ESI-HRMS) was obtained by Waters Xevo G2-XS TOF mass spectrometer (Waters). The UV-*vis* absorption spectra were recorded using a U-3900H spectrophotometer (Hitachi) using a quartz cuvette with a 1 cm path length. Fluorescence spectra were obtained using a F-7100 spectrofluorimeter (Hitachi) equipped with a 450 W Xe lamp. Steady-state fluorescence spectra at low-temperature (77 K) and lifetime decay profile were measured using an FLS1000 spectrophotometer (Edinburgh) equipped with a xenon arc lamp (Xe900), time-correlated single photon counting (TSCPC) and oxford instruments cryostat with a temperature control sample holder. Dynamic light scattering (DLS) was obtained with Zetasizer nano ZS (Malvern). Transmission electron microscopy (TEM) images were obtained by HT7800 (Hitachi). The EPR spectrum was measured on Bruker EMXplus-6/1 ((Bruker). Femtosecond transient absorption (fs-TA) spectroscopy was performed using a commercial femtosecond pump-probe system (Newport Corporation). Confocal fluorescence images were obtained by Nikon Laser Scanning C2 System (Nikon). Cell viability and cytotoxicity assay were measured with Spark Multi-mode Plate Reader (Tecan). Live/dead cell staining fluorescence images were observed using an EVOS FL Auto 2 (Thermo Fisher). Flow cytometry analysis carried out by CytoFLEX (Bruker). *In vivo* imaging was recorded by an IVIS Spectrum imaging system (PerkinElmer).

# Synthesis and Characterization

**Scheme S1.** The synthetic strategies of multi-branched donor/π-bridge pyrido cyanine **(McL1**–**3**).

**Synthesis of na-CHO:** Compound **na-CHO** was prepared according to similar procedures with previous work [1]. Compound **na-CHO** (0.65 g, yield: 95%) was obtained as a yellow solid. ^1^H NMR (500 MHz, CDCl_3_), δ ppm: ^1^H NMR (500 MHz, CDCl_3_), δ ppm: 9.95 (s, 1H), 8.29 (s, 1H), 7.91 (d, *J* = 9.2 Hz, 1H), 7.70 (s, 2H), 7.31 (dd, *J* = 9.2, 2.6 Hz, 1H), 6.97 (s, 1H), 4.75 (t, *J* = 5.3 Hz, 1H), 3.62 (dd, *J* = 10.9, 5.4 Hz, 2H), 3.58 (t, *J* = 5.4 Hz, 2H), 3.10 (s, 3H). LC-MS (ESI): calcd. for [C_14_H_15_NO_2_] m/z 229.11, found [M+H]^+^ 230.21.

**Synthesis of py-1:** Compound **py-1** was prepared according to similar procedures with previous work [1]. Compound **py-1** (4.23 g, yield: 90%) was obtained as a white crystalline. ^1^H NMR (500 MHz, DMSO-*d_6_*) δ ppm: 8.97 (d, *J* = 6.1 Hz, 1H), 8.48 (dd, *J* = 11.3, 4.4 Hz, 1H), 8.05 (d, *J* = 7.9 Hz, 1H), 7.95 (t, *J* = 6.9 Hz, 1H), 4.23 (s, 3H), 2.79 (s, 3H). LC-MS (ESI): calcd. for [C_7_H_10_N^+^] m/z 108.08, found [M]^+^ 108.14.

**Synthesis of McL1:** Compound **na-CHO** (0.229 g, 1 mmol) and intermediate **py-1** (0.117 g, 0.5 mmol) were dissolved in EtOH (6 mL) and stirred at 50°C for 5 mins. Under N_2_ atmosphere, piperidine (80 µL) was added dropwise into the above solution, and the reaction was conducted at 75°C for 12 hrs. After the reaction completing (monitored by TLC), cooled to room temperature and the crude product was recrystallized, then purified by flash chromatography (Methanol/DCM = 12/1). **McL1** (0.21 g, yield: 93%) was obtained as a red solid. ^1^H NMR (500 MHz, DMSO-*d_6_*) δ (ppm): 8.86 (d, *J* = 6.2 Hz, 1H), 8.55 (d, *J* = 8.4 Hz, 1H), 8.46 (t, *J* = 7.8 Hz, 1H), 8.06 (d, *J* = 16.2 Hz, 2H), 7.93 (d, *J* = 8.7 Hz, 1H), 7.86 – 7.76 (m, 2H), 7.73 (d, *J* = 8.7 Hz, 1H), 7.54 (d, *J* = 15.8 Hz, 1H), 7.27 (dd, *J* = 9.1, 2.4 Hz, 1H), 6.96 (s, 1H), 4.75 (t, *J* = 5.2 Hz, 1H), 4.38 (s, 3H), 3.70 – 3.50 (m, 4H), 3.10 (s, 3H). ^13^C NMR (125 MHz, DMSO-*d_6_*) δ (ppm): 153.30, 149.25, 146.23, 144.36, 144.23, 136.79, 131.27, 130.21, 128.38, 127.01, 125.61, 124.81, 124.76, 124.62, 116.81, 114.78, 105.25, 58.83, 54.56, 46.39, 40.52. HR-MS (ESI): calcd. for [C_21_H_23_N_2_O^+^] m/z 319.1805, found [M]^+^ 319.1799.

**Synthesis of py-2:** To a Schlenk flask, 2,6-dimethylpyridine (1.07 g, 10.0 mmol) was dissolved in acetone (20 mL) and CH_3_I (2.13 g, 15.0 mmol) was added under N_2_ atmosphere. The reaction mixture was stirred at 55°C for 12 hrs. The deposit was filtered, washed with acetonitrile, and dried *in vacuo*. The white solid compound **py-2** was obtained (2.14 g, yield: 86%). ^1^H NMR (500 MHz, DMSO-*d_6_*) δ (ppm): 8.32 (t, *J* = 7.9 Hz, 1H), 7.88 (d, *J* = 7.9 Hz, 2H), 4.04 (s, 3H), 2.80 (s, 6H). LC-MS (ESI): calcd. for [C_8_H_12_N^+^] m/z 122.10, found [M]^+^ 122.21.

**Synthesis of McL2:** Compound **na-CHO** (0.458 g, 2 mmol) and intermediate **py-2** (0.124 g, 0.5 mmol) were dissolved in EtOH (10 mL) and stirred at 50°C for 5 mins. Under N_2_ atmosphere, piperidine (100 µL) was added dropwise into the above solution, and the reaction was conducted at 75°C for 12 hrs. After the reaction completing (monitored by TLC), cooled to room temperature and the crude product was recrystallized, then purified by flash chromatography (Methanol/DCM = 10/1). **McL2** was obtained as dark red solid (0.30 g, yield: 91 %). ^1^H NMR (500 MHz, DMSO-*d_6_*) δ (ppm): 8.37 (t, *J* = 8.0 Hz, 1H), 8.26 (d, *J* = 8.0 Hz, 2H), 8.06 (s, 2H), 7.94 (d, *J* = 8.6 Hz, 2H), 7.86 (d, *J* = 15.7 Hz, 2H), 7.79 (d, *J* = 9.2 Hz, 2H), 7.72 (d, *J* = 8.8 Hz, 2H), 7.63 (d, *J* = 15.8 Hz, 2H), 7.26 (d, *J* = 9.0 Hz, 2H), 6.96 (s, 2H), 4.76 (t, *J* = 5.1 Hz, 2H), 4.32 (s, 3H), 3.66 – 3.60 (m, 4H), 3.57 (d, *J* = 5.5 Hz, 4H), 3.09 (s, 6H). ^13^C NMR (125 MHz, DMSO-*d_6_*) δ (ppm): 153.38, 145.78, 145.24, 142.09, 136.60, 130.69, 130.12, 128.58, 127.12, 125.73, 124.38, 123.34, 121.00, 116.79, 105.27, 58.82, 54.57, 47.14, 40.60. HR-MS (ESI): calcd. for [C_36_H_38_N_3_O_2_^+^] m/z 544.2959, found [M]^+^ 544.2978.

**Synthesis of py-3:** To a Schlenk flask, 2,4,6-trimethylpyridine (1.21 g, 10.0 mmol) was dissolved in acetone (20 mL), and CH_3_I (2.13 g, 15.0 mmol) was added under N_2_ atmosphere. The reaction mixture was stirred at 55°C for 12 hrs. The deposit was filtered, washed with acetonitrile, and dried *in vacuo*. The white solid compound **py-3** was obtained (2.24 g, yield: 85%). ^1^H NMR (500 MHz, DMSO-*d_6_*) δ (ppm): 7.73 (s, 2H), 3.97 (s, 3H), 2.73 (s, 6H), 2.48 (s, 3H). LC-MS (ESI): calcd. for [C_9_H_14_N^+^] m/z 136.11, found [M]^+^ 136.21.

**Synthesis of McL3:** Compound **na-CHO** (0.687 g, 3 mmol) and **py-3** (0.131 g, 0.5 mmol) were dissolved in 10 mL EtOH and stirred at 50°C for 5 mins. Under N_2_ atmosphere, piperidine (100 µL) was added dropwise into the above solution, and the reaction was conducted at 75°C for 12 hrs. After the reaction completing (monitored by TLC), the mixture cooled to room temperature and the crude product was recrystallized, then purified by flash chromatography (Methanol/DCM = 8/1). **McL3** was obtained as dark purple solid (0.38 g, yield: 86 %). ^1^H NMR (500 MHz, DMSO-*d_6_*) δ (ppm): ^1^H NMR (500 MHz, DMSO) δ 8.29 (dd, *J* = 69.3, 19.7 Hz, 2H), 8.10 – 7.69 (m, 14H), 7.58 (d, *J* = 15.5 Hz, 2H), 7.43 (d, *J* = 15.8 Hz, 2H), 7.27 (d, *J* = 7.0 Hz, 3H), 6.97 (s, 3H), 4.79 (s, 3H), 4.21 (s, 3H), 3.60 (d, *J* = 29.9 Hz, 12H), 3.09 (s, 9H). ^13^C NMR (125 MHz, DMSO-*d_6_*) δ (ppm): 152.70, 152.29, 149.97, 147.94, 141.63, 135.42, 129.42, 128.96, 127.88, 127.81, 127.66, 126.15, 126.08, 125.90, 124.76, 124.65, 123.76, 118.02, 116.04, 115.71, 104.20, 57.74, 53.50, 39.88, 38.21. HR-MS (ESI): calcd. for [C_51_H_53_N_4_O_3_^+^] m/z 769.4113, found [M]^+^ 769.4102.

# Supplementary Figures and Tables

## Supplementary figures


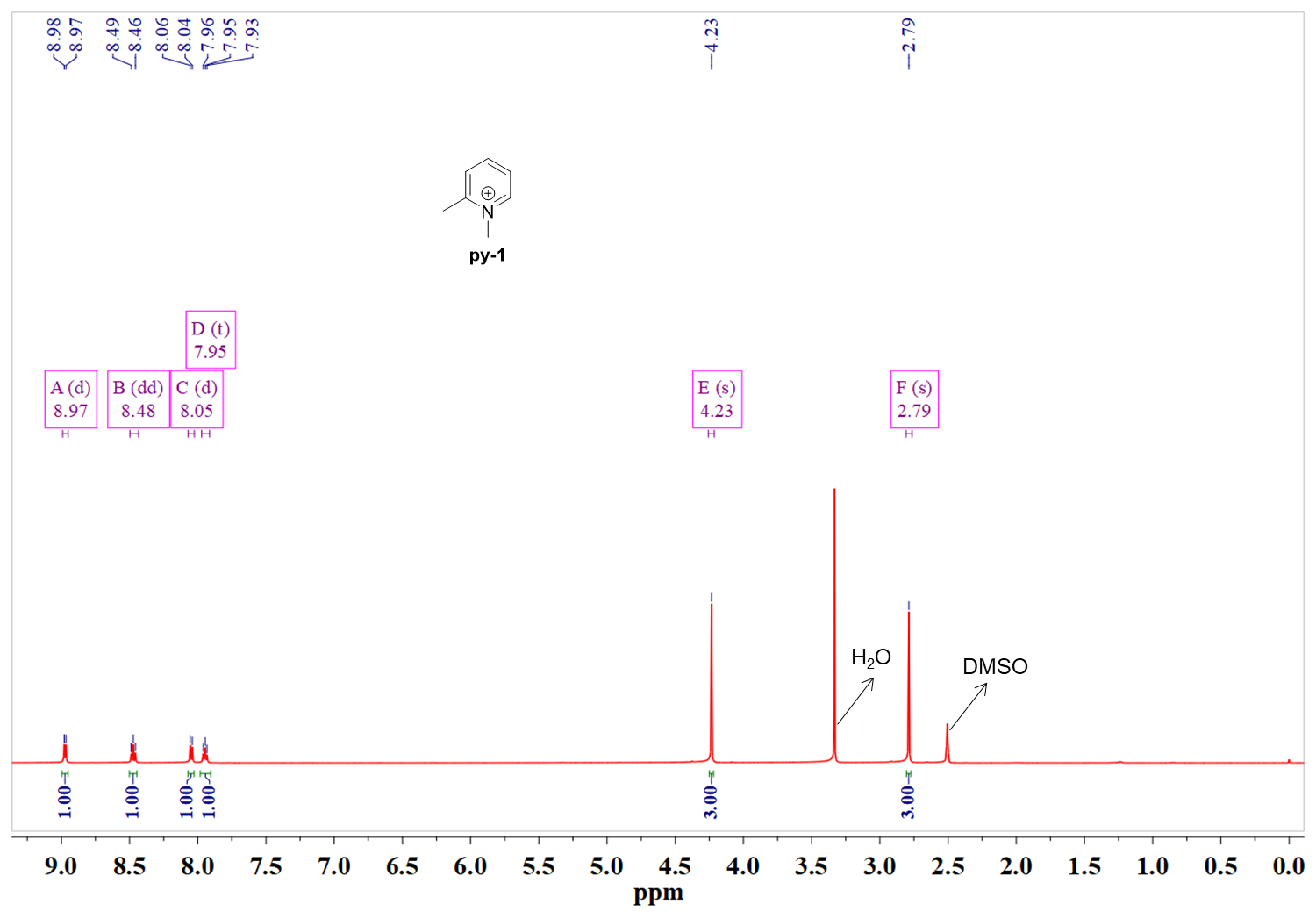


**Figure S1.** ^1^H NMR spectrum of **py-1** (500 MHz, 298 K, DMSO*-d_6_*).


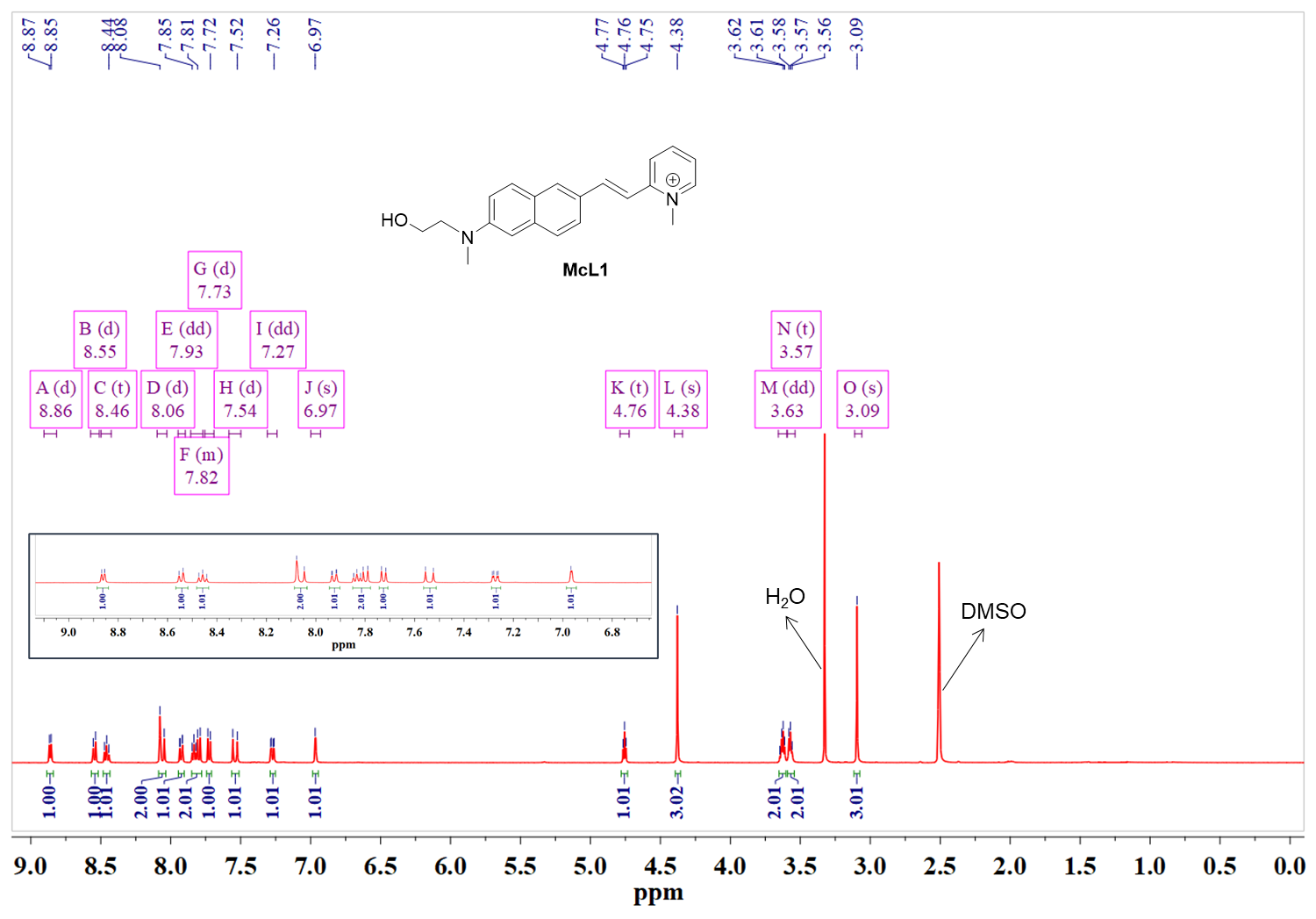


**Figure S2.** ^1^H NMR spectrum of **McL1** (500 MHz, 298 K, DMSO*-d_6_*). The aromatic region is magnified in the insert.


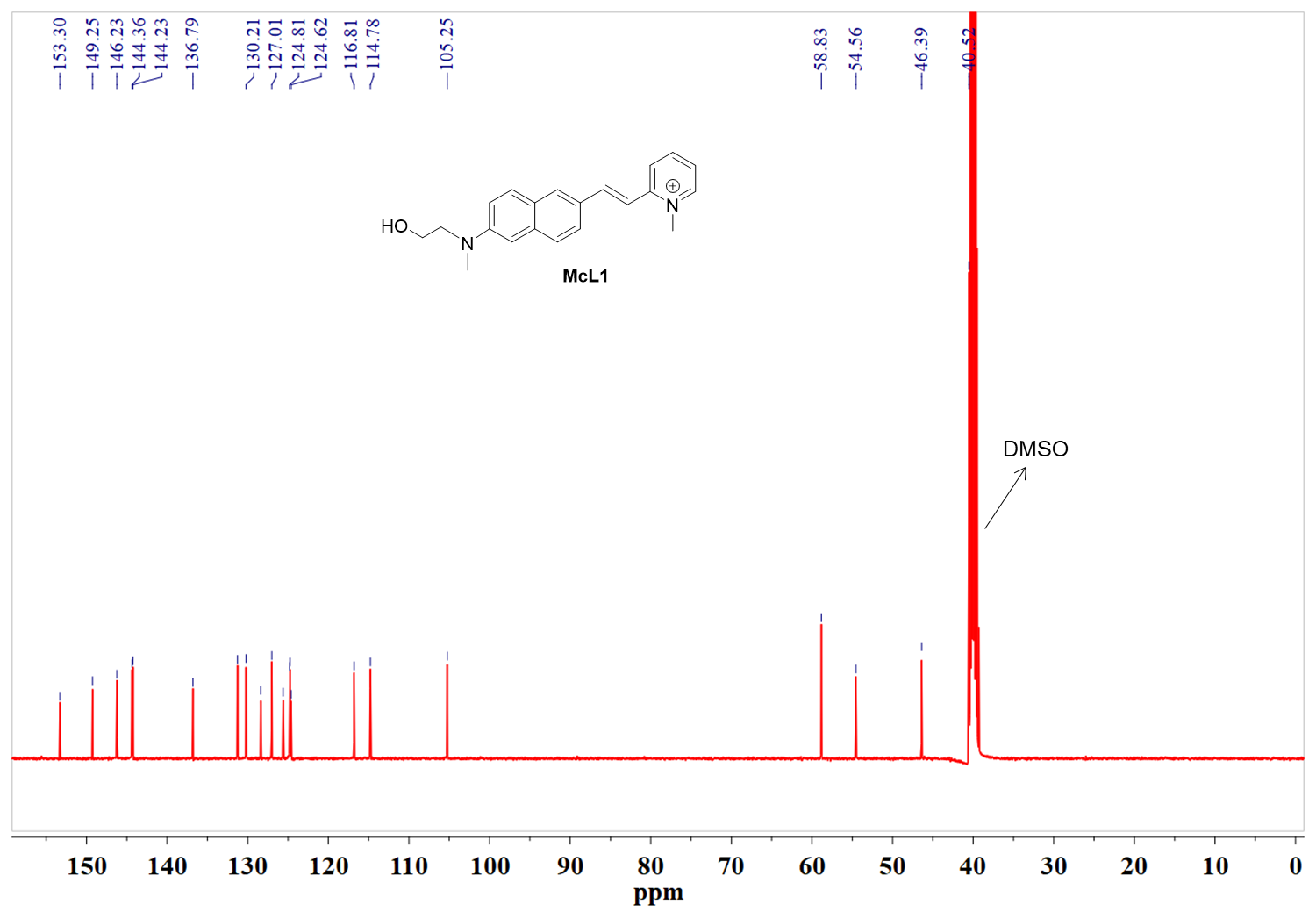


**Figure S3.** ^13^C NMR spectrum of **McL1** (125 MHz, 298 K, DMSO-*d_6_*).


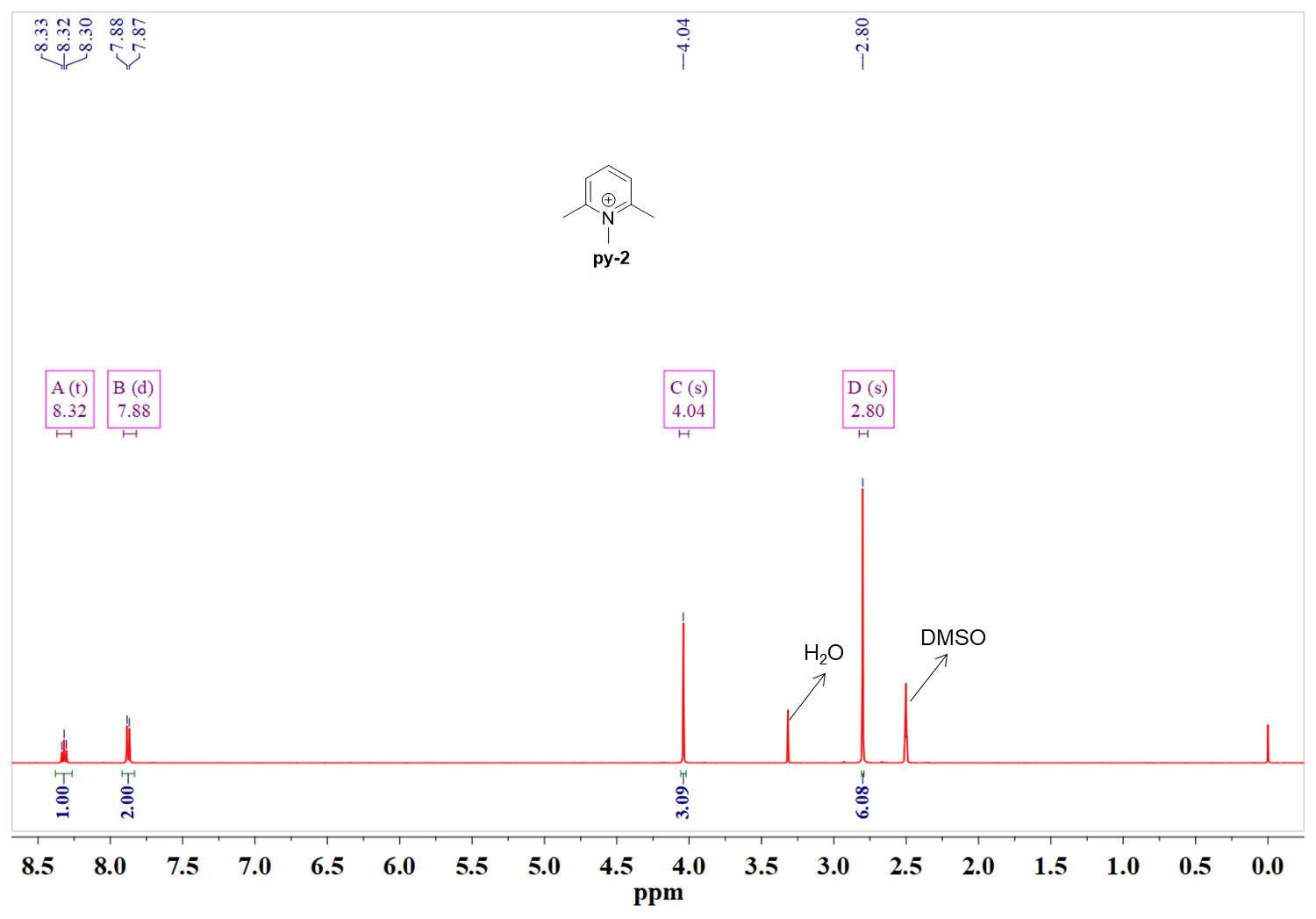


**Figure S4.** ^1^H NMR spectrum of **py-2** (500 MHz, 298 K, DMSO*-d_6_*).


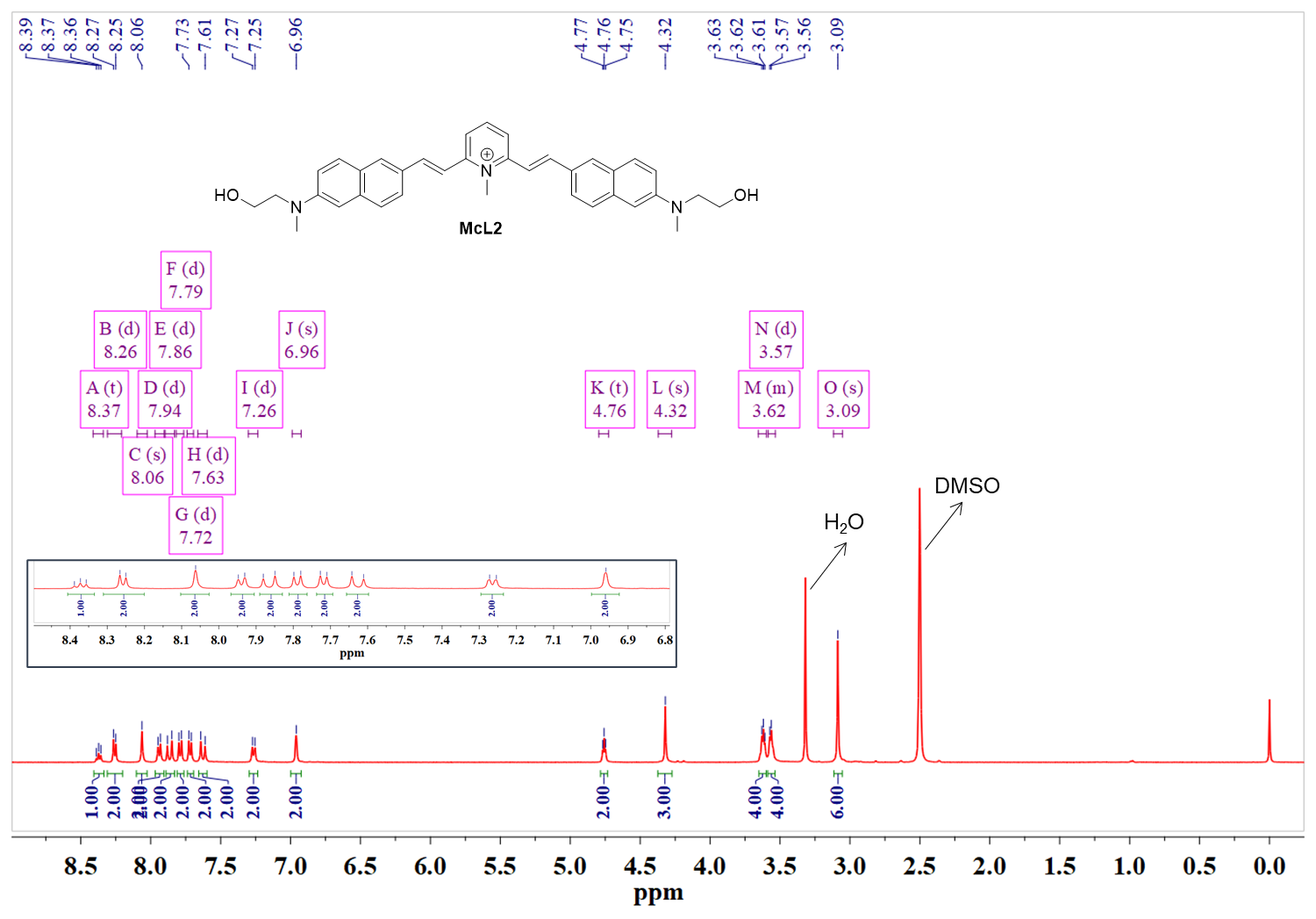


**Figure S5.** ^1^H NMR spectrum of **McL2** (500 MHz, 298 K, DMSO*-d_6_*). The aromatic region is magnified in the insert.


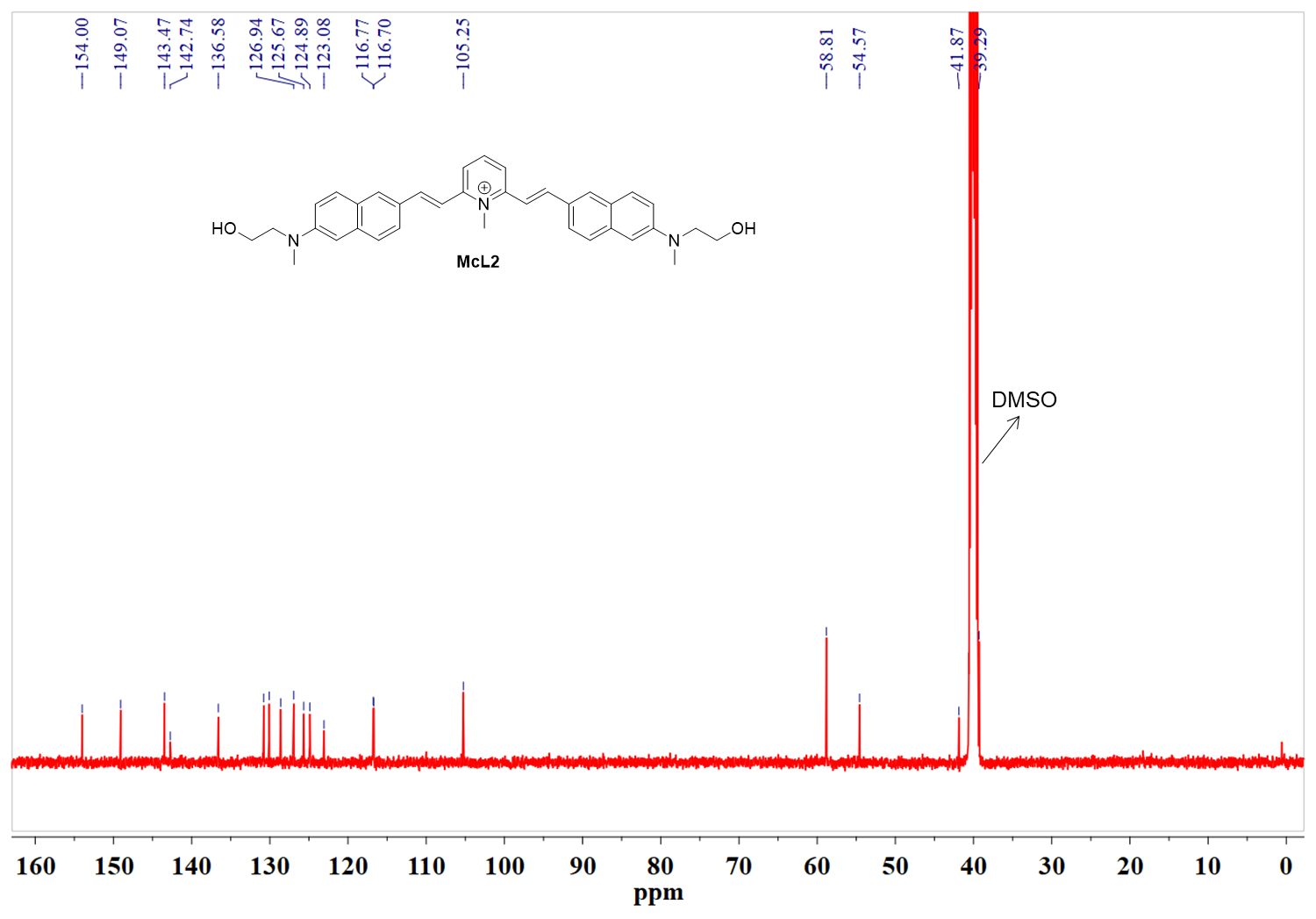


**Figure S6.** ^13^C NMR spectrum of **McL2** (125 MHz, 298 K, DMSO-*d_6_*).


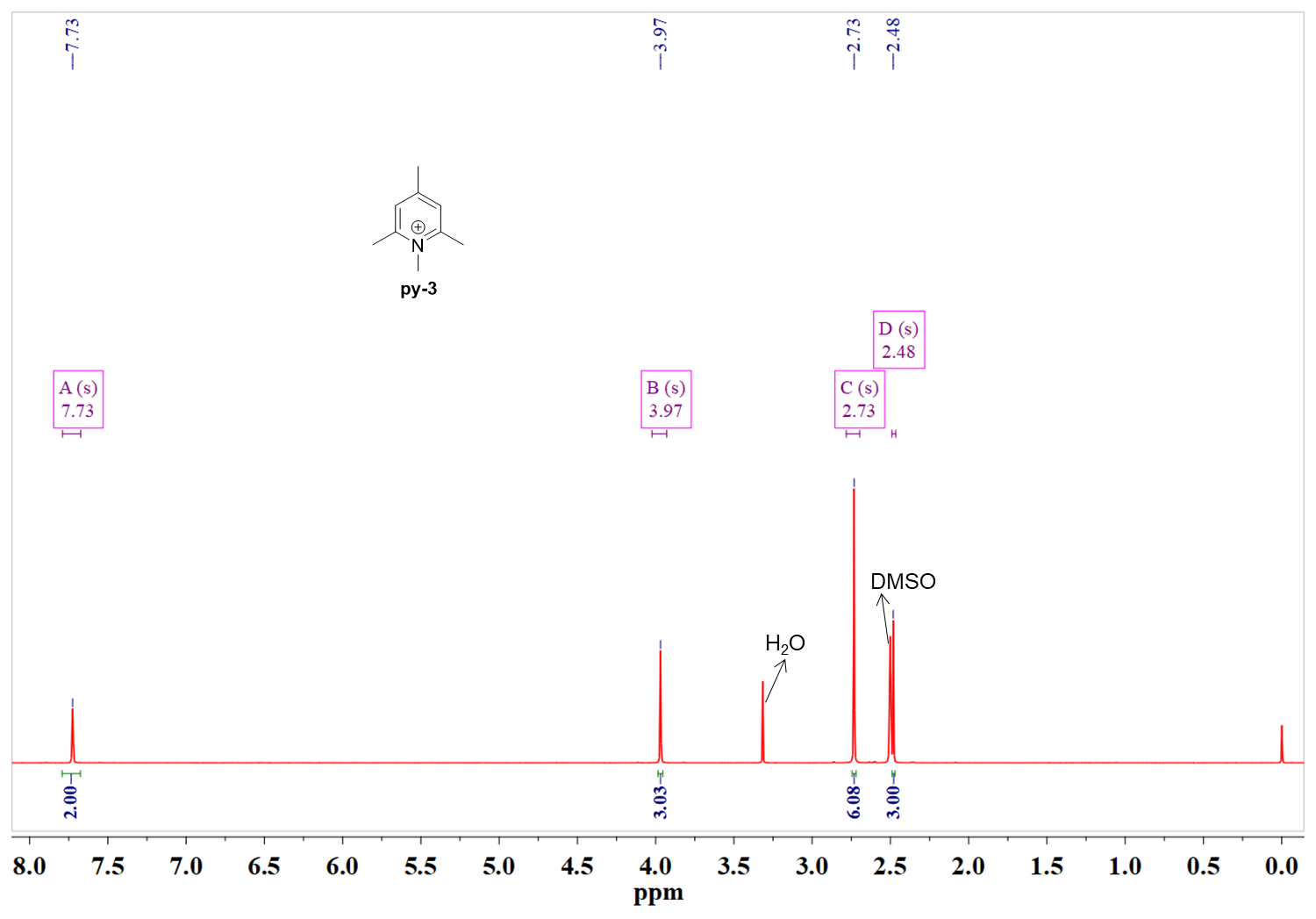


**Figure S7.** ^1^H NMR spectrum of **py-3** (500 MHz, 298 K, DMSO*-d_6_*).


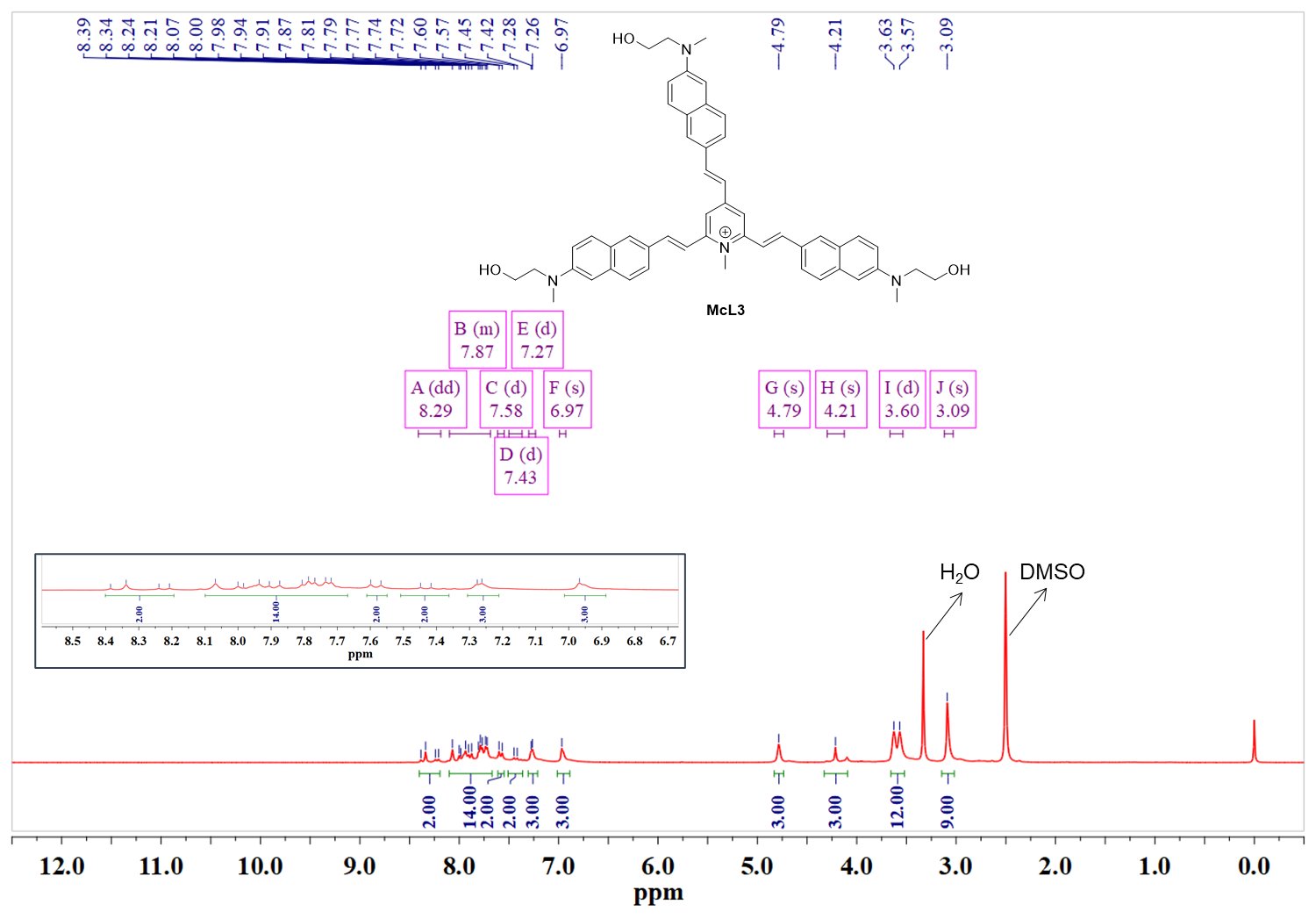


**Figure S8.** ^1^H NMR spectrum of **McL3** (500 MHz, 298 K, DMSO*-d_6_*). The aromatic region is magnified in the insert.


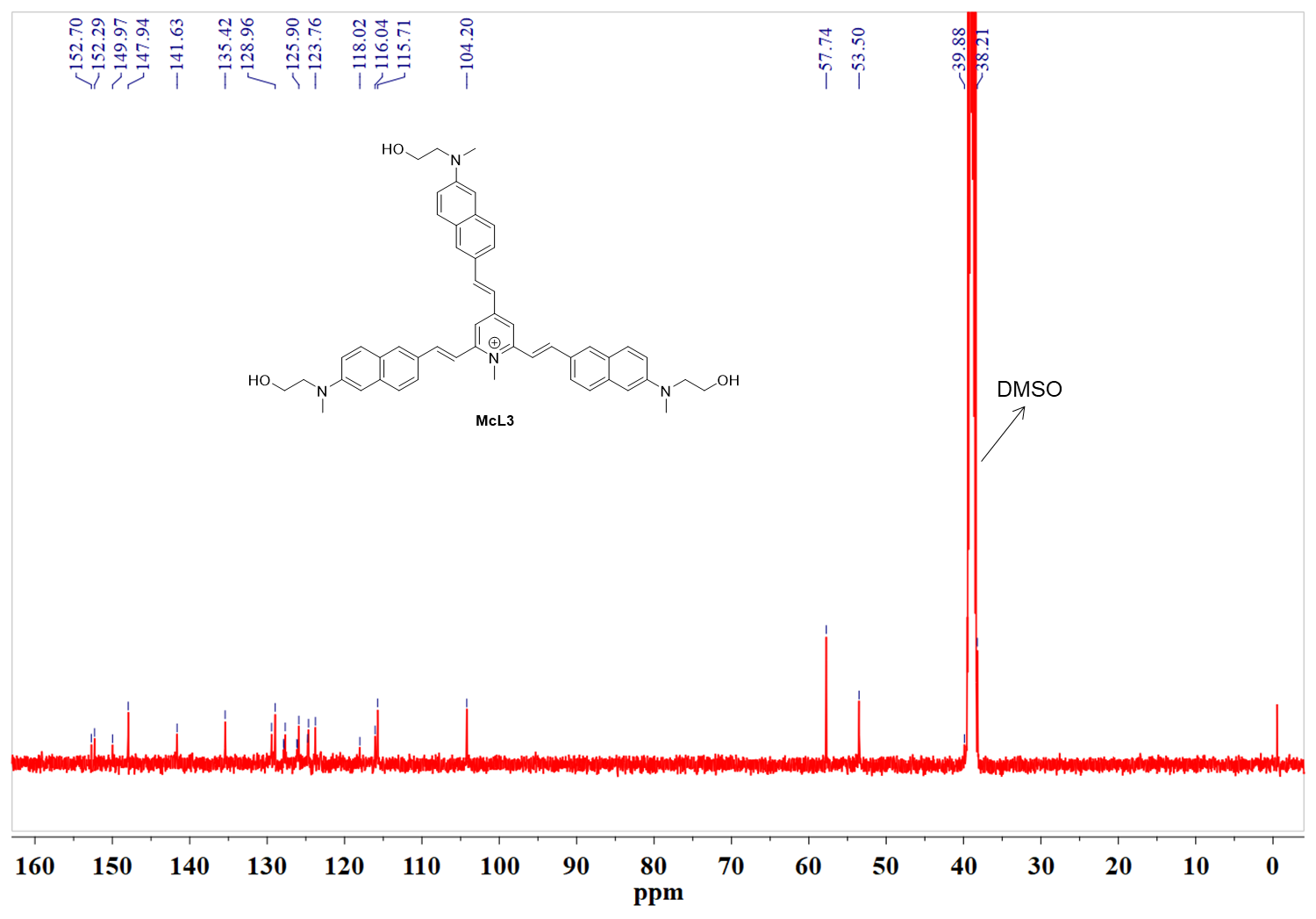


**Figure S9.** ^13^C NMR spectrum of **McL3** (125 MHz, 298 K, DMSO-*d_6_*).


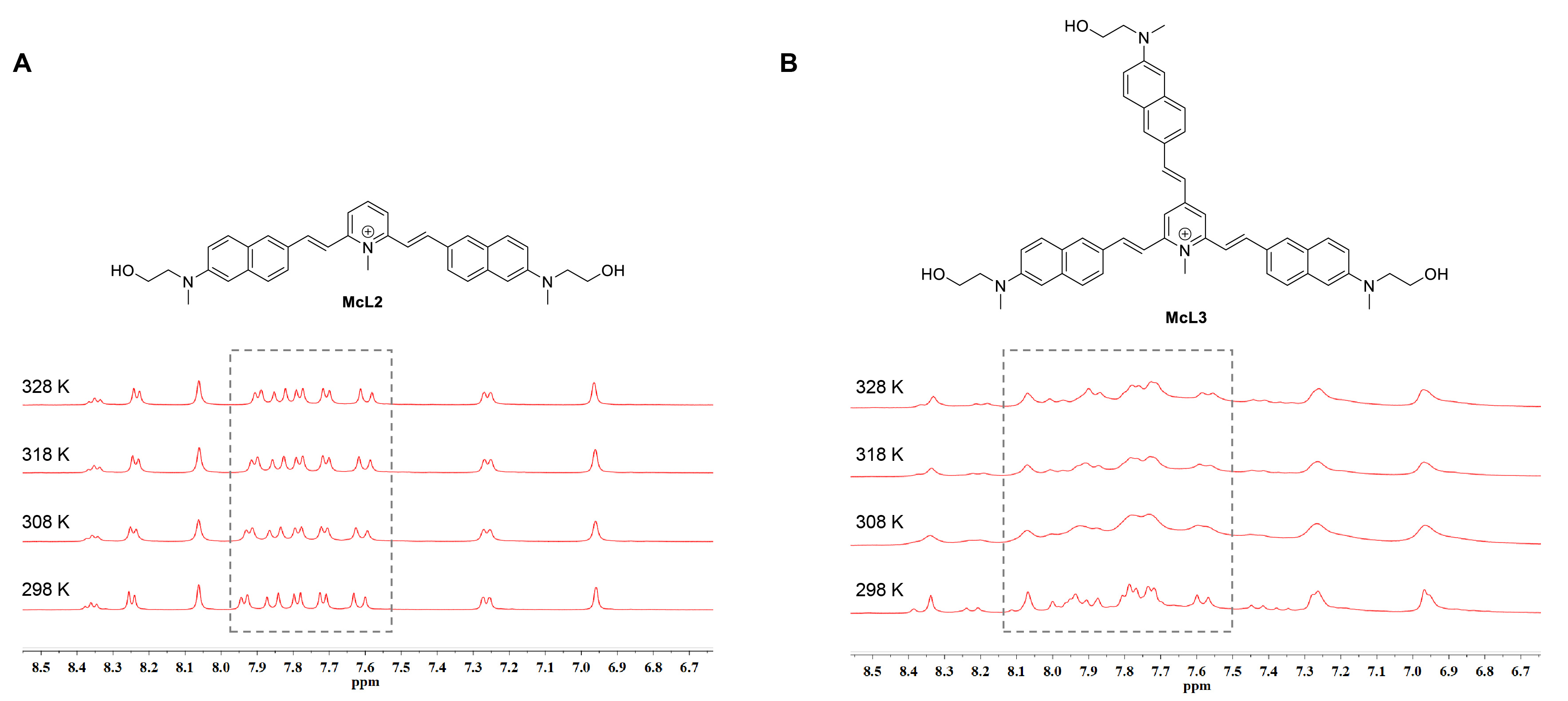


**Figure S10.** Variable-temperature ^1^H NMR (500 MHz, DMSO*-d_6_*) of (A) **McL2** and (B) **McL3**, recorded from 298 K to 328 K.


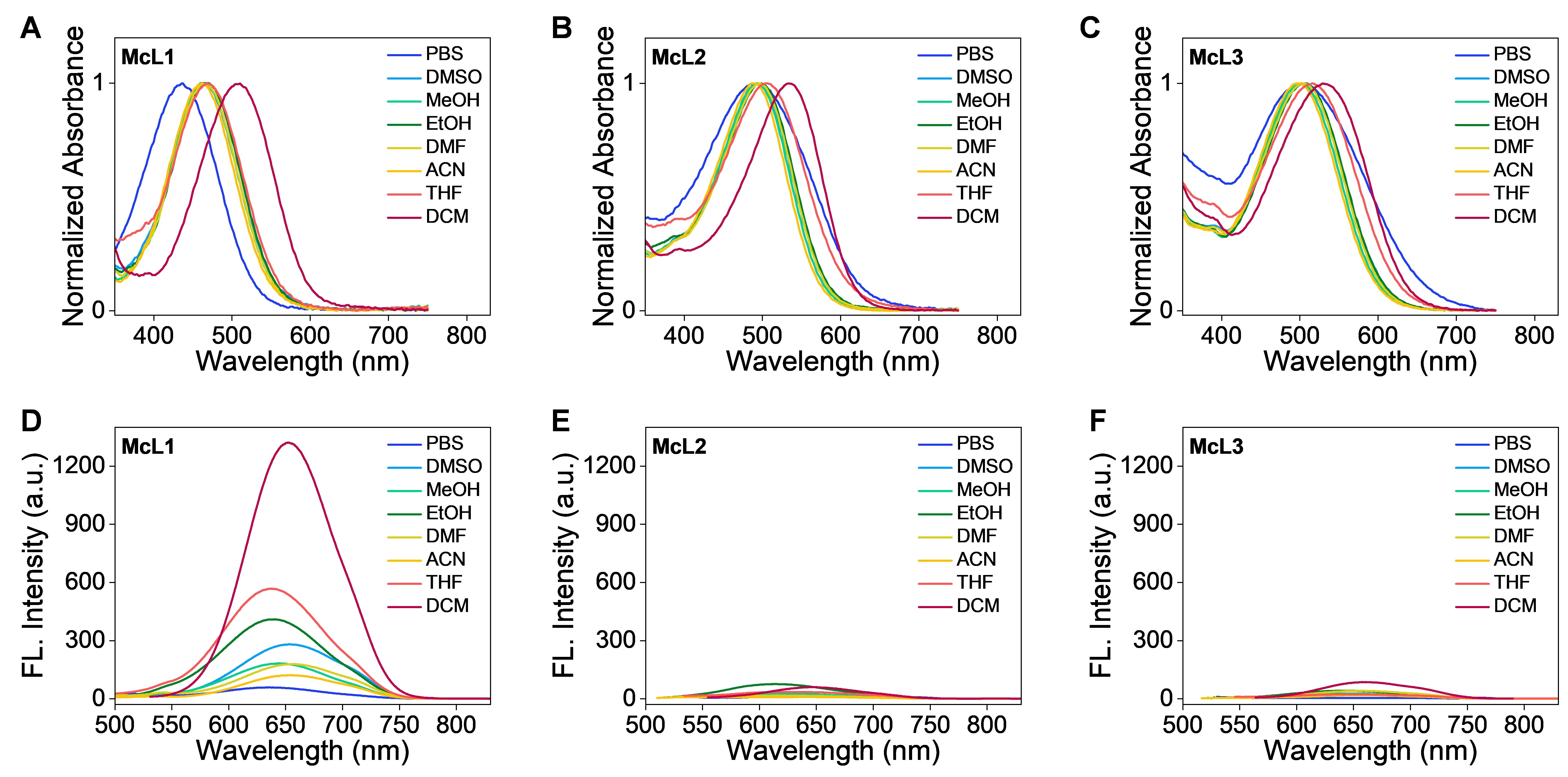


**Figure S11.** (A–C) Normalized UV-*vis* absorption and (D–F) fluorescence emission spectra of **McL1**–**3** (10 μM) in various solvents: PBS (10 mM, pH = 7.42), DMSO, MeOH, EtOH, DMF, ACN, THF, DCM. Emission spectra were recorded upon excitation at the respective absorption maxima (λ_abs_).


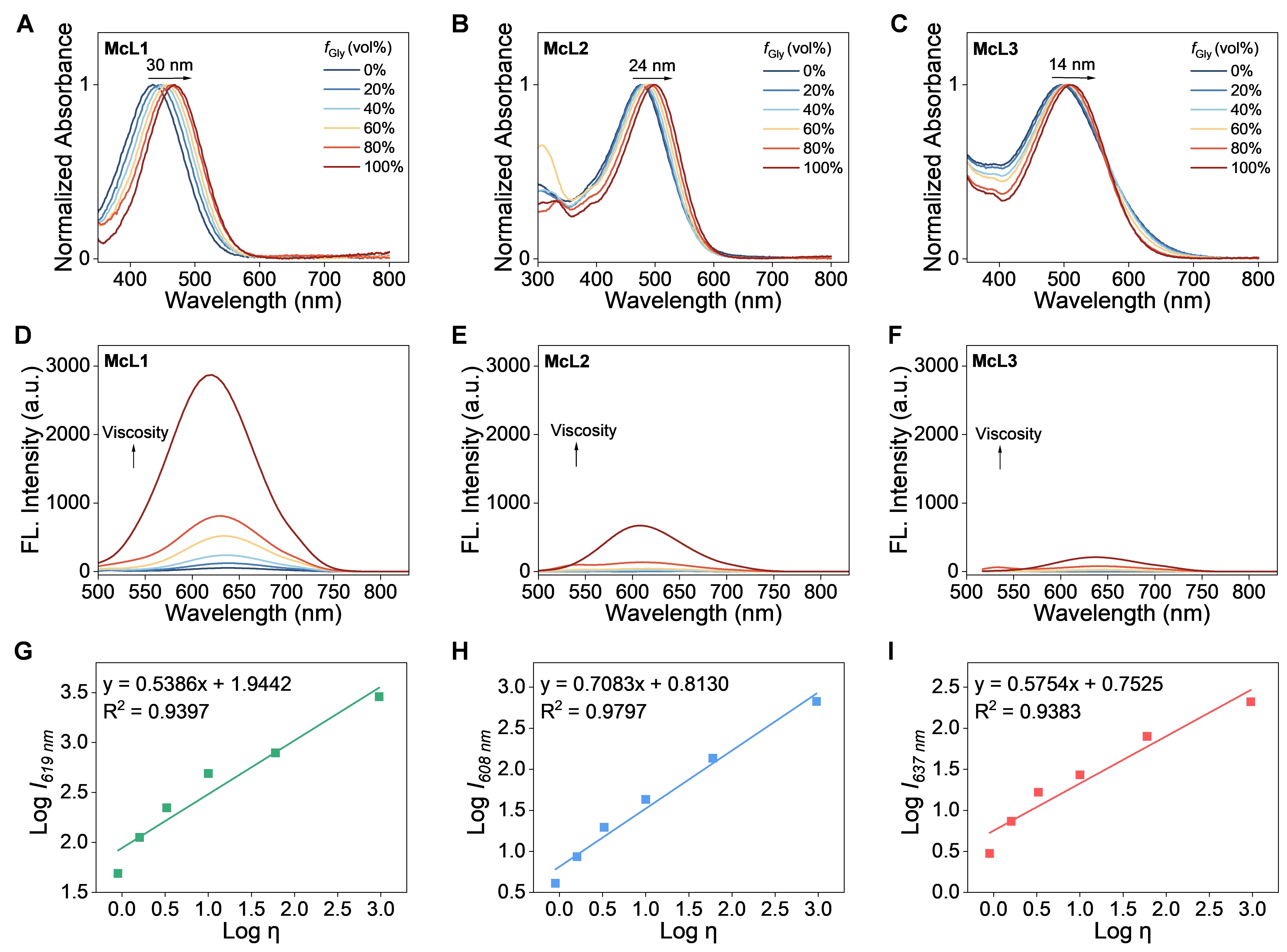


**Figure S12.** (A–C) Normalized UV-*vis* absorption and (D–F) fluorescence emission spectra of **McL1**–**3** (10 μM) in the binary mixture of water-glycerol with different volume ratios (0% to 100% glycerol, corresponding to viscosities (η) of 0.9 cP to 954 cP). (G–I) Linear fit of log(intensity) *vs.* log(viscosity) for **McL1**–**3**. Emission spectra were recorded upon excitation at the respective absorption maxima (λ_abs_).


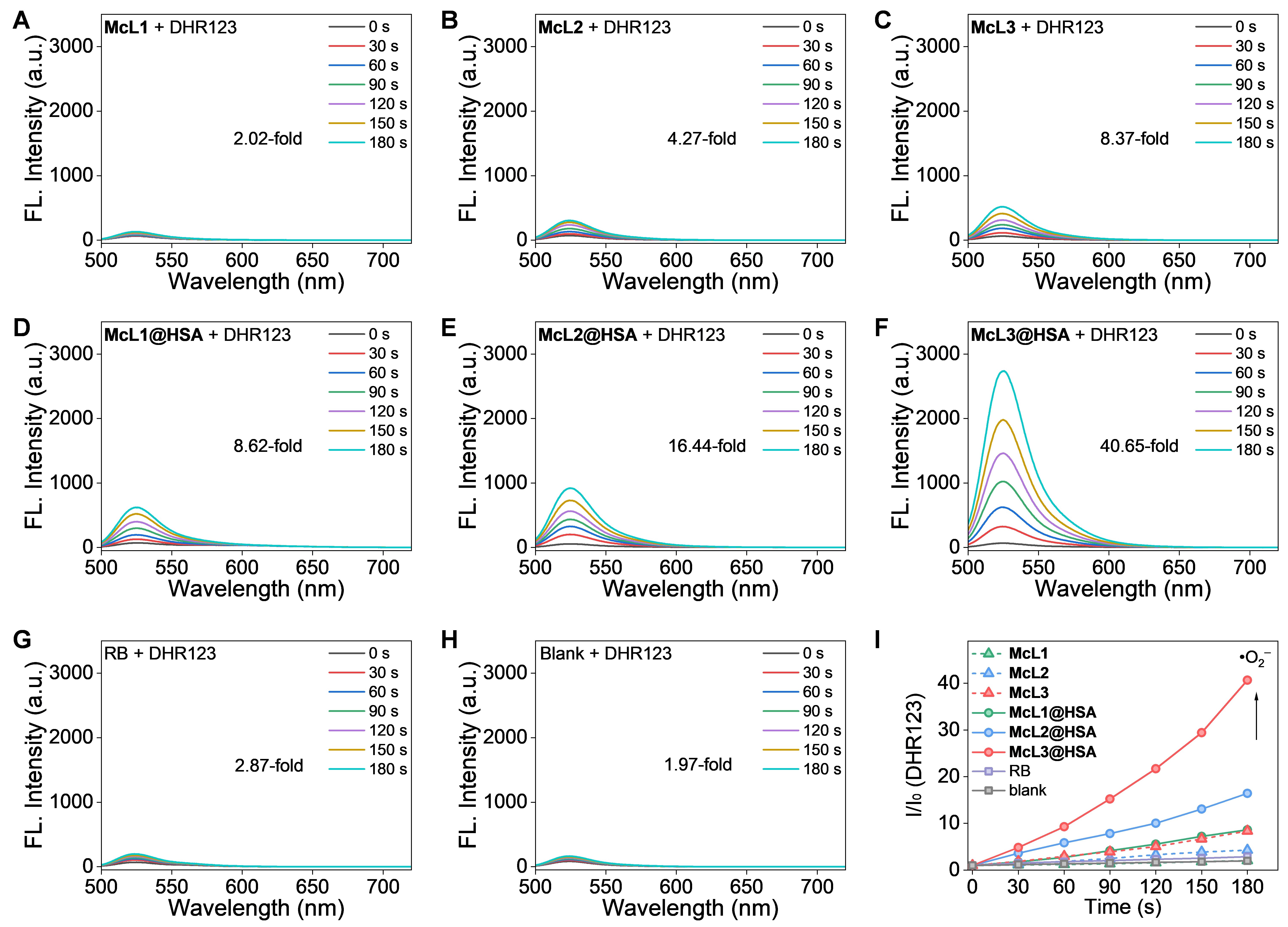


**Figure S13.** Fluorescence emission spectra of DHR123 (5 μM, λ_ex_ = 480 nm) mixed with (A) **McL1**, (B) **McL2**, (C) **McL3**, (D) **McL1@HSA**, (E) **McL2@HSA**, (F) **McL3@HSA**, (G) RB, (H) blank in PBS (10 mM, pH = 7.42) after white-light (420 nm long-pass filter, 80 mW cm^−2^) irradiation for different times. The sample concentration was adjusted to an absorbance of 0.2 OD. (I) Compare the production of •O_2_^‾^ after different irradiation time. I_0_ and I are the fluorescence intensity of DHR123 at 524 nm before and after irradiation, respectively.


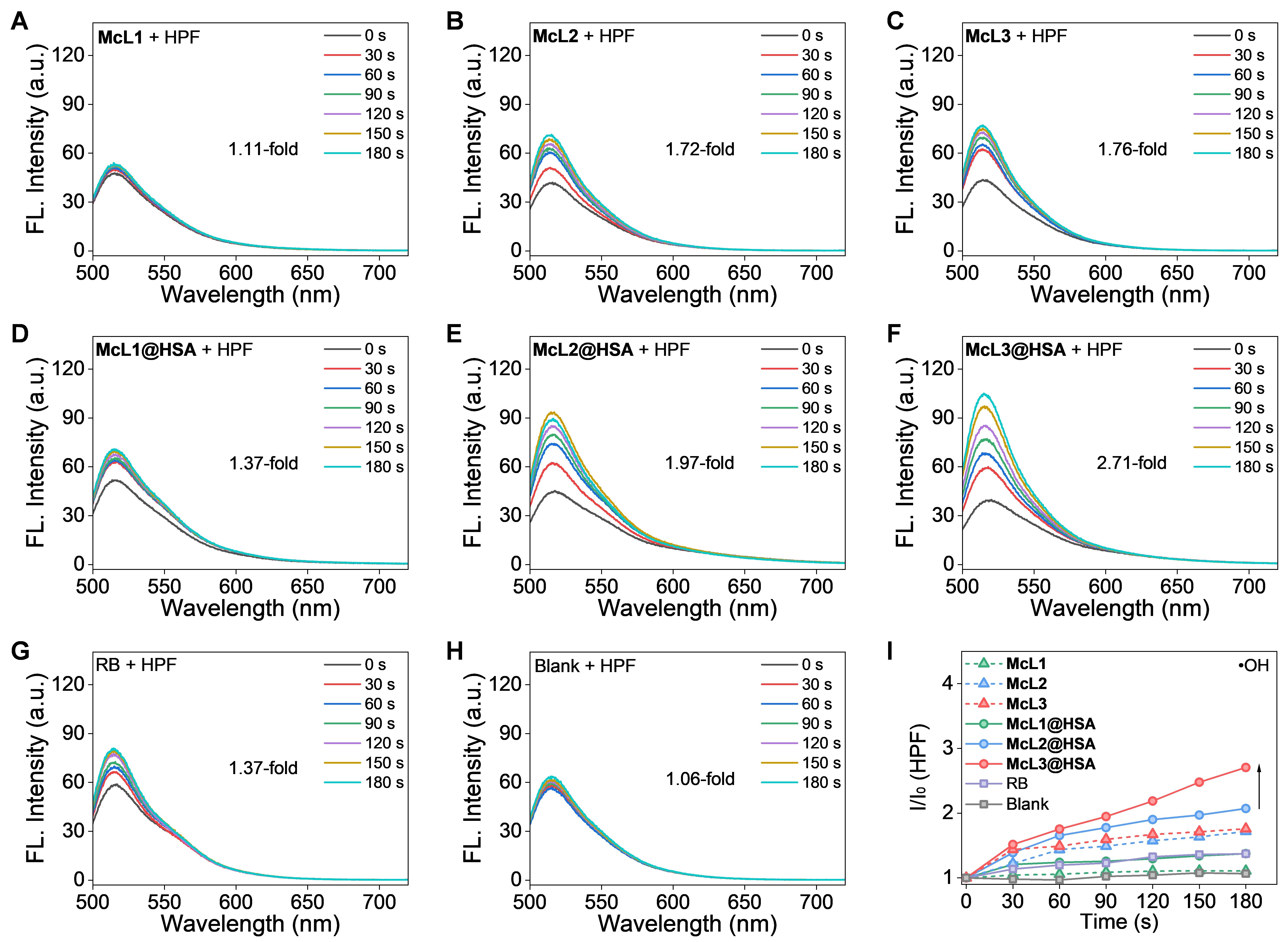


**Figure S14.** Fluorescence emission spectra of HPF (5 μM, λ_ex_ = 480 nm) mixed with (A) **McL1**, (B) **McL2**, (C) **McL3**, (D) **McL1@HSA**, (E) **McL2@HSA**, (F) **McL3@HSA**, (G) RB, (H) blank in PBS (10 mM, pH = 7.42) after white-light (420 nm long-pass filter, 80 mW cm^−2^) irradiation for different times. The sample concentration was adjusted to an absorbance of 0.2 OD. (I) Compare the production of •OH after different irradiation time. I_0_ and I are the fluorescence intensity of HPF at 515 nm before and after irradiation, respectively.


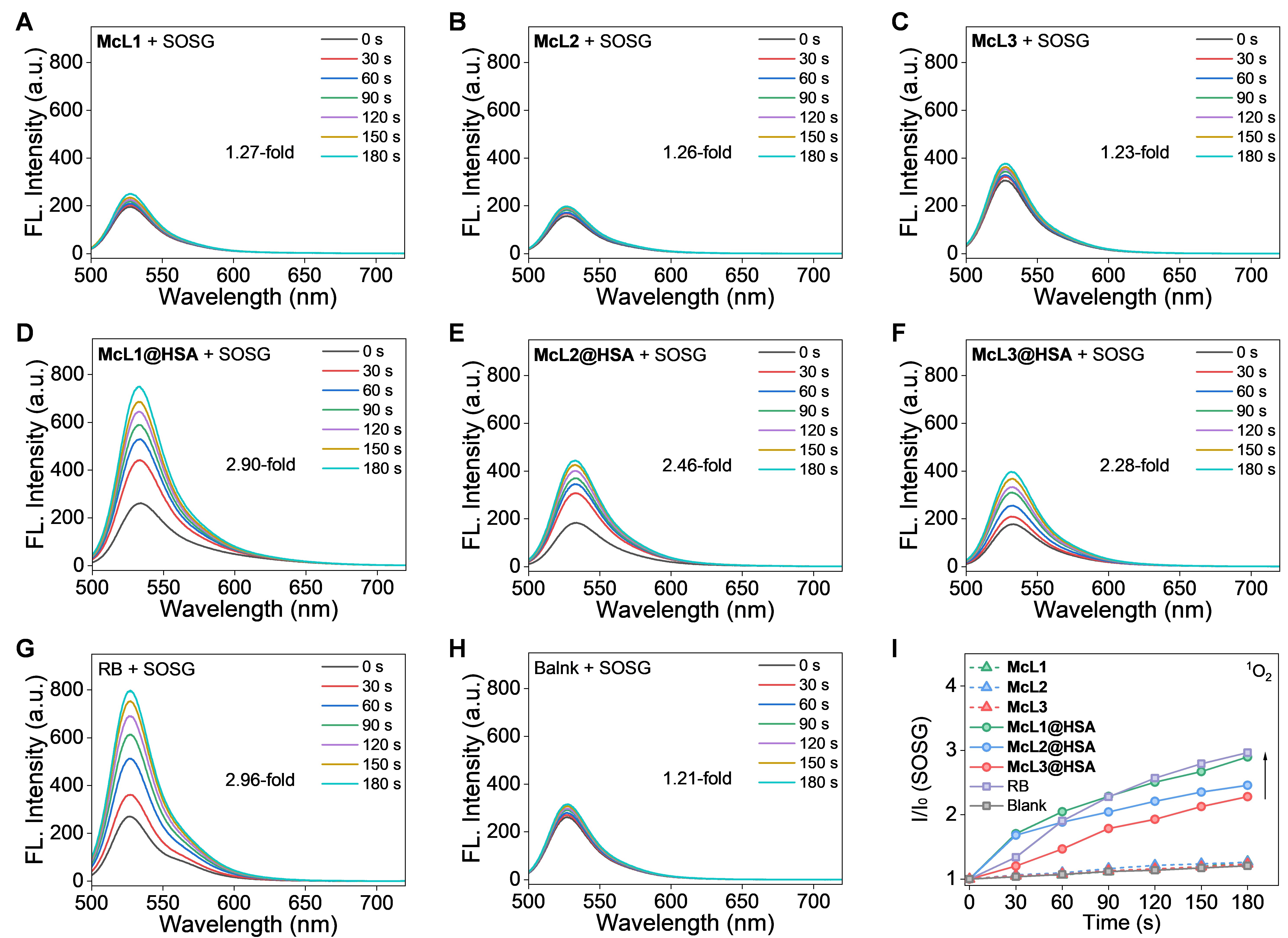


**Figure S15.** Fluorescence emission spectra of SOSG (5 μM, λ_ex_ = 480 nm) mixed with (A) **McL1**, (B) **McL2**, (C) **McL3**, (D) **McL1@HSA**, (E) **McL2@HSA**, (F) **McL3@HSA**, (g) RB, (H) blank in PBS (10 mM, pH = 7.42) after white-light (420 nm long-pass filter, 80 mW cm^−2^) irradiation for different times. The sample concentration was adjusted to an absorbance of 0.2 OD. (I) Compare the production of ^1^O_2_ after different irradiation time. I_0_ and I are the fluorescence intensity of SOSG at 527 nm before and after irradiation, respectively.


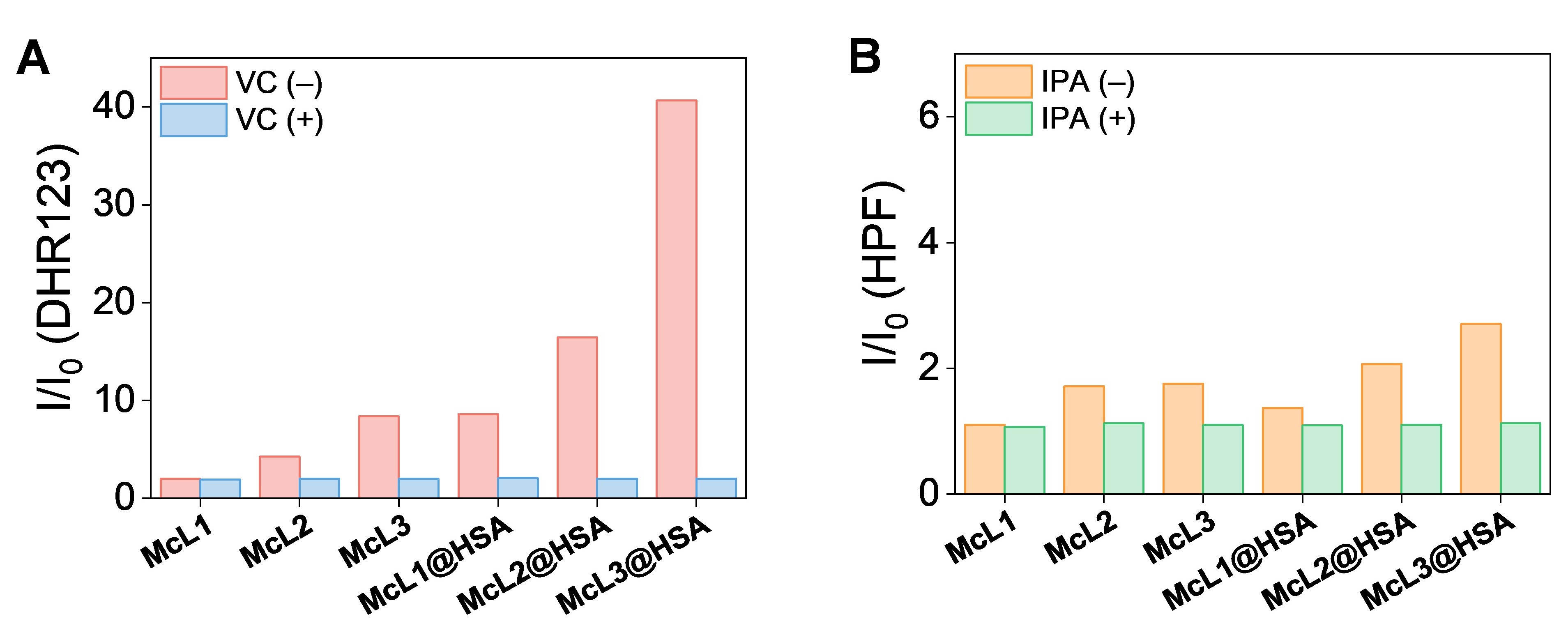


**Figure S16.** (A) Comparison of the fluorescence intensity of DHR123 in the presence of PSs, which mixed with (+) or without (−) VC after white-light (420 nm long-pass filter, 80 mW cm^−2^) irradiation for 180 s. (B) Comparison of the fluorescence intensity of HPF in the presence of PSs, which mixed with (+) or without (−) IPA after white-light (420 nm long-pass filter, 80 mW cm^−2^) irradiation for 180 s.


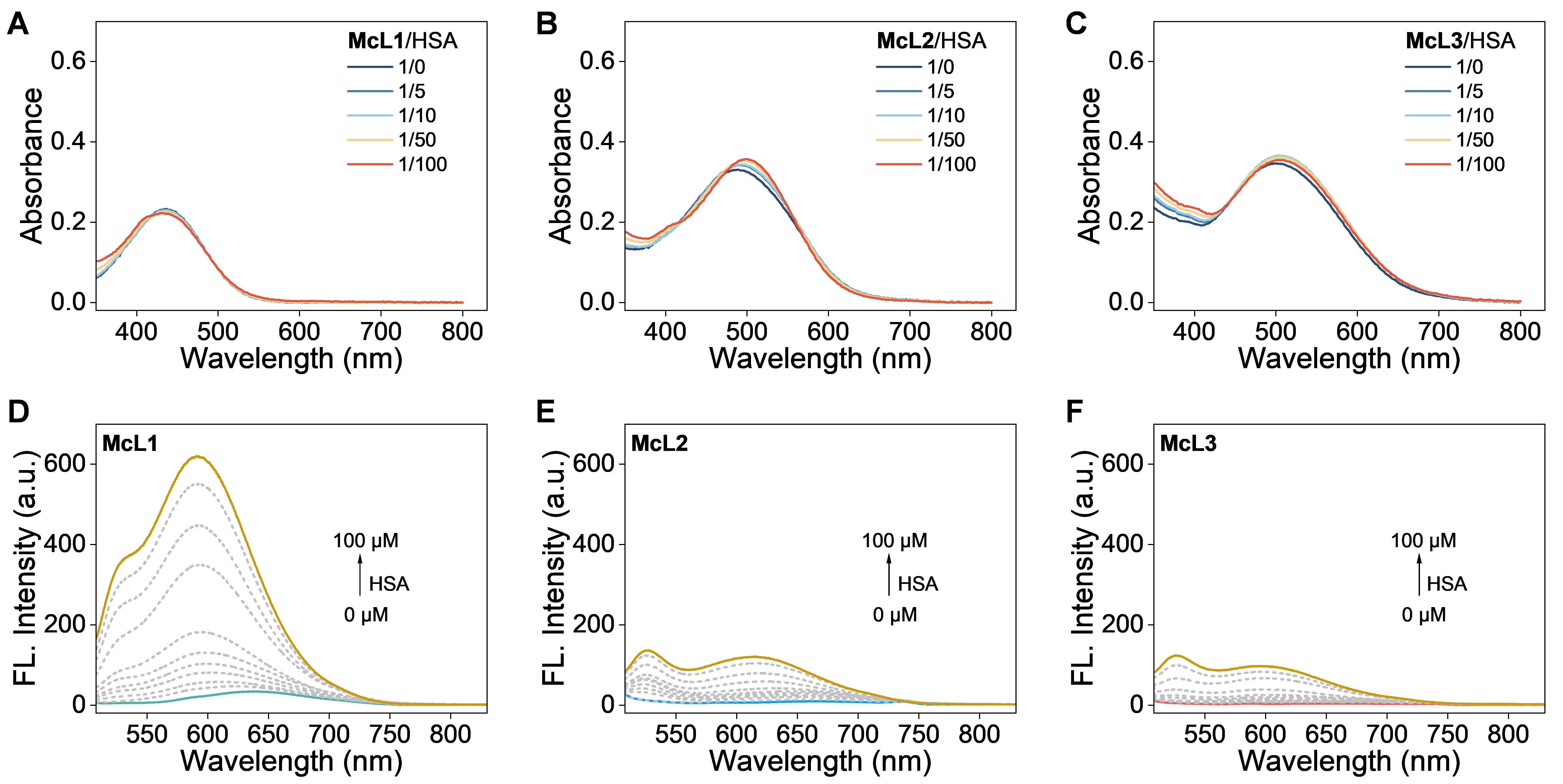


**Figure S17.** (A–C) UV-*vis* absorption and (D–F) fluorescence emission spectra of **McL1**–**3** (10 μM) with different concentrations of HSA in PBS (10 mM, pH = 7.42).


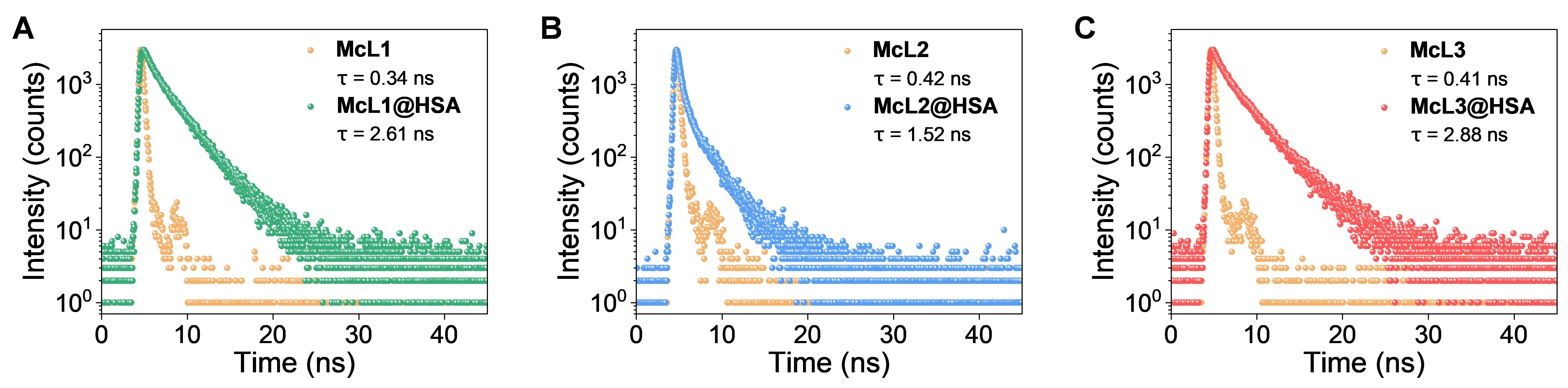


**Figure S18.** Time-resolved decay profiles of (A) **McL1** and **McL1@HSA**, (B) **McL2** and **McL2@HSA**, (C) **McL3** and **McL3@HSA** in PBS (10 mM, pH = 7.42) with excited by EPL picosecond pulsed diode lasers at 450 nm.


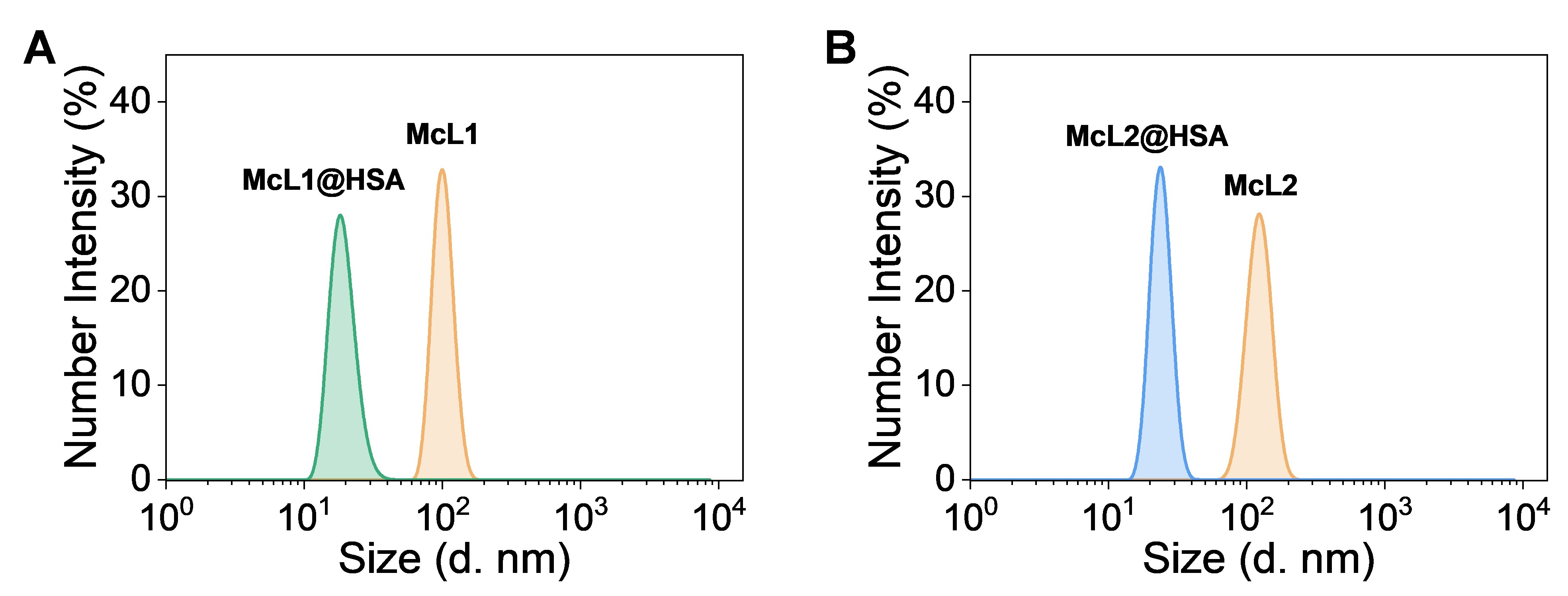


**Figure S19.** The hydrodynamic distribution curves of (A) **McL1**, **McL1@HSA** and (B) **McL2**, **McL2@HSA** were determined by DLS.


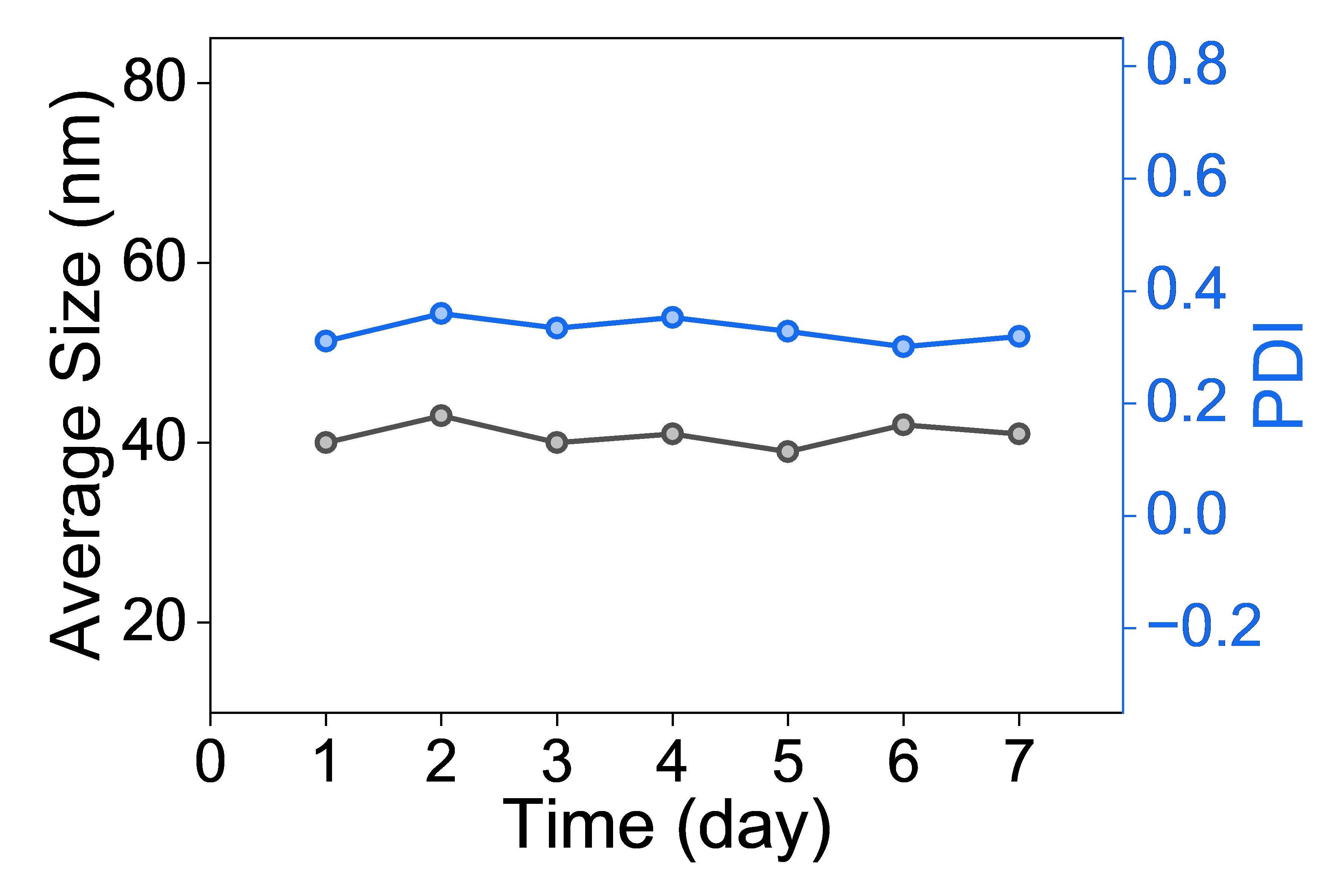


**Figure S20.** The colloidal stability of **McL3@HSA** in PBS (10 mM, pH = 7.42) for different storage periods at 4°C.


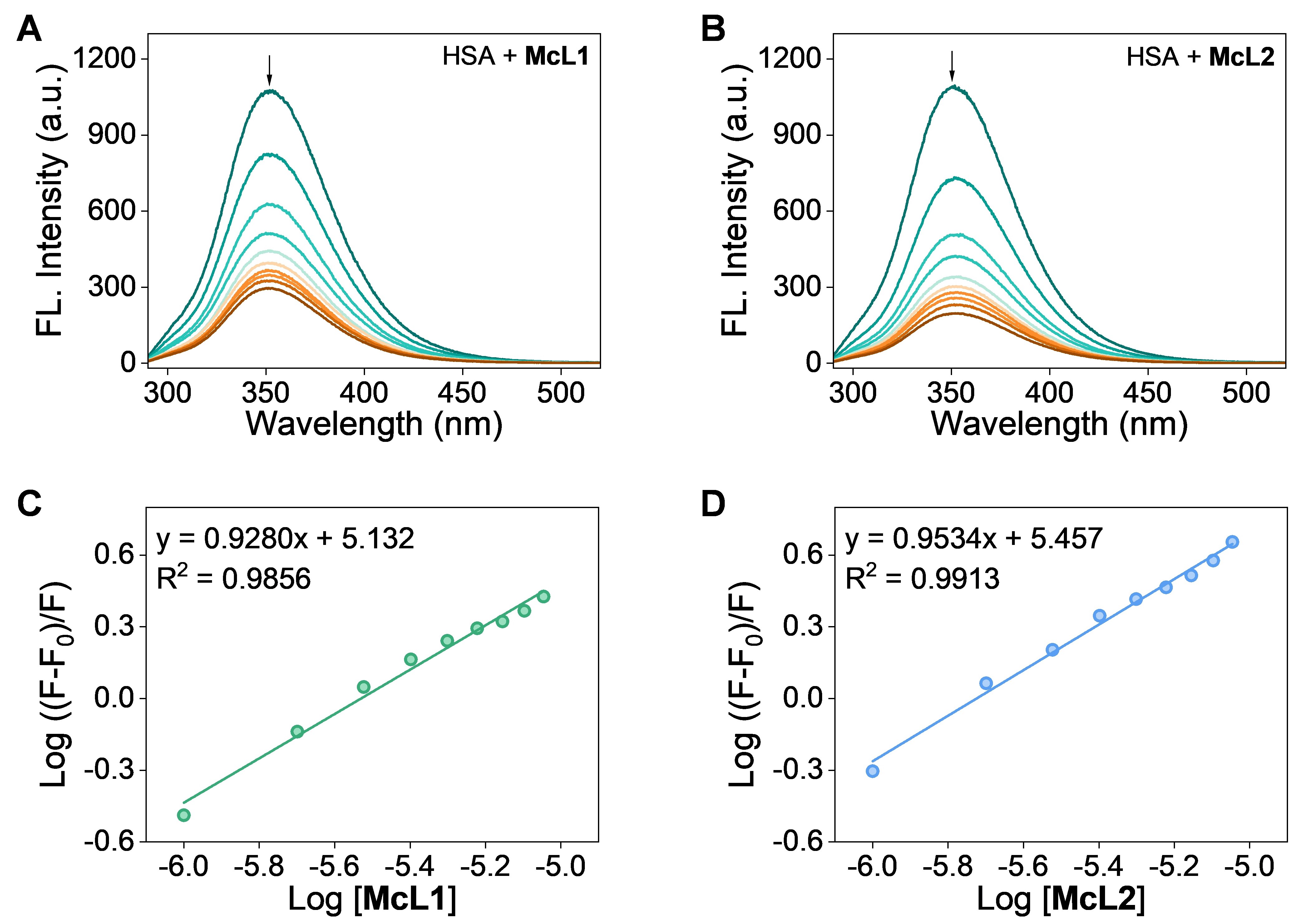


**Figure S21.** The fluorescence emission spectra of HSA (10 μM, λ_ex_ =280 nm) quenching induced by (A) **McL1** and (B) **McL2**, respectively. Double-logarithmic regression curves of (C) **McL1** and (D) **McL2**, respectively. **McL1** and **McL2** exhibit a high binding affinity for HSA with a binding constant greater than 10^5^ M^-1^ and a binding stoichiometry of 1:1 as determined by fluorescence titration.


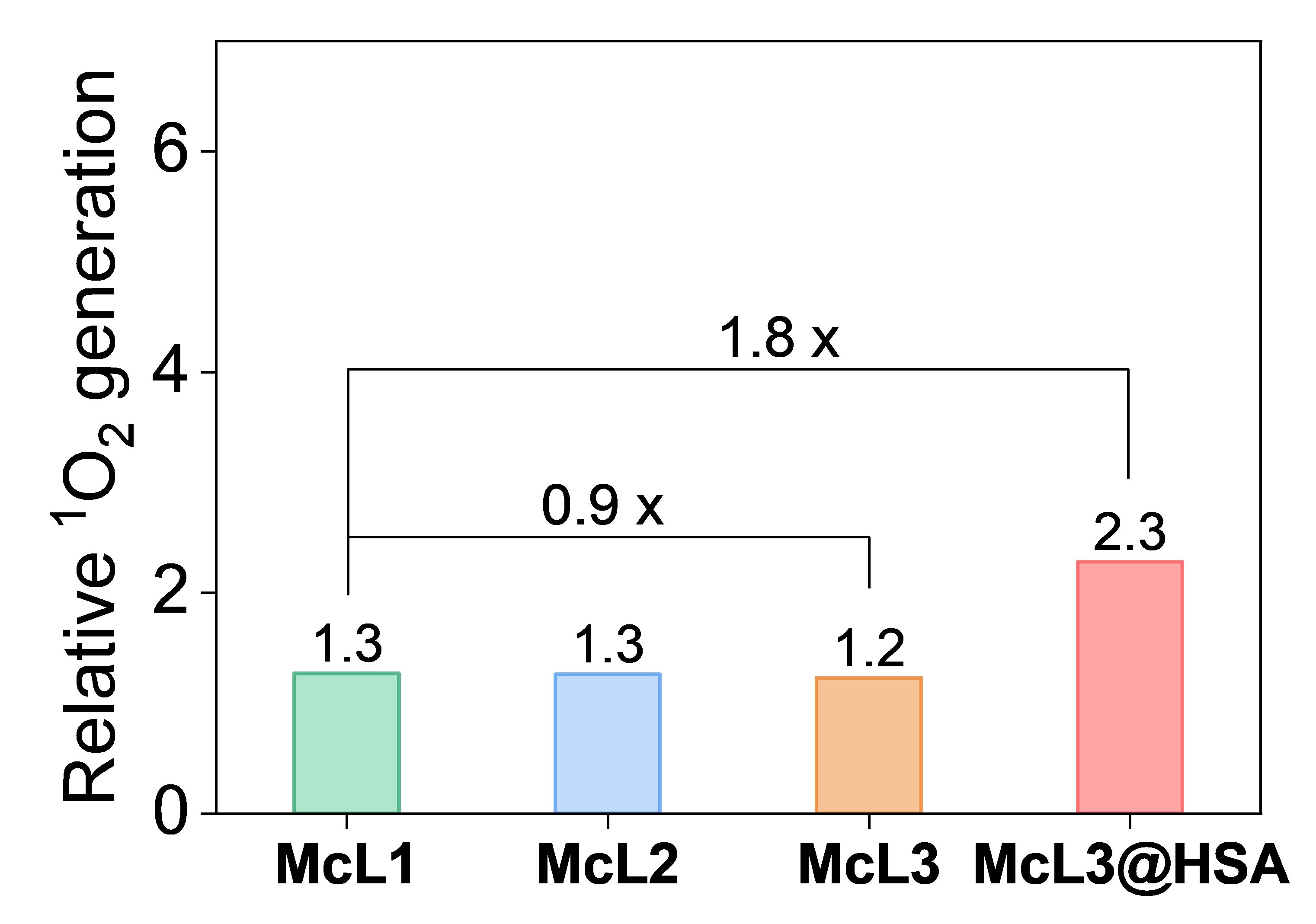


**Figure S22.** Comparative ^1^O_2_ generation abilities of **McL1**–**3** and **McL3@HSA** measured by the fluorescence increase of SOSG after 180 s white-light irradiation (420 nm long-pass filter, 80 mW cm^−2^). Data are expressed as fold-change in fluorescence intensity.


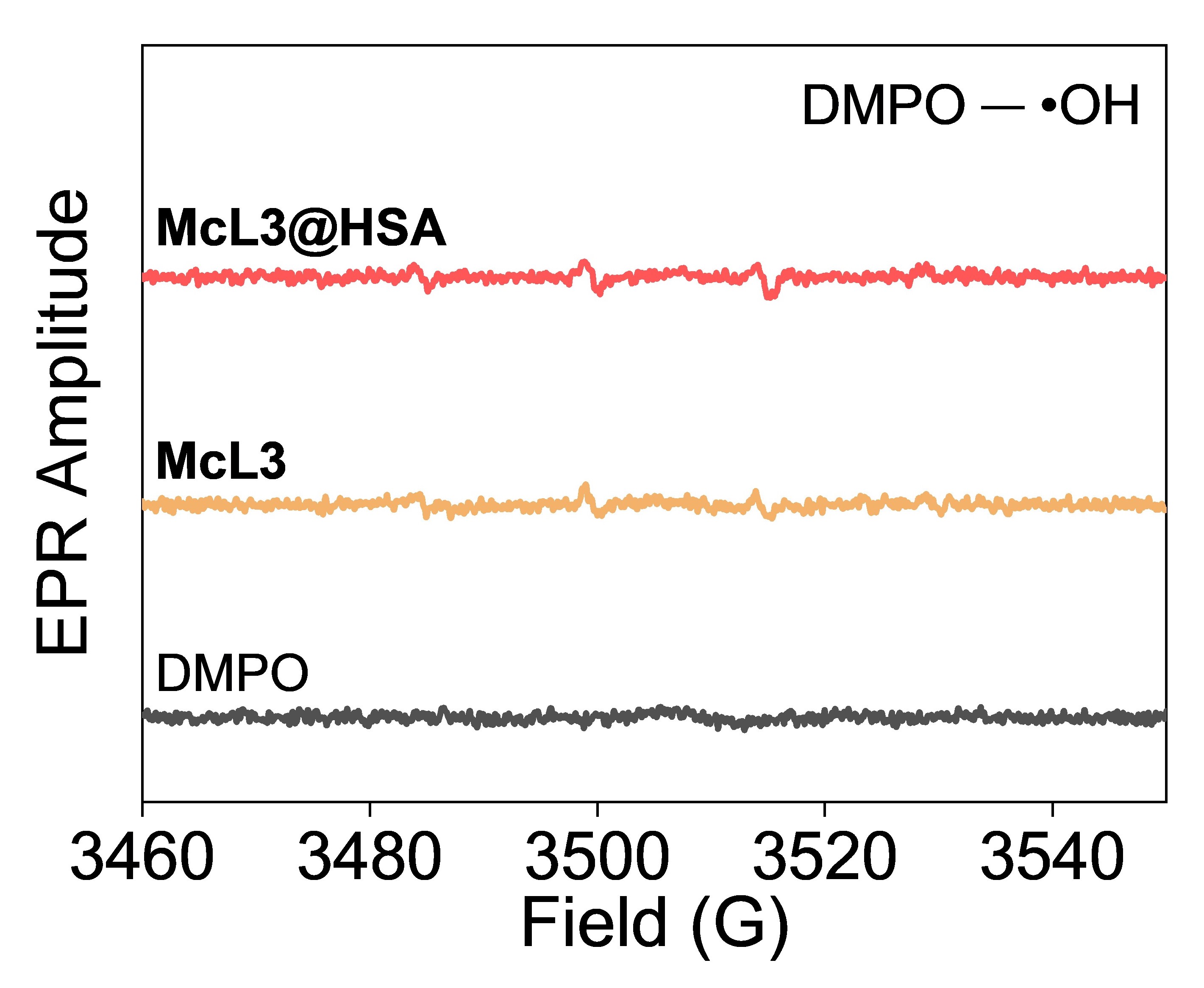


**Figure S23.** Electron paramagnetic resonance (EPR) signals of **McL3** and **McL3@HSA** under white-light irradiation, using DMPO as a spin trap for •OH.


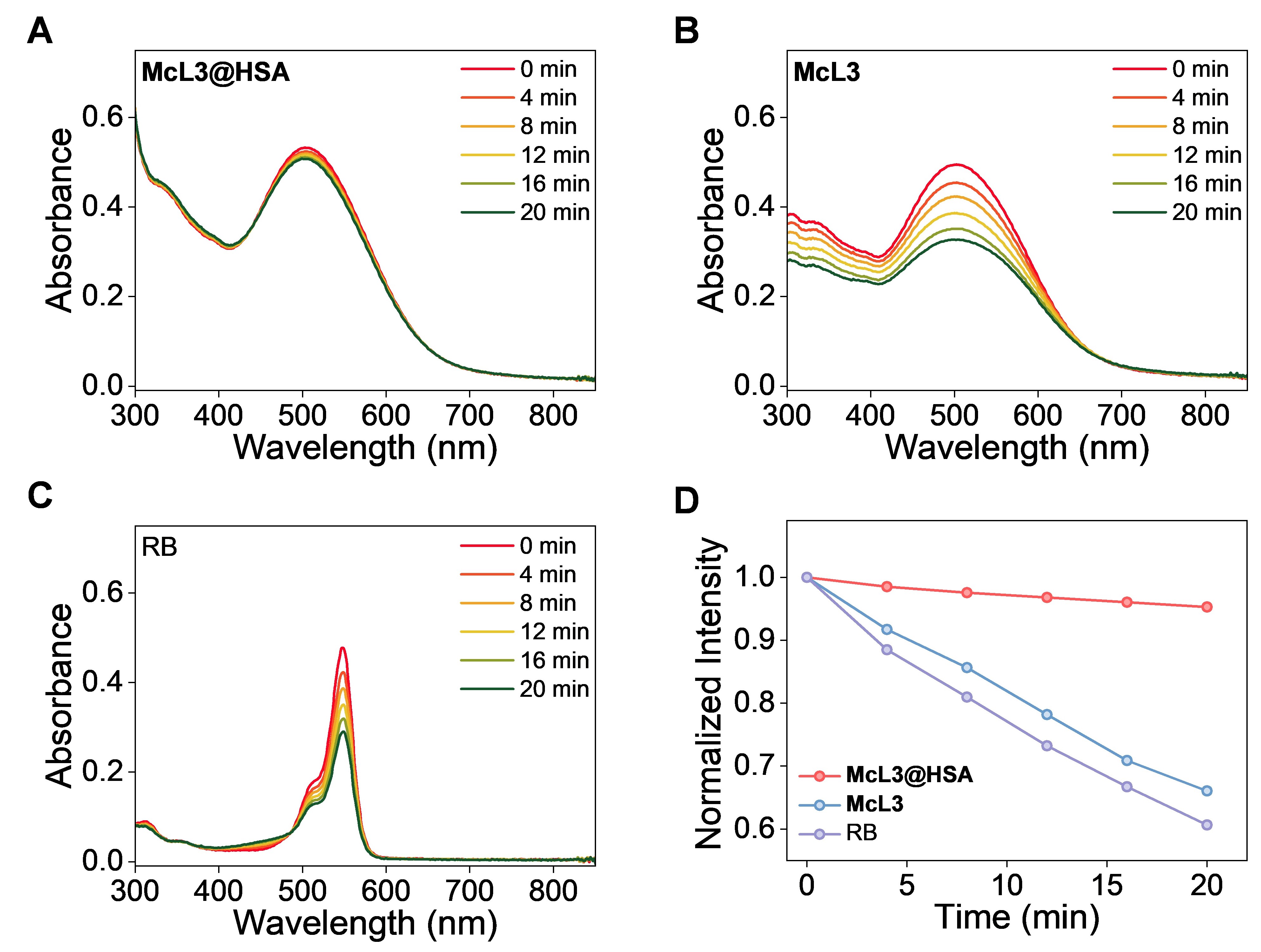


**Figure S24.** (A–C) The absorption spectra and (D) the corresponding absorbance intensity changes of **McL3@HSA**, **McL3**, and RB in PBS (10 mM, pH = 7.42) upon white-light (420 nm long-pass filter, 80 mW cm^−2^) irradiation for 0 to 20 mins.


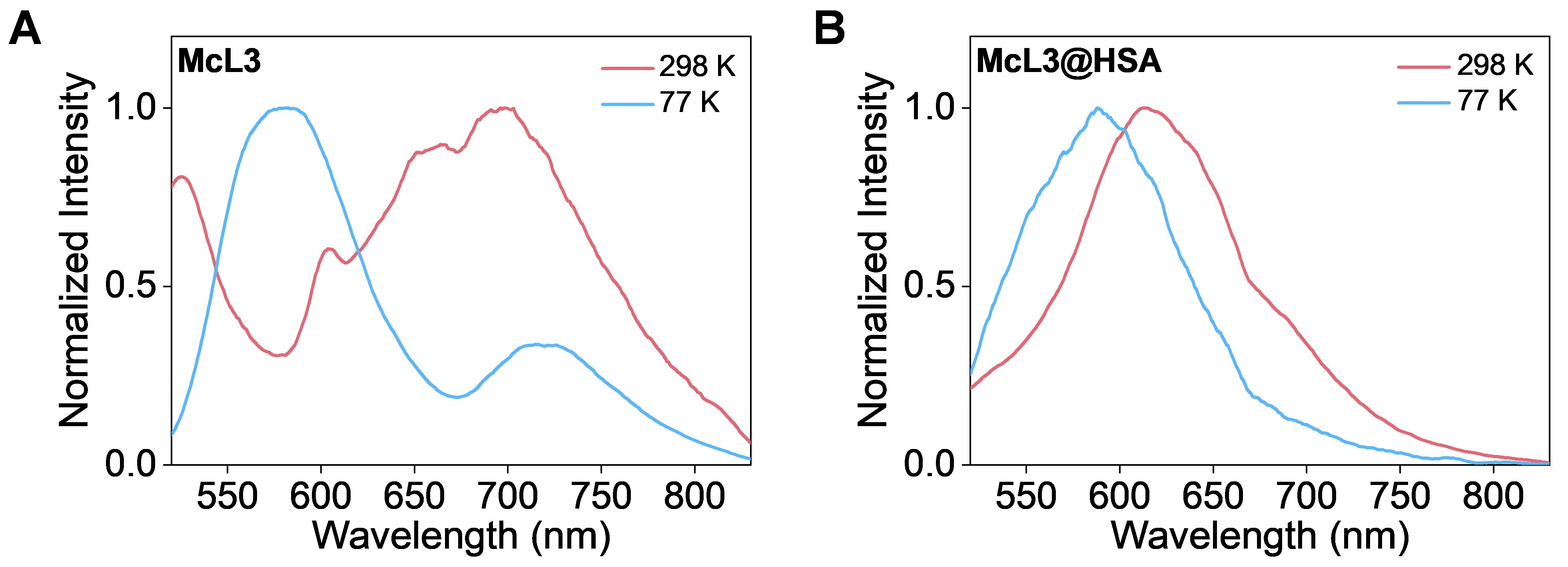


**Figure S25.** Normalized steady-state photoluminescence spectra of (A) **McL3** and (B) **McL3@HSA** in PBS (10 mM, pH = 7.42) at 298 K (red line) and 77 K (blue line), respectively.


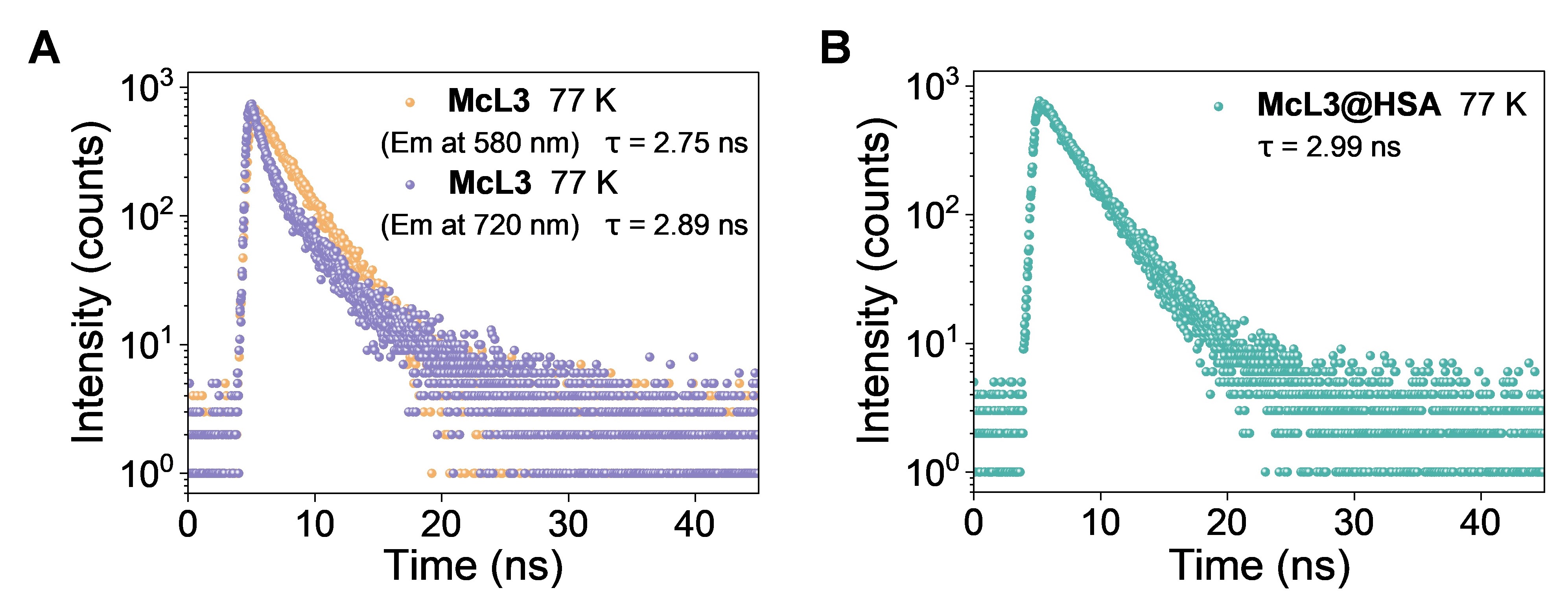


**Figure S26.** Lifetime decay profiles of (A) **McL3** and (B) **McL3@HSA** in PBS (10 mM, pH = 7.42) upon excitation with 450 nm at 77 K. The fluorescence lifetimes of **McL3** almost consistently with emission peaks at 580 and 720 nm at 77 K, indicating the fluorescent nature of the emission, further proving that the restriction of molecular vibrations at low temperatures is the same as that of HSA protein cavities.


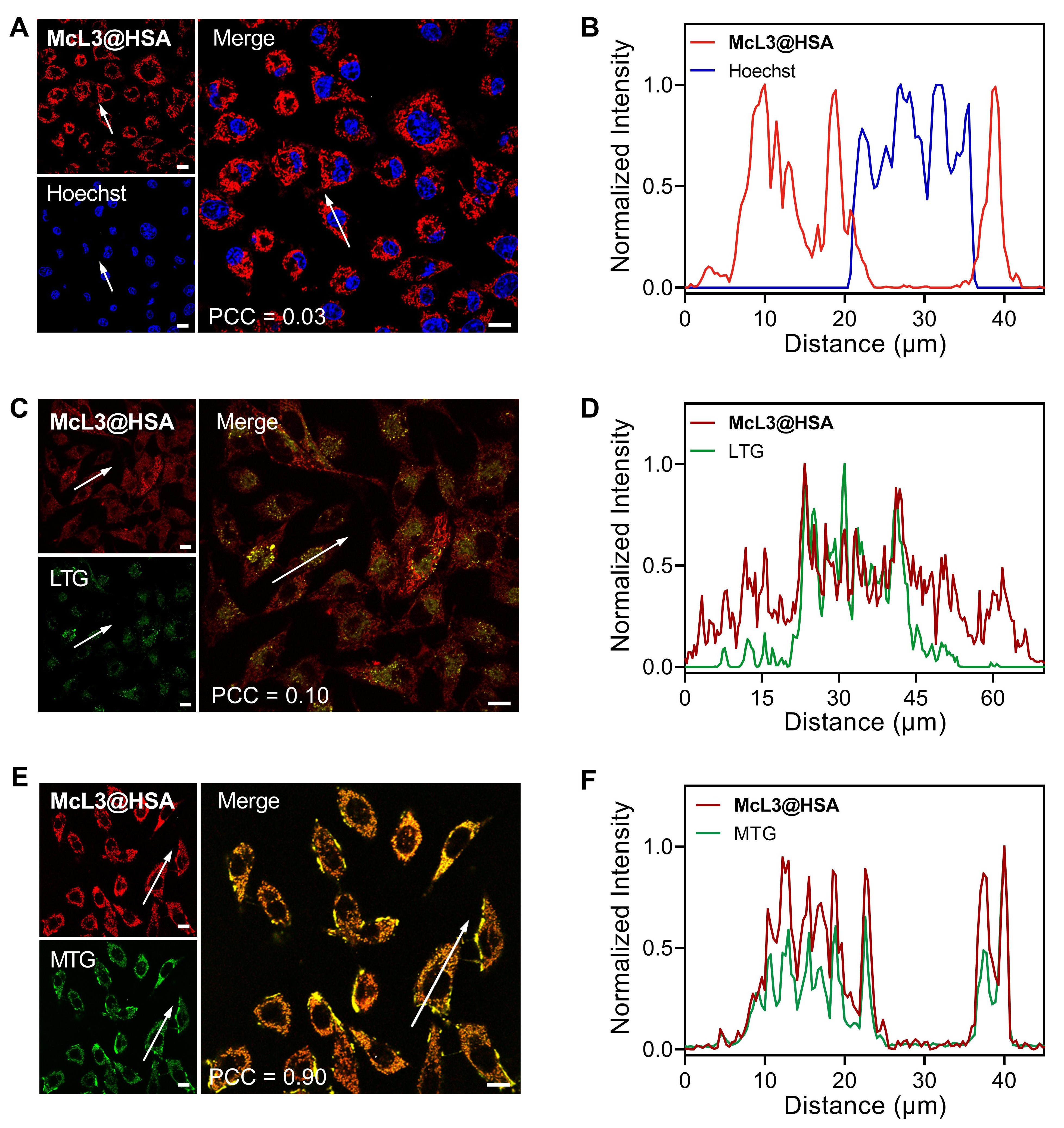


**Figure S27.** Fluorescence colocalization images of **McL3@HSA** (1 μM) co-stained with (A) Hoechst 33342 (Hoechst, 10 μg/mL), (C) LysoTracker Green (LTG, 100 nM), and (E) MitoTracker Green (MTG, 100 nM). Inset: Pearson correlation coefficient (PCC). Scale bar = 10 μm. (B,D,F) The normalized fluorescence intensities profiles in (A), (C) and (E) along the white lines, respectively. Imaging conditions: **McL3@HSA** (red channel): λ_ex/em_ = 488/550–750 nm; Hoechst (blue channel): λ_ex/em_ = 405/440–480 nm; LTG (green channel): λ_ex/em_ = 488/500–540 nm; MTG (green channel): λ_ex/em_ = 488/500–540 nm.


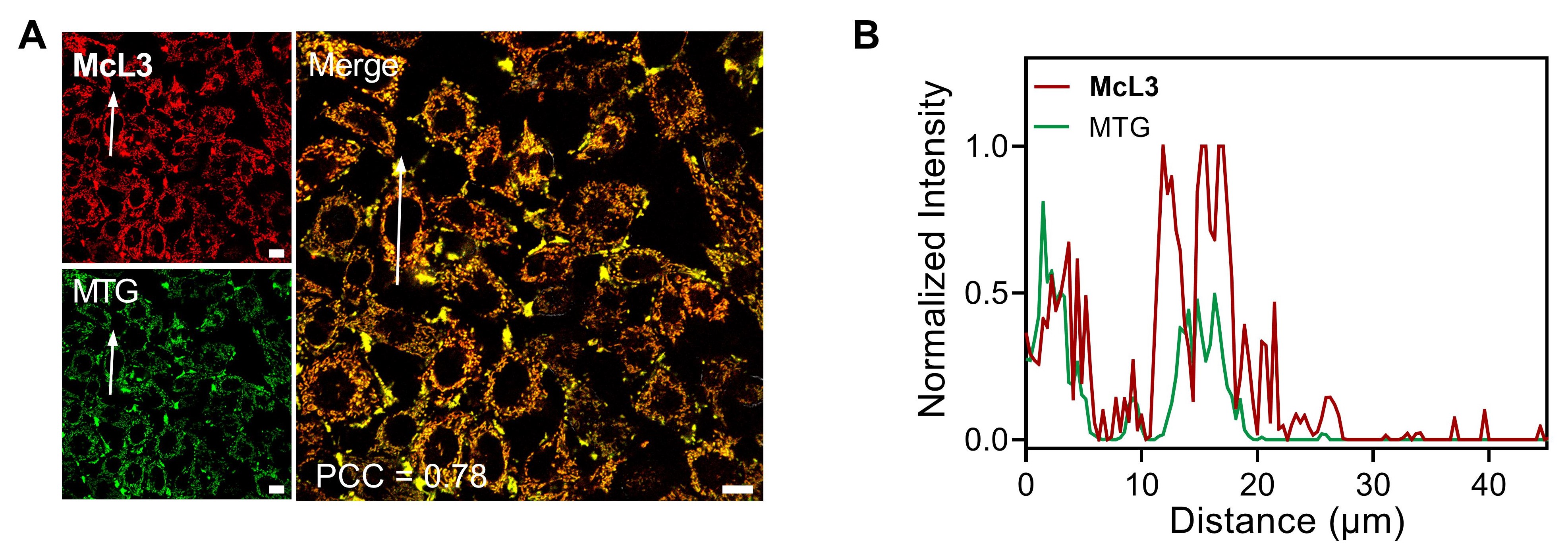


**Figure S28.** (A) Fluorescence colocalization images of **McL3** (λ_ex/em_ = 488/550–750 nm) co-stained with MitoTracker Green (MTG, 100 nM). Inset: Pearson correlation coefficient (PCC). Scale bar = 10 μm. (B) The normalized fluorescence intensities profiles in (A) along the white lines. Imaging conditions: **McL3** (red channel): λ_ex/em_ = 488/550–750 nm; MTG (green channel): λ_ex/em_ = 488/500–540 nm.


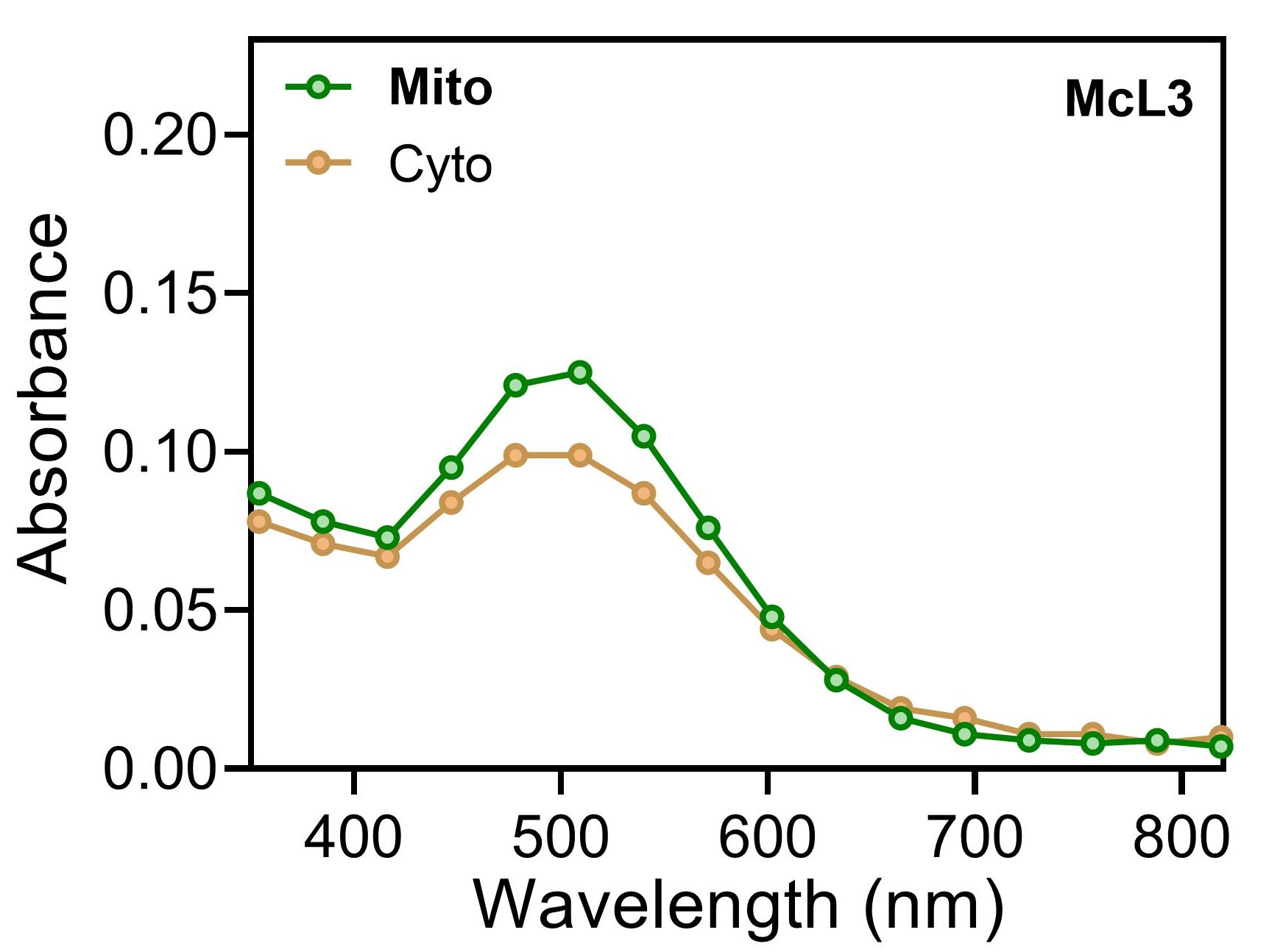


**Figure S29.** The absorption spectra of **McL3** in mitochondrial and cytoplasmic fractions.


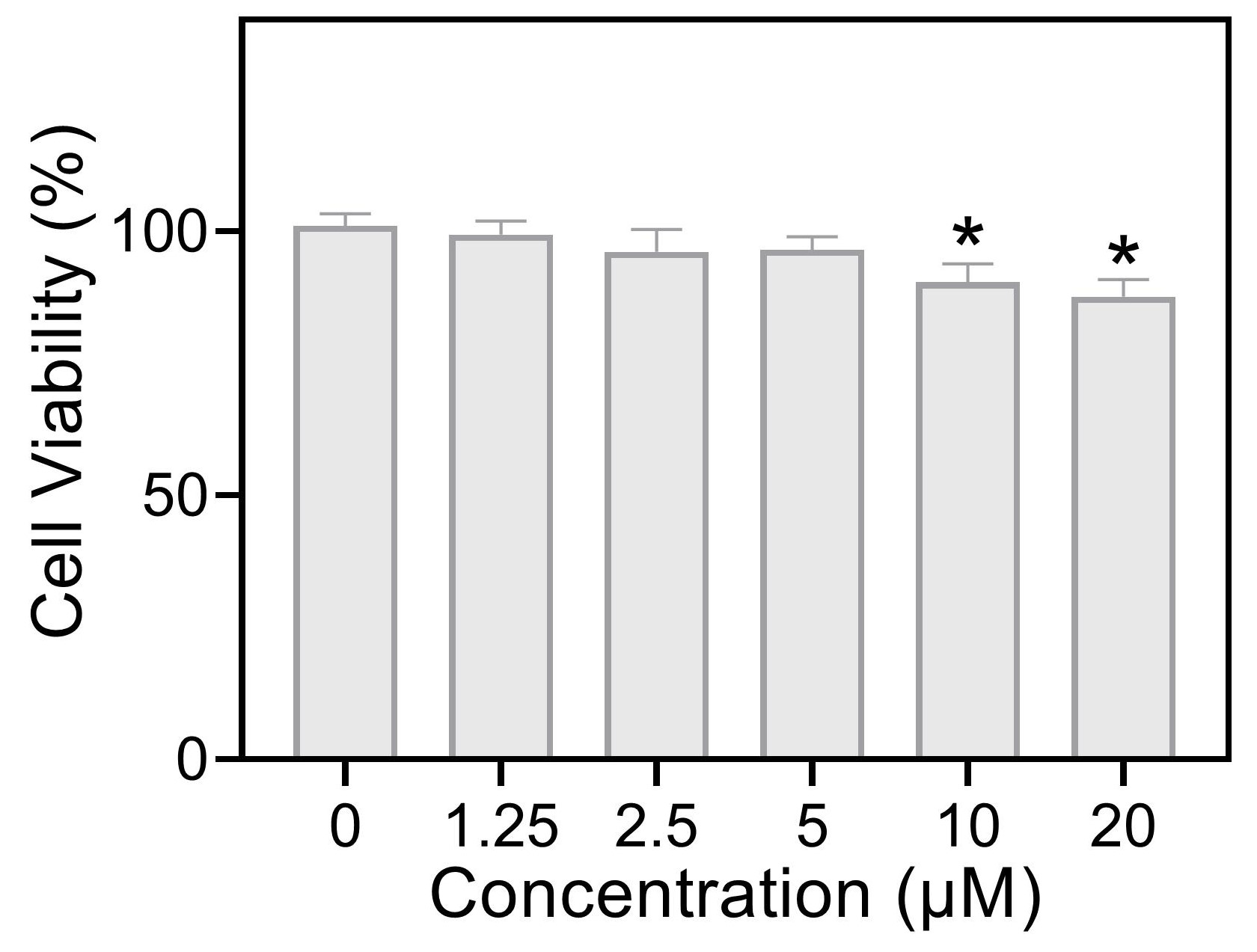


**Figure S30.** Cell viability of A549 cells treated with **McL3@HSA** (0–20 μM). Data were presented as mean ± SD (*n* = 6). Statistical significance: **P* < 0.05 *vs*. 0 μM group.


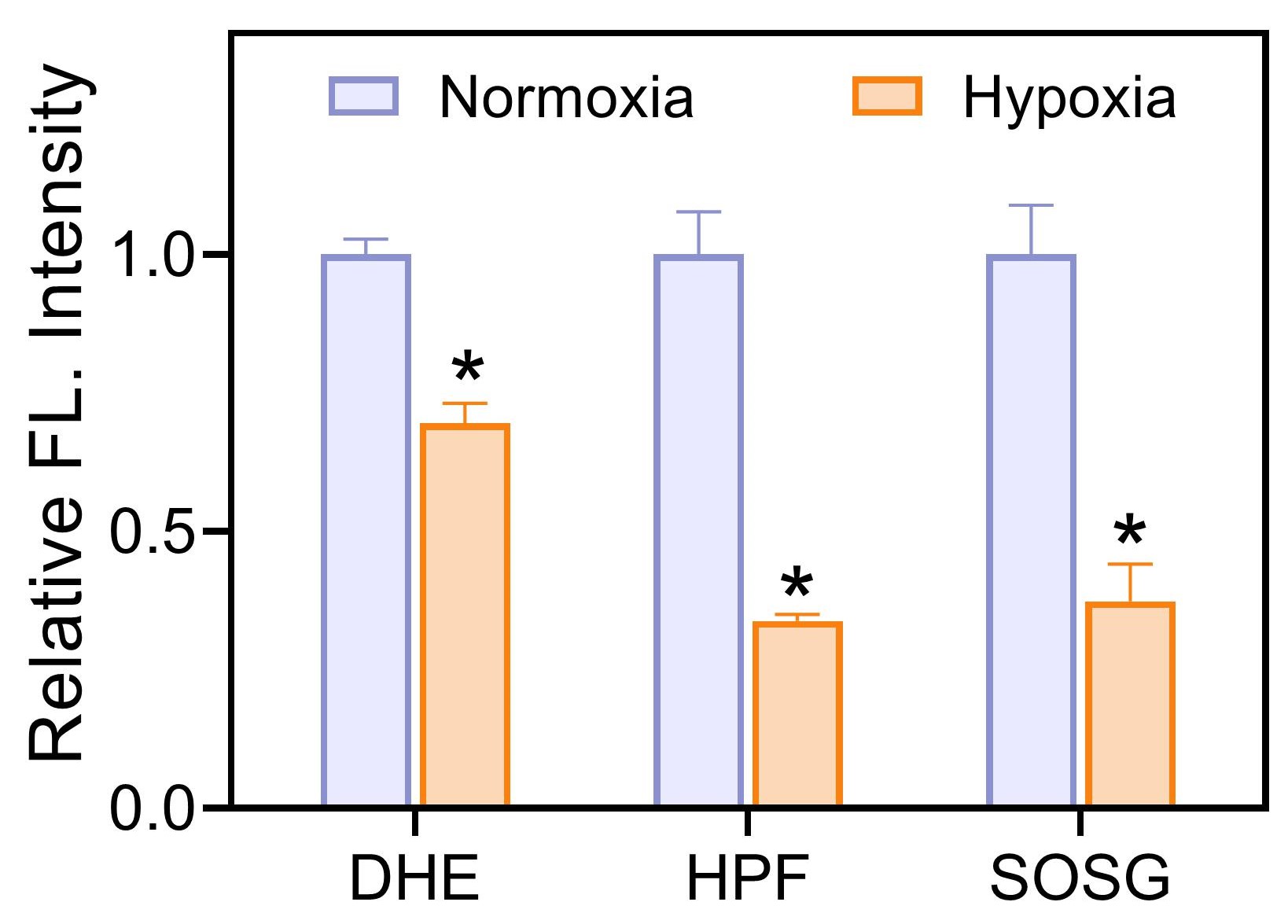


**Figure S31.** The fluorescence intensity quantification of intracellular ROS detected by commercial ROS indicators (DHE, HPF and SOSG) in the presence of **McL3@HSA** (1 μM) under white-light (420 nm long-pass filter, 80 mW cm^−2^, 5 mins) in both normoxic and hypoxic condition. Data were presented as mean ± SD (*n* = 3). Statistical significance: **P* < 0.05 *vs*. normoxia group.


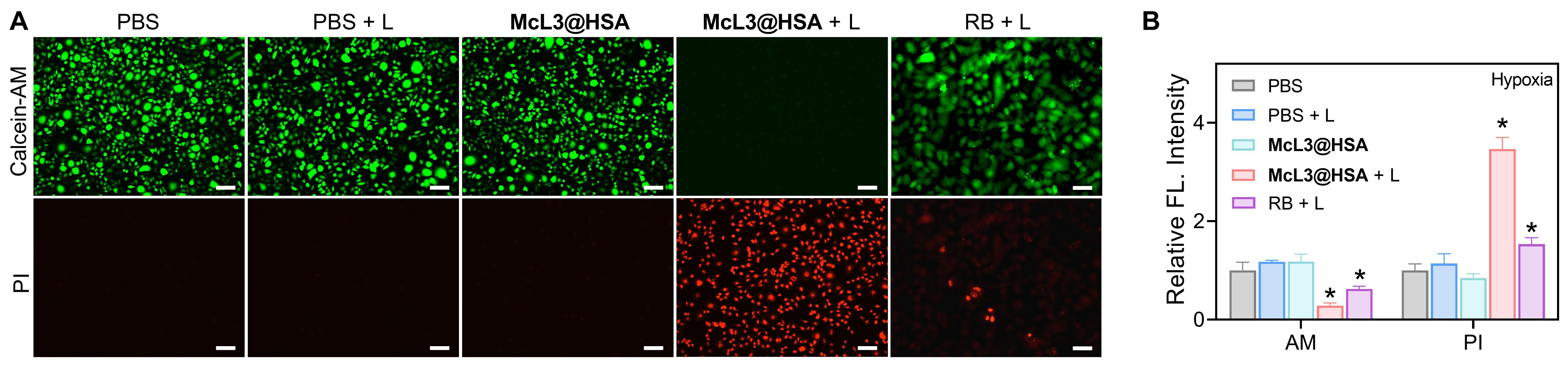


**Figure S32.** (A) Representative live/dead fluorescence images of A549 cells under hypoxic conditions after various treatments, stained with Calcein-AM/PI (green: live cells; red: dead cells). Concentrations of **McL3@HSA** and RB were 5 μM, respectively. Scale bar = 100 μm. (B) Quantification of fluorescence intensity from the live/dead cell staining in (A). Data were presented as mean ± SD (*n* = 3). Statistical significance: **P* < 0.05 *vs.* PBS group.


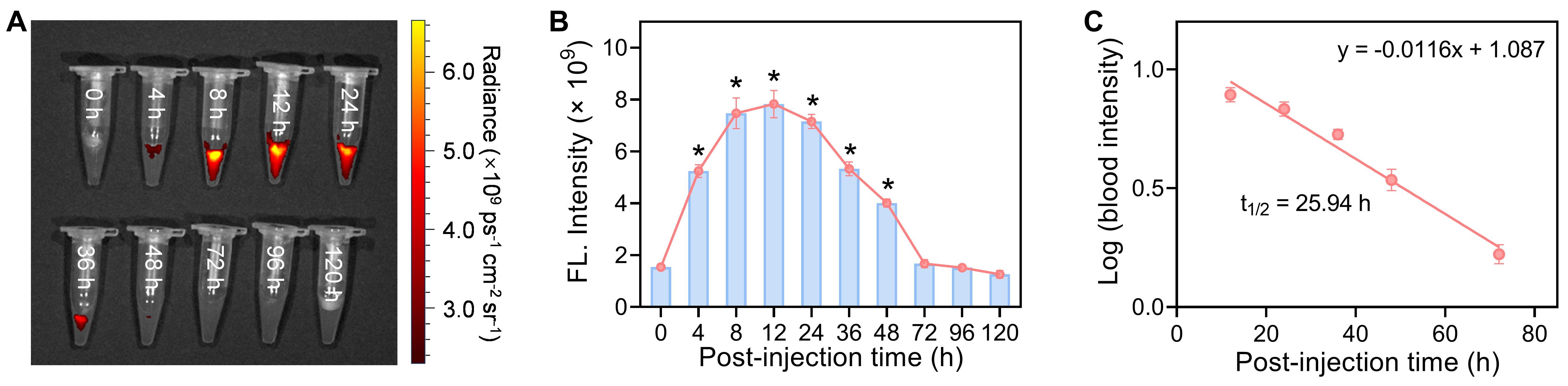


**Figure S33.** (A) The fluorescence images of blood sample collected from A549 tumor-bearing mice with the i.v. injection of **McL3@HSA** (0, 4, 8, 12, 24, 36, 48, 72, 96, 120 hrs). (B) Quantificated the fluorescence intensities of blood sample. Data were presented as mean ± SD (*n* = 3). Statistical significance: **P* < 0.05 *vs.* 0 hr group. (C) Comprehensive pharmacokinetic analysis based on the half-life of **McL3@HSA** degradation in mice blood.


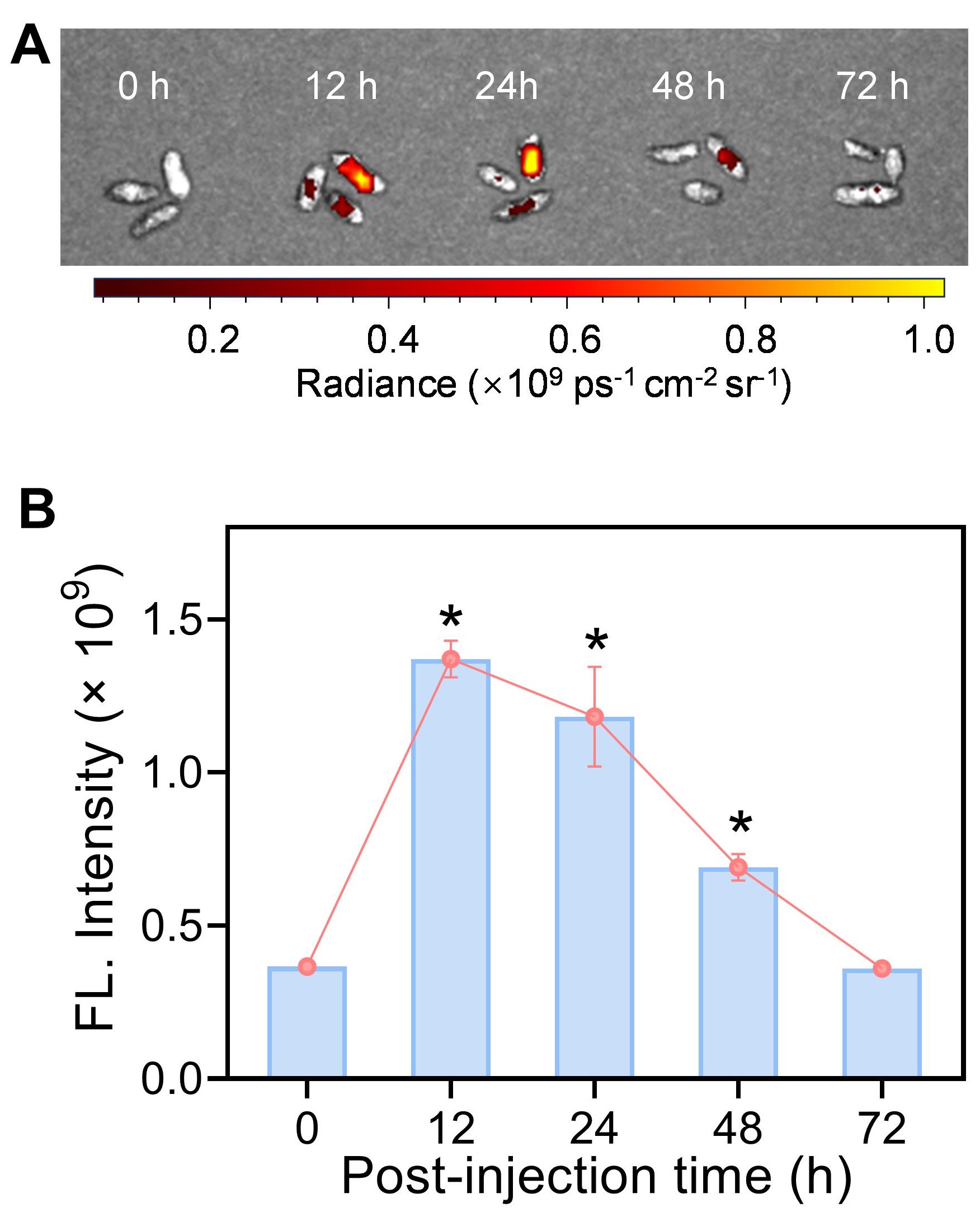


**Figure S34.** (A) Fluorescence images of faeces from A549 tumor-bearing mice following i.v. injection of **McL3@HSA** over a 72 hrs period. (B) Quantitative analysis of the fluorescence intensity of fecal metabolites in (A). Data were presented as mean ± SD (*n* = 3). Statistical significance: **P* < 0.05 *vs.* 0 hr group.


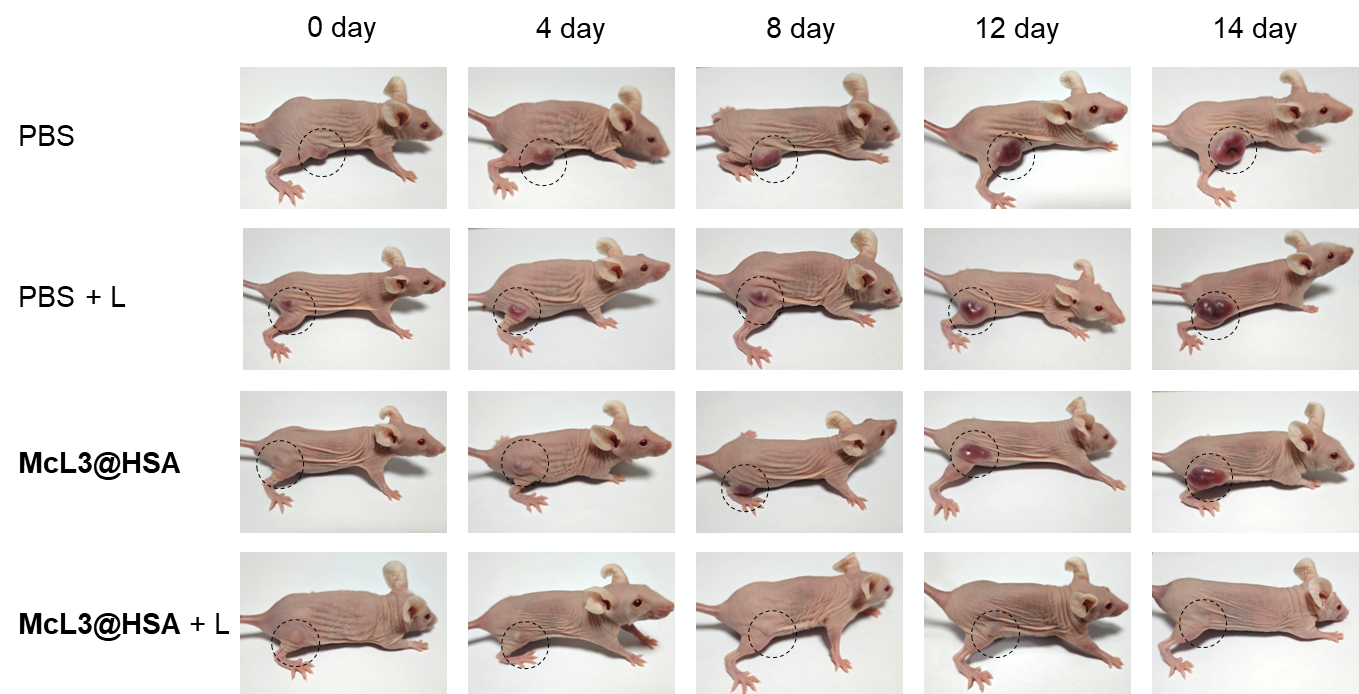


**Figure S35.** Representative photographs of A549 tumor-bearing mice from each group during treatment.


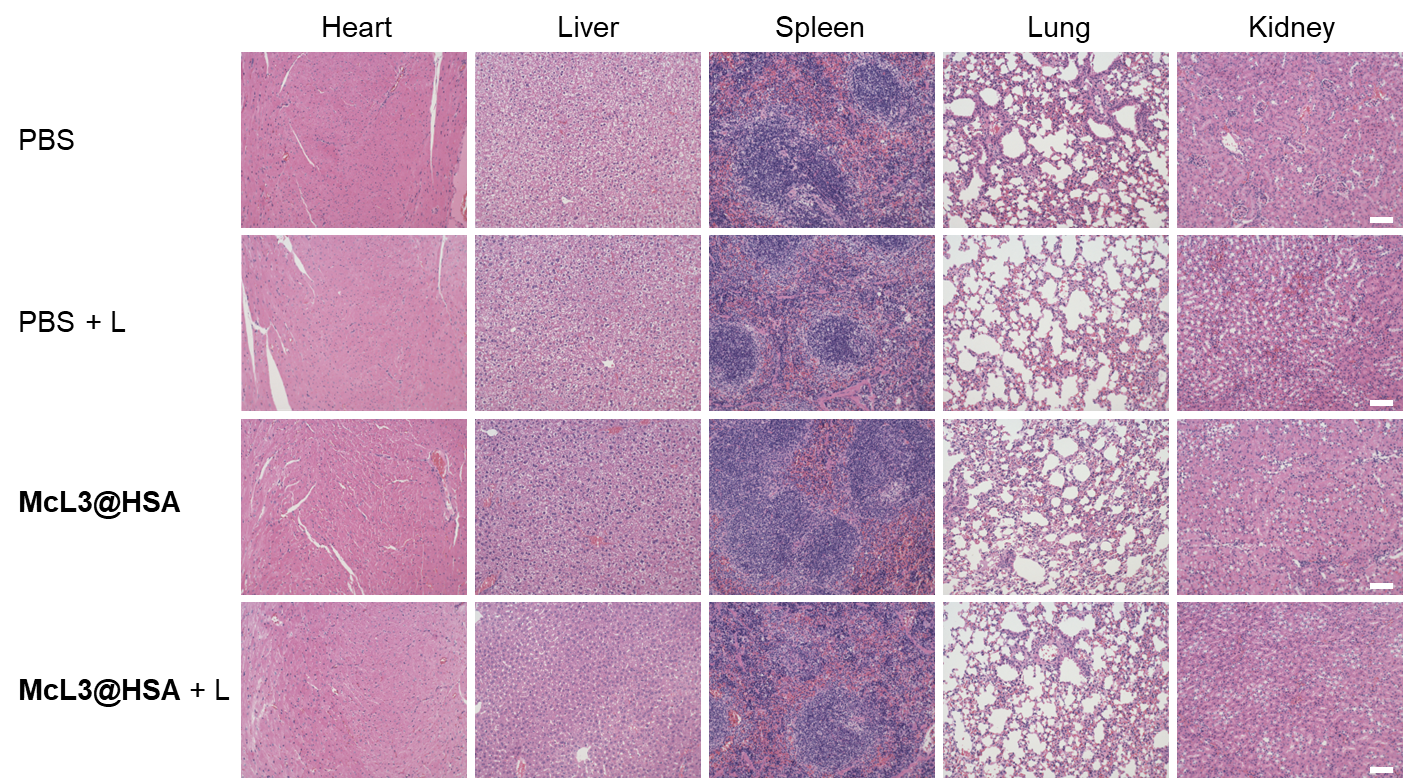


**Figure S36.** Representative images of H&E staining of heart, liver, spleen, lungs, and kidneys tissues of mice in different treatment groups. Scale bar = 50 μm.

## Supplementary tables

**Table S1.** ^1^H NMR spectroscopic data for the vinylene protons of **McL1**–**3** (500 MHz, 298 K, DMSO-*d_6_*).

| **Chemical structutre** | **δ_H_ (ppm)**^[a]^ |
| --- | --- |
|  | H_1_: Signal overlaped in 7.86 – 7.77 (m, 2H)  H_2_: 7.54 (d, *J* = 15.9 Hz, 1H) |
|  | H_1_ and H_3_: 7.86 (d, *J* = 15.7 Hz, 2H)  H_2_ and H_4_: 7.63 (d, *J* = 15.8 Hz, 2H) |
|  | H_1_ and H_2_: Signal overlaped in 8.10 – 7.69 (m, 14H)  H_3_ and H_5_: 7.58 (d, *J* = 15.5 Hz, 2H),  H_4_ and H_6_: 7.43 (d, *J* = 15.8 Hz, 2H) |

^[a]^ Vinylene protons in **McL1**–**3** exhibited chemical shifts (δ) in the range of 7.4 – 7.8 ppm with coupling constants (*J*) exceeding 15 Hz, indicating the formation of the *E*-configured products.

**Table S2.** Photophysical properties of **McL1**–**3** in different solvents.

| Sample | solvents | λ_abs_^[a]^ (nm) | λ_em_^[b]^  (nm) | Δv ^[c]^  (cm^-1^) | ε_max_^[d]^  (M^-1^ cm^-1^) | Φ_F_^[e]^ | Brightness^[f]^ |
| --- | --- | --- | --- | --- | --- | --- | --- |
| **McL1** | PBS | 437 | 640 | 7258 | 24434 | 0.0102 | 237 |
|  | DMSO | 461 | 656 | 6448 | 27200 | 0.0842 | 2291 |
|  | MeOH | 464 | 648 | 6120 | 26300 | 0.0465 | 1223 |
|  | EtOH | 466 | 641 | 5859 | 27000 | 0.1061 | 2865 |
|  | DMF | 467 | 660 | 6262 | 27200 | 0.0525 | 1428 |
|  | ACN | 462 | 655 | 6378 | 26700 | 0.0304 | 811 |
|  | THF | 468 | 637 | 5669 | 26400 | 0.1590 | 4197 |
|  | DCM | 510 | 653 | 4294 | 27800 | 0.3001 | 8342 |
| **McL2** | PBS | 490 | 650 | 5024 | 32818 | 0.0046 | 152 |
|  | DMSO | 494 | 620 | 4114 | 57700 | 0.0045 | 259 |
|  | MeOH | 495 | 616 | 3968 | 60500 | 0.0036 | 218 |
|  | EtOH | 496 | 613 | 3848 | 62500 | 0.0087 | 542 |
|  | DMF | 497 | 610 | 3727 | 62900 | 0.0028 | 178 |
|  | ACN | 491 | 640 | 4742 | 61900 | 0.0015 | 95 |
|  | THF | 505 | 631 | 3954 | 43000 | 0.0077 | 331 |
|  | DCM | 534 | 648 | 3294 | 62900 | 0.0065 | 410 |
| **McL3** | PBS | 500 | 650 | 4615 | 34617 | 0.0019 | 66 |
|  | DMSO | 505 | 649 | 4394 | 61800 | 0.0034 | 207 |
|  | MeOH | 500 | 647 | 4544 | 65100 | 0.0030 | 195 |
|  | EtOH | 508 | 637 | 3986 | 63300 | 0.0049 | 311 |
|  | DMF | 506 | 659 | 4588 | 73100 | 0.0044 | 325 |
|  | ACN | 501 | 657 | 4739 | 65500 | 0.0021 | 135 |
|  | THF | 515 | 648 | 3985 | 45400 | 0.0048 | 219 |
|  | DCM | 530 | 657 | 3647 | 57200 | 0.0104 | 595 |

^[a]^ Peak position of the compounds (10 μM) in different polarity organic solvents with the longest absorption band in nm. ^[b]^ Peak emission position in nm, which excited at the corresponding wavelength of maximum absorption. ^[c]^ Stokes’ shift in cm^-1^. ^[d]^ Extinction coefficient in M^-1^ cm^-1^. ^[e]^ Relative fluorescence quantum yields using Rhodamine B (*Φ*_r_ = 0.31 in water) as standard, respectively. ^[f]^ Brightness calculated by *εΦ*.

**Table S3.** The DFT-calculated energy levels of singlets and triplets of **McL1**–**3** and **McL3@HSA**.

| Sample | **McL1** | | | **McL2** | | | **McL3** | | | **McL3@HSA** | | |
| --- | --- | --- | --- | --- | --- | --- | --- | --- | --- | --- | --- | --- |
| Energy level^[a]^ | S_n_ (eV) | T_n_ (eV) | S_1_-T_n_ (eV) | S_n_ (eV) | T_n_ (eV) | S_1_-T_n_ (eV) | S_n_ (eV) | T_n_ (eV) | S_1_-T_n_ (eV) | S_n_ (eV) | T_n_ (eV) | S_1_-T_n_ (eV) |
| 1 | 2.11 | 1.19 | 0.92 | 1.82 | 1.36 | 0.46 | 1.88 | 1.44 | 0.44 | 1.66 | 1.43 | 0.23 |
| 2 | 2.74 | 2.34 | -0.23 | 2.03 | 1.48 | 0.34 | 1.92 | 1.53 | 0.35 | 1.93 | 1.58 | 0.08 |
| 3 | 2.81 | 2.36 | -0.25 | 2.42 | 1.90 | -0.08 | 2.16 | 1.64 | 0.24 | 2.16 | 1.65 | 0.01 |
| 4 | 3.42 | 2.57 | -0.47 | 2.52 | 2.16 | -0.34 | 2.28 | 1.94 | -0.06 | 2.33 | 2.09 | -0.43 |
| 5 | 3.58 | 2.84 | -0.73 | 2.93 | 2.53 | -0.71 | 2.56 | 2.13 | -0.25 | 2.55 | 2.25 | -0.59 |
| 6 | 3.65 | 3.52 | -1.41 | 2.94 | 2.60 | -0.78 | 2.56 | 2.30 | -0.42 | 2.72 | 2.32 | -0.66 |
| 7 | 3.94 | 3.57 | -1.46 | 3.23 | 2.67 | -0.85 | 2.91 | 2.38 | -0.50 | 2.80 | 2.34 | -0.68 |
| 8 | 4.30 | 3.62 | -1.51 | 3.41 | 2.71 | -0.89 | 3.03 | 2.61 | -0.73 | 2.94 | 2.59 | -0.93 |

^[a]^ The energy levels of singlet and triplet states were calculated by Gaussian 16 at the B3LYP/def2-SVP level [2].

**Table S4.** The DFT-calculated spin-orbit coupling constants (SOC) of **McL1**–**3** and **McL3@HSA**.

| SOC (cm^-1^)^[a]^ | **McL1** | **McL2** | **McL3** | **McL3@HSA** |
| --- | --- | --- | --- | --- |
| ξ(S_1_-T_1_) | 0.017 | 0.085 | 0.071 | 0.235 |
| ξ(S_1_-T_2_) | 0.000 | 0.093 | 0.046 | 0.324 |
| ξ(S_1_-T_3_) | 0.049 | 0.307 | 0.079 | 0.049 |
| ξ(S_1_-T_4_) | 0.014 | 0.044 | 0.130 | 0.058 |
| ξ(S_1_-T_5_) | 0.037 | 0.092 | 0.177 | 0.980 |

^[a]^ Spin-orbital coupling (SOC) constants (ξ) between singlet (S) and triplet (T) states were derived from ORCA calculations at the B3LYP/def2-SVP level [3].

**Table S5.** Photophysical and HSA binding properties of **McL1**–**3**.

| Compd. | Λ_abs_^[a]^ (nm) | λ_em_^[b]^  (nm) | Δv ^[c]^  (cm^-1^) | ε_max_^[d]^  (M^-1^ cm^-1^) | *Φ_F_* ^[e]^ | Brightness^[f]^ | *τ_F_* ^[g]^ | *K*_r_^[h]^  (×10^7^ s^-1^) | *K*_nr_^[i]^  (×10^9^ s^-1^) | *K*_a_^[j]^  (×10^5^ M^-1^) | Bonding site^[K]^ |
| --- | --- | --- | --- | --- | --- | --- | --- | --- | --- | --- | --- |
| **McL1** | 437 | 640 | 7258 | 24434 | 0.0102 | 237 | 0.34 | 3.00 | 2.91 | / | / |
| **McL2** | 490 | 650 | 5024 | 32818 | 0.0046 | 152 | 0.42 | 1.10 | 2.37 | / | / |
| **McL3** | 500 | 650 | 4615 | 34617 | 0.0019 | 66 | 0.41 | 0.46 | 2.43 | / | / |
| **McL1@HSA** | 435 | 592 | 6097 | 22100 | 0.2176 | 4809 | 2.61 | 8.34 | 0.30 | 1.36 | 0.93 |
| **McL2@HSA** | 500 | 615 | 3740 | 35700 | 0.0371 | 1326 | 1.52 | 2.44 | 0.63 | 2. 86 | 0.95 |
| **McL3@HSA** | 505 | 597 | 3052 | 36500 | 0.0297 | 1085 | 2.88 | 1.03 | 0.34 | 3.96 | 0.93 |

^[a]^ Peak position of the compounds with the longest absorption band in nm. ^[b]^ Peak emission position in nm, which excited at the corresponding wavelength of maximum absorption. ^[c]^ Stokes’ shift in cm^-1^. ^[d]^ Extinction coefficient in M^-1^ cm^-1^. ^[e]^ Relative fluorescence quantum yields using Rhodamine B (*Φ*_r_ = 0.31 in water) as standard, respectively. ^[f]^ Brightness calculated by *εΦ*. ^[g]^ Fluorescence lifetimes in ns. ^[h]^ Radiative transition speeds (*K*_r_) in s^-1^ determined by *Φ_F_*/*τ_F_*. ^[i]^ Non-radiative transition rate constant (*K*_nr_) in s^-1^ determined by (1-*Φ_F_*)/*τ_F_*. ^[j]^ Affinty constant (*K*_a_) of pyrido cyanine assembly with HSA in M^-1^. ^[K]^ Bonding site of pyrido cyanine assembly with HSA. Unless otherwise specified, all measurements are conducted in PBS (10 mM, pH = 7.42) with the concentration of 10 μM.

**Table S6.** Simulation docking results of **McL3** and HSA (PDB: 4LB9) in different binding poses.

| Modes^[a]^ | Binding Energy  (kcal/mol) | r_msd_ (l.b.) | r_msd_ (u.b.) |
| --- | --- | --- | --- |
| 1 | -10.6 | 0.000 | 0.000 |
| 2 | -9.3 | 9.092 | 13.767 |
| 3 | -8.9 | 4.011 | 13.749 |
| 4 | -8.7 | 3.797 | 6.451 |
| 5 | -8.6 | 7.133 | 10.753 |
| 6 | -8.4 | 8.245 | 14.490 |
| 7 | -8.3 | 10.573 | 17.724 |
| 8 | -8.2 | 8.349 | 13.769 |
| 9 | -8.1 | 1.762 | 2.302 |

^[a]^ Docking stages were predicted by AutoDock Vina and results were visualized with Discovery Studio software.

**Table S7.** IC_50_ values of **McL3@HSA** under different treatment conditions.

| **Condition** | **IC_50_ (μM)** | **Photocytotoxicity index** ^[a]^ |
| --- | --- | --- |
| Dark | 243 | / |
| Light (normoxia) | 3.52 | 69.03 |
| Light (hypoxia) | 4.28 | 56.78 |

^[a]^ Photocytotoxicity index was determined by (IC_50(dark)_/IC_50(light)_) [4].

# Reference

1. Fang B, Bai H, Zhang J, Shi M, Ge Y, Wang L, Li P, Ding Y, Zhang S, Zhang C, et al. Fluorogen‐activating human serum albumin for mitochondrial nanoscale imaging. *Adv Mater*. 2025;37(35):2501849.

2. Frisch MJ, Trucks GW, Schlegel HB, Scuseria GE, Robb MA, Cheeseman JR, G. Scalmani, Barone V, Mennucci B, Petersson GA, et al. *Gaussian 16, inc., Wallingford CT*. 2016.

3. Neese F. The ORCA program system. *WIREs Comput Mol Sci*. 2012;2(1):73-78.

4. Jiang Y, Huang S, Ma H, Weng J, Du X, Lin Z, Kim J, You W, Zhang H, Wang D, et al. RNA-activatable near-infrared photosensitizer for cancer therapy. *J Am Chem Soc*. 2024;146(36):25270-25281.
